# Evaluation of randomized controlled trials: a primer and tutorial for mental health researchers

**DOI:** 10.1186/s13063-023-07596-3

**Published:** 2023-08-30

**Authors:** Mathias Harrer, Pim Cuijpers, Lea K. J. Schuurmans, Tim Kaiser, Claudia Buntrock, Annemieke van Straten, David Ebert

**Affiliations:** 1 Psychology and Digital Mental Health Care, Technical University Munich, Georg-Brauchle-Ring 60-62, Munich, 80992 Germany; 2 Clinical Psychology and Psychotherapy, Institute for Psychology, Friedrich-Alexander-University Erlangen-Nuremberg, Erlangen, Germany; 3 Department of Clinical, Neuro and Developmental Psychology, Amsterdam Public Health Research Institute, Vrije Universiteit Amsterdam, Amsterdam, the Netherlands; 4 WHO Collaborating Centre for Research and Dissemination of Psychological Interventions, Vrije Universiteit Amsterdam, Amsterdam, the Netherlands; 5 Methods and Evaluation/Quality Assurance, Freie Universität Berlin, Berlin, Germany; 6 Institute of Social Medicine and Health Systems Research (ISMHSR), Medical Faculty, Otto Von Guericke University Magdeburg, Magdeburg, Germany

**Keywords:** Mental health, Randomized controlled trial, Data analysis, Tutorial

## Abstract

**Background:**

Considered one of the highest levels of evidence, results of randomized controlled trials (RCTs) remain an essential building block in mental health research. They are frequently used to confirm that an intervention “works” and to guide treatment decisions. Given their importance in the field, it is concerning that the quality of many RCT evaluations in mental health research remains poor. Common errors range from inadequate missing data handling and inappropriate analyses (e.g., baseline randomization tests or analyses of within-group changes) to unduly interpretations of trial results and insufficient reporting. These deficiencies pose a threat to the robustness of mental health research and its impact on patient care. Many of these issues may be avoided in the future if mental health researchers are provided with a better understanding of what constitutes a high-quality RCT evaluation.

**Methods:**

In this primer article, we give an introduction to core concepts and caveats of clinical trial evaluations in mental health research. We also show how to implement current best practices using open-source statistical software.

**Results:**

Drawing on Rubin’s potential outcome framework, we describe that RCTs put us in a privileged position to study causality by ensuring that the potential outcomes of the randomized groups become exchangeable. We discuss how missing data can threaten the validity of our results if dropouts systematically differ from non-dropouts, introduce trial estimands as a way to co-align analyses with the goals of the evaluation, and explain how to set up an appropriate analysis model to test the treatment effect at one or several assessment points. A novice-friendly tutorial is provided alongside this primer. It lays out concepts in greater detail and showcases how to implement techniques using the statistical software R, based on a real-world RCT dataset.

**Discussion:**

Many problems of RCTs already arise at the design stage, and we examine some avoidable and unavoidable “weak spots” of this design in mental health research. For instance, we discuss how lack of prospective registration can give way to issues like outcome switching and selective reporting, how allegiance biases can inflate effect estimates, review recommendations and challenges in blinding patients in mental health RCTs, and describe problems arising from underpowered trials. Lastly, we discuss why not all randomized trials necessarily have a limited external validity and examine how RCTs relate to ongoing efforts to personalize mental health care.

**Supplementary Information:**

The online version contains supplementary material available at 10.1186/s13063-023-07596-3.

## Background

Randomized controlled trials (RCTs) are widely considered the “gold standard” to determine if an intervention is effective or not [1]. RCTs form a crucial part of treatment policy decisions and are regarded as one of the highest levels of evidence [2, 3]. While their primacy is not uncontested [4, 5], hardly anyone would disagree that RCTs can be an exceptionally well-suited design to study the effectiveness of some treatment or intervention.

A more practical concern is that the methodological quality of RCT evaluations, in mental health research and elsewhere, leaves much room for improvement. Back in the 1990s, Altman [6] called the poor quality of health research a “scandal”, and it has been argued that his assessment is still accurate today [7, 8]. In the past, methodological researchers have found fault with various aspects of RCT analyses; Table 1 provides an overview of commonly named problems in the literature.

**Table 1:** Common problems with statistical analyses of RCTs in (mental) health research

**Analysis steps**	**Common problems**
Missing data handling	Many systematic reviews have found that, while standards have improved in recent years [9], the missing data handling in many RCTs remains poor [9–13]: • The amount of missing data is often insufficiently reported, as is the methodology used to handle missing values. • Assumptions of the missing data handling strategy remain undiscussed (are the data assumed to be missing completely at random, missing at random, missing not at random—and why?). • Methods that are inadequate (e.g., single imputation) or based on strong assumptions (e.g., complete case analysis) are used. • Although recommended by many regulatory guidelines [14], sensitivity analyses are still underused. If sensitivity analyses are conducted, they are often not suited to test the assumptions of the main missing data handling strategy. • While often plausible, methods that model the missing not at random (MNAR) assumption are employed very infrequently and are often poorly reported.
Baseline covariate tests	Methodologists have frequently commented that baseline covariate or “randomization tests” are superfluous and that they should not be conducted [15–19].
	Nevertheless, these tests are frequently reported in RCT evaluations, and reviewers often demand them to show that the randomization “worked”. Because *P* values of these tests are often included in the baseline characteristics table, some refer to this as the “Table-1 Fallacy” [20].
Analysis model	Even when data was derived form a parallel-group RCT, researchers often calculate change from baseline and pre-post effect sizes to assess intervention effects. While widespread and often requested by reviewers, this approach does not account for regression to the mean and can produce highly misleading results [21–23].
Interpretation of results	Null (viz., *p* ≥ 0.05) results are often interpreted as showing the absence of an effect, while “absence of evidence does not imply evidence of absence” [24, 25]. This issue also pertains to negative effects, which may be uncommon but important to detect. This problem is exacerbated by the fact that (in mental health research), most trials are not even sufficiently powered to detect the main effect of the intervention [26].
	In a similar vein, “post hoc” power analyses are often conducted (or requested), e.g., to calculate the power of a trial based on its final sample size and calculated effect size (often with the intention to check if there is a “true” effect that the trial was simply not powered to detect). This approach is circular and logically flawed, since the observed power is simply a function of the *P* value [27, 28].
Reporting	There is evidence that the quality of clinical trial reports has improved substantially since journals started adopting the Consolidated Standards of Reporting Trials (CONSORT [29, 30]). Nevertheless, the reporting of RCT results in mental health research remains suboptimal [31, 32]. In the abstract, for example, trialists often fail to report methods of randomization and/or allocation concealment, or do not disclose the funding source.
	Another concern is selective reporting. Still, many trials are not preregistered in a clinical trial registry; statistical analysis plans (SAPs) provided in these registrations are often vague. This makes it easier to conceal questionable research practices such as selective outcome reporting (i.e., only reporting outcomes that fit the researcher’s objective) [33] or “outcome switching” [34] in clinical trial reports.
	Core outcome sets (COS [35]) are collections of outcomes that should be measured and reported in all clinical trials. They are a great way to ensure that endpoints are assessed consistently within a research field and using the appropriate instruments. A number of COS or related consensus papers has been developed for various mental and behavior disorders [36–41], but they remain underused. A comprehensive overview of available COS for mental health research and beyond is provided by the Core Outcome Measures in Effectiveness Trials (COMET [42]) initiative (www.comet-initiative.org).

As a remedy, it has been emphasized that researchers need to be equipped with a greater understanding of their methodology [7]. We agree with this assessment, and we believe all mental health researchers should care for and be helped to understand what makes a good RCT evaluation. In this spirit, we want to provide a non-technical introduction to some core ideas behind RCTs and how they relate to the larger topic of causal inference.

In this primer and tutorial, we discuss fundamental concepts in trial evaluations and showcase their practical implementation using the free statistical programming framework R. We focus on issues that are particularly relevant in mental health research and describe some of the avoidable and unavoidable limitations of RCTs in this field. Naturally, it is out of scope for this article to give a comprehensive view of all the intricacies in RCT analyses; instead, we want to provide a starting point and show how to get the “basics” right.

## Methods

This article consists of two parts: a conceptual primer on RCT methodology (presented here), as well as a practical tutorial in the supplementary material. The tutorial provides a practical guide on how to analyze RCTs using R, based on data of a real trial examining the effect of an Internet-based intervention for depression [43, 44]. The tutorial also presents more detailed background information on some of the concepts mentioned in the primer. Prior knowledge of R is not required to complete the tutorial.

## Results

### Potential outcomes

In mental health care, many of our research questions revolve around the *cause* and *effect* of different actions. If we administer a psychological intervention or prescribe some medication, we do so because we hope that this will *cause* our patient’s mental health to improve. If a patient starts to feel better during our treatment, we may take this as a sign that the intervention was successful. Yet in reality, we will never know the *true* impact of our actions. This is because we cannot go back in time to see how our patient would have developed had we acted differently.

This inability to go back in time and directly observe the effect of different actions is encapsulated in the “fundamental problem of causal inference” [45]. It states that a causal effect can only be shown if we compare the outcome of some action *A* to the outcome had we not taken action *A*. The problem is that only one outcome will ever be realized; the other remains a *potential* outcome that cannot be observed.

This idea is formalized in the *potential outcome framework*, which is part of the so-called Neyman–Rubin causal model (NRCM; named after Jerzy Neyman and Donald Rubin; [46, 47]). This model has become a dominant approach on how to think about causality in biomedical contexts. It also allows us to understand how and why causal inferences can be drawn from clinical trials. We will now introduce some basic concepts of this model and the notation through which they are typically expressed.

Say that we, as mental health professionals, are approached by a person *i* who currently suffers from a depressive episode. Naturally, our goal is to help that person, and this means that we have to decide which course of action is most likely to make that person feel better. Let us, therefore, assume that our outcome of interest is the depression status of person *i* several weeks into the future. We call this outcome *Y* and write *Y*_*i*_ = 1 if person *i* still suffers from depression at this later stage, and *Y*_*i*_ = 0 if not. Naturally, assuming that patients simply “have” or do “not have” depression is quite a simplification. Diagnostic manuals have often been criticized for enforcing such a false dichotomy between health and disease [48]. Yet for now, it will be helpful to think of our outcome *Y* as binary: either patients still suffer from depression after some time (*Y*_*i*_ = 1) or they do not (*Y*_*i*_ = 0).

Imagine that we have just learned of a new treatment *T* that may be just right for person *i*, and now we have to decide if we want to provide it. This means that we have two courses of action: either the treatment is provided (*T* = 1) or we decide not to provide it (*T* = 0). For this brief moment, two *potential* outcomes exist: one value of *Y*_*i*_ that we would measure in a world in which *T* was provided; and the value of *Y*_*i*_ we would measure if *T* was not provided. These potential outcomes are typically denoted as *Y*_*i*_(*T* = 1) or *Y*_*i*_(*T* = 0), respectively. Note that, using this notation, *Y*_*i*_(·) is a function; it works like a magical “crystal ball” that tells us the outcome *Y*_*i*_ depending on which action we plug into it.

For some mental health problems, it is common for patients to improve even without treatment [21]. For example, about one-third of untreated depression cases remit “spontaneously” within 6 months [49]. This means that patients’ symptoms improve so much that they no longer meet the diagnostic criteria for depression, even though they have not received any intervention. Thus, if we decide to provide the treatment *T* and patient *i* improves, this in no way guarantees that *T* actually *caused* this improvement. Maybe the patient would have also improved had we not provided the treatment. To establish the true, *causal* effect of our treatment, the two potential outcomes have to be compared with each other. This true causal effect is denoted by *τ*_*i*_:

1 $${\tau }_{i}={Y}_{i}\left(T=1\right)-{Y}_{i}(T=0)$$

Put differently, the causal treatment effect is the difference between the potential outcome if we provide the treatment, and the potential outcome if we do not provide it. If the potential outcomes do not differ, there is no “real” causal effect.

**Fig. 1:**
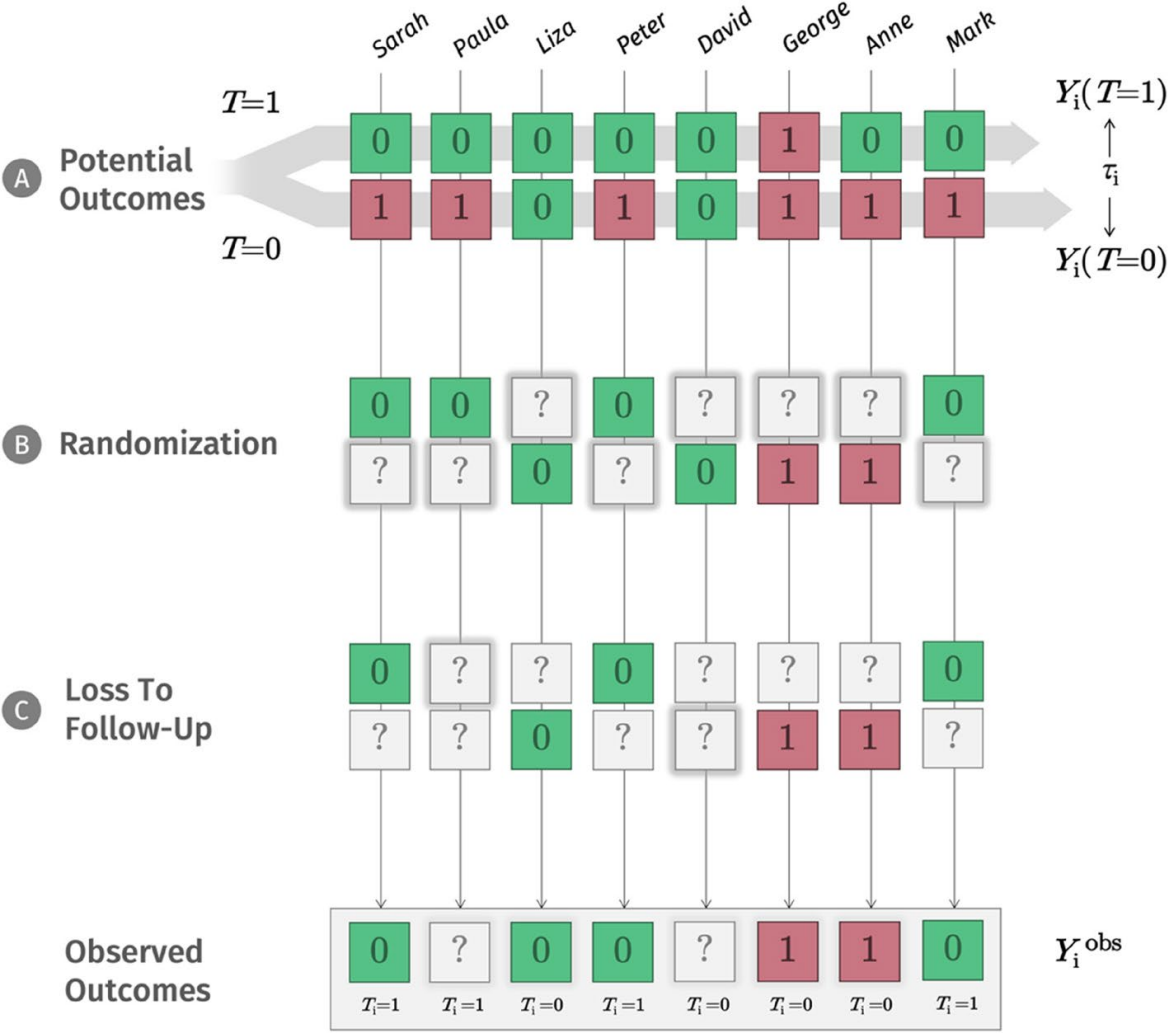
Potential and observed outcomes in RCTs. *Note*: Going from top to bottom, this diagram illustrates the hidden “machinery” inside an RCT. The top panel (**A**) shows the potential outcomes for each person in our sample if we provide a new treatment (*T* = 1) or not (*T* = 0). In our example, “0” means that a person does not suffer from a depressive episode after several weeks, while “1” means that the person still suffers from depression. The potential outcomes are hypothetical; since they are based on counterfactuals, it is impossible to observe both at the same time, and so the true causal effect *τ*_i_ of our treatment also remains unobservable. Going down one step, panel **B** shows the process of randomization, which lets chance decide which potential outcome is realized, and which one is missing (“?”). Loss to follow-up (panel **C**) adds another layer of missingness. Here, it is much less plausible that the missings are added “completely at random”. As analysts, all we end up having are the observed outcomes at the end of this process, which we need to use to estimate the unobservable causal effect *τ* on top as closely as possible. *Legend: T*_i_ = treatment allocation of patient *i* (*T*_i_ = 0 for no treatment, *T*_i_ = 1 for treatment); *τ*_i_ = causal treatment effect of patient *i*; *Y*_i_ = outcome of patient *i*: “1” (red box) if the patient still suffers from depression after several weeks, or “0” (green box) if the patient does not suffer from depression after several weeks; “?” (gray box) if the outcome was not recorded; ***Y***_i_^obs^ = observed outcomes of the trial

Usually, we are not only interested in an individual treatment effect (ITE) for one person *i*, but also in a “typical” or “overall” effect that can be expected in a patient population. This quantity *τ* is also known as the average treatment effect (ATE [50]). Panel A in Fig. 1 illustrates this. Imagine that to test if the new treatment *T* really works, we recruit a sample of eight people for which it may be suited. For each person, there are two potential outcomes, one if we provide *T*, one if we do not. The ATE *τ* is the difference between the average outcome had we provided the treatment to all individuals, 𝔼[*Y*_*i*_(*T* = 1)], and the average outcome had we given it to no one, $\mathbb E\left[{Y}_{i}\left(T=0\right)\right]$.

There is something bittersweet about this definition of the ATE. On the one hand, it gives us a “recipe” for how we can obtain the true causal effect of an intervention in a population of interest. At the same time, it shows us that, as finite beings, this true effect will always be unknown to us because it is impossible to observe the two “ingredients” that define *τ* at the same time. We cannot provide *and* not provide some treatment to the same people at the same time. This is where RCTs try to provide a solution.

### Randomization solves a missing data problem

We have now learned that if we want to confirm that some treatment is effective, we must show that there is a causal effect, defined by comparing the two potential outcomes. However, this is impossible since no two potential outcomes can ever be observed at the same time. Therefore, we need an instrument that lacking actual knowledge of *τ* at least allows us to approximate it as closely as possible.

The NRCM tells us that to draw causal inferences (e.g., “treatment *T* causes patients’ depression to improve”), we have to solve a missing data problem [51, 52]. As symbolized by panel B in Fig. 1, depending on our decision, only one potential outcome value *Y* will ever be observed. We use a question mark (?) to show that the other potential outcome will inevitably be missing from our records. In daily life, there may be countless reasons why one potential outcome is realized, while the other is “missing”. In our depression example, we could imagine that people with greater symptom severity, or higher expectations, are more likely to opt for the new treatment; but it is probably an even more complex network of reasons that determines if *T* = 1 or *T* = 0.

In RCTs, through randomization, we suspend the influence of these countless and often unknown variables by replacing it with a single factor: chance. We cannot change that one potential outcome will always be missed; but successful randomization ensures that we at least know that potential outcomes are *missing completely at random* (MCAR) for each person in our population [46, 53, 54]. The fact that outcomes are “deleted” at random has a crucial implication: it means that the average potential outcome of those receiving treatment (let us call them “group A”) and those without treatment (“group B”) become *exchangeable* [55, 56]. We would have observed a comparable distribution of outcomes even if we had somehow made a mistake and had always given the treatment to group B instead of group A.

The concept of exchangeability can be difficult to understand at first. It essentially means that the treatment status (“does person *i* receive treatment or not?”) is now completely independent of the potential outcomes of each person. Provided we have a sufficiently large sample, this allows us to get a representative cross-section of the potential outcomes we had observed if all patients had received the treatment; and it also provides us with an unbiased sample of all the potential outcomes we had observed if no patient had received the treatment.

Through randomization, we ideally achieve something in a sample that we learned was impossible to do for one person *i*: observing the outcome of some action, while at the same time also seeing the outcome had we not acted like this. The two groups have now become like real-life crystal balls for each other, where group B with *T* = 0 indicates what would have happened to group A if it had not received the treatment, and group A suggests what would have happened to group B if we had in fact provided it with the treatment. This is possible because we know that, *in theory*, the other potential outcomes still exist for both randomized groups and that the average of these potential outcomes is exchangeable with the average of the other group. At any stage post-randomization, the difference in outcome means *μ*_1_ – *μ*_2_ between the two groups can therefore be used to approximate the true causal effect *τ* of our treatment *T*.

This is an important insight that is often neglected in practice. By definition, the ATE estimated in RCTs is a *between-group* effect. In many mental health trials, it is still common to see that patients’ change from baseline is used to measure the “treatment effect” of an intervention. This is a flawed approach, even when the change scores over time in the intervention group are compared to the ones of a control group (see Table 1). There are various mathematical reasons that should discourage us from conducting change score analyses [57, 58], but they also often reveal a conceptual misunderstanding. In RCTs, we are not interested in *within-group* change over time: we want to estimate the true, causal effect of our treatment, and this causal effect is estimated by the difference between our randomized groups at some specific point in time. Naturally, many RCTs contain not only one but several follow-ups. It is also possible to include these multiple outcome assessments into a single statistical model and then estimate the overall effect of the intervention over the study period. However, even in such a longitudinal analysis, we are only interested in the average difference *between* the two groups that we observe across the different follow-up measurements, not how outcomes change from baseline *within* a single arm. A suitable method to conduct longitudinal analyses in RCTs known as mixed model repeated measures is demonstrated in the tutorial (S6.2).

Ideally, RCTs bring us into a privileged position: only by comparing the intervention and control group means at one or several specific time points after randomization, we generate a valid estimate of our treatment’s average causal effect. Within-group changes from baseline are therefore typically neither relevant nor appropriate to estimate the ATE. It should also be noted that RCTs are not the only instrument to estimate *τ*. It simply becomes much more difficult, and requires much more untestable assumptions, once (potential) outcomes are not MCAR [55, 59]. We will see this in the following section.

The NRCM also reveals another crucial point about RCTs: they work, not necessarily because randomization makes the two randomized groups perfectly identical, but because they make the potential outcomes of both groups exchangeable. This point also allows to understand why baseline imbalance tests to show that randomization “worked” are misguided, even though they are commonly seen in practice (see Table 1). The goal of randomization is to create exchangeability in the potential *outcomes* because this allows us to draw causal inferences, not to ensure that the groups have identical *baseline values* [51]. Even successful randomization provides perfectly balanced groups only *in the long run*; in our finite RCT sample, allocating treatment by chance is perfectly consistent with the idea that baseline means may also sometimes differ *by chance*. Random baseline imbalances can be relevant, but only if they occur in variables that are associated with differences in the (potential) outcomes [60, 61]. Below, we show that covariate adjustment in the analysis model can be used to address this concern.

### Ignorability

 Unsurprisingly, in practice, no such thing as an “ideal RCT” exists. In reality, we often have to deal with many additional problems, such as loss to follow-up. Panel C in Fig. 1 shows that we can think of loss to follow-up as a second layer of missingness added *after* randomization. These missings could occur for various reasons: maybe unobserved people moved to another city; they might have busier jobs; or they could have had negative side effects, leading them to discontinue treatment. In any way, on this “layer”, it is often implausible that values are simply missing completely at random. Looking at the examples above, it is clear that loss to follow-up can distort our estimate of the ATE dramatically. Imagine that all treated individuals who had negative side effects were lost to follow-up. This would introduce *selection bias* [62]: our estimates are only based on individuals who tolerated the treatment well in the treatment group, and we would overestimate the ATE.

In this moment, it is helpful to go back to the NRCM. To us, missing values due to loss to follow-up are analogous to potential outcomes: they exist in theory, we just have not observed them. Yet, to approximate them as closely as possible, we now need an assumption that is more plausible than MCAR. Here, we resort to a trick. We now stipulate that values are missing randomly, but only in a subset of our data with identical covariates ***X***. Imagine that people working a full-time job were less likely to have time for the post-assessment, and that this fully explains our missing values. This implies that, once we only look at full-time workers, the outcomes of those who provide data, and those who do not, will not systematically differ (i.e., the observed and unobserved outcomes are *exchangeable* again). If we can identify some combination of covariates ***X*** conditional on which values are randomly missing again, we speak of *ignorable* missing data [63]. This is the core idea of the *missing at random* (MAR) assumption: facing missing follow-up data, we can “rescue” our estimate of the causal effect using prognostic information captured by our observed baseline covariates ***X***. This inherently untestable assumption is crucial since many relevant imputation methods depend on it.

### Trial estimands

Arguably, the potential outcome framework we covered above is quite theoretical. It is still important to understand some of its core tenets because they define precisely when causal inferences can be drawn from data and what inherently unobservable effect RCTs actually try to get as close as possible to. In real-life RCTs, our research questions are much less abstract than that, and we now need a tool that links all this theory to the concrete analyses we should perform in our own evaluation. One way to align the theory and hands-on implementation of an RCT analysis is through so-called trial estimands.

Estimand means “what is to be estimated”. Previously, we learned that RCTs allow us to estimate the ATE caused by the treatment. This sounds straightforward, but things get more complicated once we think of the intricacies of “real-life” RCTs. How, for example, should this effect be measured, and when? In what population is this effect to be expected? What do we do if some patients do not use the intervention as intended? Trial estimands allow us to answer these questions precisely and unambiguously. They are a list of statements that describe the (i) compared treatment conditions, (ii) targeted population, (iii) handling of “intercurrent events”, (iv) measured endpoint, and (v) what population-level summary is used to quantify the treatment effect [64]. Table 2 shows a concrete example for a psychological intervention.

**Table 2:** Example of a trial estimand employing a treatment policy strategy

Attribute	Example
1. Treatment conditions	Internet-based, 8-week intervention for subthreshold depression with full access to care as usual, versus one-session psychoeducation with full access to care as usual.
2. Population	Elderly individuals (≥ 65 years) with mild depression (PHQ-9 ≥ 10) who do not fulfill diagnostic criteria of a major depressive episode according to the DSM-5.
3. Intercurrent events	Treatment policy strategy: effect regardless of treatment discontinuation (i.e., not completing all intervention sessions as intended) or use of other treatments (e.g., conventional psychotherapy or pharmacotherapy).
4. Endpoint	PHQ-9 depressive symptom severity score 8 weeks after randomization (post-test), measured regardless of intercurrent events.
5. Summary measure	Mean treatment group difference in the endpoint, expressed as a standardized mean difference (Cohen’s *d*), with the pooled post-test *SD* used for standardization.

*DSM-5* Diagnostic and Statistical Manual of Mental Disorders, Fifth Edition, *PHQ-9* Patient Health Questionnaire 9

Trial estimands play an important role in regulatory affairs and have been adopted by both the European Medicines Agency [65] and the U.S. Food and Drug Administration (FDA; [66]). They are still much less common in mental health research, but there are very good reasons to make them a routine step in each RCT evaluation [67, 68].

Estimands also allow to us understand what is *not* estimated. Imagine that we conducted an RCT comparing a new type of psychotherapy to a waitlist. If the trial is successful, we could be inclined to say that our treatment has a true causal effect, meaning that therapists should now deliver it to help their patients. Looking at the estimand, we can immediately see that this reasoning is flawed because the trial only estimated the causal effect compared to a waitlist, not compared to what therapists usually do in their practice. In this particular case, we are confusing the “efficacy” of the treatment in a waitlist-controlled trial with its “effectiveness” as a routine-care treatment [69]. Many of such misinterpretations and sweeping overgeneralizations can be avoided by honestly stating what effect our RCT analysis actually estimates.

Detailing the handling of intercurrent events (such as treatment discontinuation, or use of other treatments) within the estimand is another important part since this can change the interpretation of the ATE. Typically, a so-called treatment policy strategy will come closest to the definition we elaborated earlier: we want to estimate the outcome of our treatment compared to what would have happened had we not provided it (or provided some alternative treatment instead), *no matter* if the treatment was actually used as intended [70]. This strategy largely overlaps with what is more commonly known as the “intention-to-treat” principle: once randomized, individuals are always analyzed. This strategy is most easily implemented if we were also able to obtain follow-up data of patients who did not follow the treatment protocol [71].

As part of their RCT evaluation, many mental health researchers also conduct a so-called per-protocol analysis, in which only participants who adhered to the treatment protocol are included in the analysis. This approach is often used as a sensitivity analysis to examine the treatment’s efficacy under optimal conditions. However, this type of analysis is not supported by current guidelines because it extracts different subsets from both arms of our trial that may not be entirely comparable [72]. It is possible to estimate such an “optimal” effect of a treatment using the so-called principal stratum strategy [73], but this approach is based on many statistical assumptions and may be more difficult to implement in practice.

### Climbing up the ladder: the analysis model

To appreciate what the goal of the analysis model in RCT evaluations is, we must first go back to Fig. 1. In its totality, this visualization illustrates what is sometimes called the *data-generating process*, the “hidden machinery” inside an RCT. On top, we have the lofty realm of the potential outcomes, which exist in theory, but are never fully observable. As we proceed downwards, many of these outcomes “go missing”: first through randomization, where we know that potential outcomes are “deleted” completely at random and later through loss-to-follow-up, where the missingness mechanism is much less clear. All we RCT analysts end up with are the observed data at the very bottom of this process. The statistics we use in RCT evaluations are an attempt to climb this ladder back up.

Previously, we described that missing data, and how they are handled, play a critical role in RCTs. There is some degree of loss-to-follow-up in virtually all clinical trials, and this means that some unknown missingness mechanism will be lurking behind our observed data. Multiple imputation (MI [74]), in which several imputed datasets are created, and parameters of interest (e.g., the ATE) estimated in each of them, has now become a common approach to handle missing data in clinical trials. Through what is known as “Rubin’s rules” [75], MI allows to calculate pooled estimates which properly reflect that we can estimate the true value of an imputed variable but will never know it *for certain.*

MI is a very useful and highly flexible method, and the tutorial shows how to apply it in practice (see S4.2). Nevertheless, it is important to keep in mind that MI, like all imputation procedures, is based on untestable assumptions. Typically, MI approaches assume that data are missing at random and will only be valid if our imputation model is a plausible approximation of the true missingness mechanism [76]. In the tutorial, we also cover alternative approaches which assume that the data are missing not at random (MNAR; S4.2.3) and which are often sensible for sensitivity analyses.

When conducting an RCT evaluation, it is helpful to understand the imputation and analysis model as two sides of the same coin. Both try to approximate the true data-generating mechanism within our trial, and they should therefore be *compatible* with each other [77]. In the literature, this is also known as the *congeniality* of the imputation and analysis model [78]. We describe this concept in greater detail in the tutorial (S4.2.3).

Various statistical models can be used to estimate the ATE within RCTs [61, 79, 80], and more options are discussed in the tutorial. For now, we focus on analysis of variance (ANOVA), which remains the one of the most widely used methods to test the treatment effect in clinical trials. First published in 1935, Ronald A. Fisher’s “The Design of Experiments” laid the foundations for randomized designs, while popularizing ANOVA as a suitable analysis method [81]. This historical legacy could explain why ANOVA is still often thought of as a “specific” method for randomized experiments and as unrelated to other statistical models. In reality, ANOVA models are simply a special type of linear regression [79]. In a “vanilla” two-armed single-center RCT with one clearly defined primary outcome, the ATE can be estimated (and tested) using the following regression equation:

2 $${\widehat{Y}}=\alpha +{\beta }_{T}{T}+ {\beta }_{1}{x}.$$

where *Y* is the primary outcome of our trial (which we for now assume is continuous), $\alpha$ (the intercept) is the outcome mean in the control group, and ${\beta }_{T}$ is the coefficient estimating our treatment effect: the amount by which the expected value of *Y* is shifted up or down for the treatment group (*T* = 1; where *T* = 0 for the control group). Since we only have two groups, a *t*-test of our group coefficient ${\beta }_{T}$ will be mathematically equivalent to the *F*-test in an ANOVA. In the tutorial (S5.1.3) we show that, once the linear regression model in Eq. 2 has been fitted, we can also easily produce the *F*-test results we would have obtained from an ANOVA. If (2) were to only include the aforementioned terms, ${\beta }_{T}$ would be the simple difference in means between the two groups in our trial; this quantity is also known as the “marginal” treatment effect. Here, however, we *adjust* the estimate by including the baseline covariate *x* as a predictor (in ANOVA language, this results in an analysis of covariance, ANCOVA). Typically, *x* is a baseline measurement of the outcome variable, and it is also possible to adjust for multiple covariates, provided they were specified before the analysis (see also S5.2.1 in the tutorial).

Intuitively, one may think that, thanks to randomization, covariate adjustments are unnecessary; but there are several reasons to include them. First, “good” covariates explain variation of our outcome *Y within* treatment groups, so adjusting for them increases our statistical power (i.e., the confidence interval around ${\beta }_{T}$ shrinks [82]). Second, adjustment for prognostic covariates automatically controls for potential baseline imbalances *if they matter*: that is, when there is a disbalance in baseline covariates that are strongly predictive of the outcome [83]. Third, it is sometimes argued that covariate adjustment is helpful because it provides a *personalized* interpretation of ${\beta }_{T}$ as the predicted difference in outcomes between two patients with identical covariate values *x*, but different treatment (*T* = 0 vs. *T* = 1 [84]).

In practice, treatment decisions are made for individuals, so it is tempting to follow this interpretation. Yet, this is not the reason why we adjust for covariates. In RCTs, our goal is to estimate the mean difference between the intervention and control group from our sample, and the covariate-adjusted model in Eq. 2 just happens to allow to estimate this marginal effect more precisely, at least for continuous outcomes. In logistic regression models, which are commonly used for binary outcomes, covariate adjustment has a different effect than what we described above: the confidence intervals do not tighten up, but the value of ${\beta }_{T}$ increases instead [85]. This behavior is associated with the “non-collapsibility” of the odds ratio [86, 87], a numerical averaging failure that causes the average of conditional odds ratios (e.g., odds ratios calculated in subgroups of our trial sample) to not necessarily equal the unadjusted odds ratio that we observe in the entire sample. We explain this statistical “oddity” in greater detail in S5.2.2 in the tutorial, but the main point is that this behavior inadvertently changes our estimand. The odds ratio measured by ${\beta }_{T}$ does not estimate the ATE anymore; instead, we obtain a conditional treatment effect that is typically higher than the “original” ATE, and which depends on the covariate distribution in our trial, as well as the covariates we decide to adjust for [88, 89]. One way to deal with this problem is to fit a logistic regression model in the first step, and then use a method known as *regression standardization* to obtain the desired estimate of the effect size (e.g., an odds or risk ratio). Briefly put, this method first uses the logistic regression model with covariates to predict the outcome *Y* while “pretending” that all participants had been allocated to the treatment group. Then, it does the same assuming that everyone had been assigned to control. Comparing the means of these two counterfactual predictions, we obtain a valid estimate of the marginal effect size in our trial sample, while taking into account the covariates in our model. In the tutorial, we explain this method in further detail and show to apply it in practice (S5.2.2).

In contrast, when *Y* is continuous, the main analysis model can be used directly to calculate effect size measures. If we divide (“standardize”) the estimate of ${\beta }_{T}$ and its confidence interval in our linear regression model by the pooled standard deviation of *Y*, we obtain an estimate of the between-group standardized mean difference (the well-known Cohen’s *d*), as well as its confidence interval (see S5.1.4 in the tutorial).

There are also cases in which effect sizes and their significance play less of an important role, for example in pilot trials. Large-scale RCTs are time-consuming and costly, so external pilot trials are a helpful way to examine on a smaller scale if a new treatment can be successfully administered in the desired setting, how well the recruitment strategy works, or if patients adhere to the treatment [90]. As emphasized by the 2016 CONSORT extension [91], pilot trials should focus on a pre-defined set of feasibility objectives (e.g., “at least 25% of eligible patients can be recruited for the trial” or “at least 85% of patients are retained by follow-up”). These feasibility objectives can also serve as progression criteria to determine if a confirmatory trial can safely be rolled out [92]. Although tempting, the primary goal of pilot trials is not to estimate an effect size on clinical outcomes, or its significance, because they typically do not have the power to detect such effects.

Overall, effect sizes are an apt conclusion for our tour because they are often used to “summarize” the results of a trial. Effect sizes are also what meta-analysts use to synthesize to results of multiple studies and often an entire research field. There is a risk to “reify” effect sizes derived from RCTs, to take them as “proof” that some treatment has a true, real effect. Looking back, we can now see how fragile effect sizes really are. They are only *estimates* of an inherently unobservable quantity that will only be valid if the assumptions of our statistical analyses are correct; many of which (like the underlying missingness mechanism) are untestable. RCTs can be easily “tweaked” to show an intervention effect even when there is none [93], and for many of these design flaws, there is no direct statistical remedy at all. This is sobering, but it underlines that effect sizes and *P* values alone are not sufficient to draw valid causal inferences from RCTs.

## Discussion

It is crucial to keep in mind that clinical trial evaluations are an intricate topic and that this article barely scratches the surface. There is much more to learn about good practice in RCT analyses; a more in-depth and “hands-on” look at trial evaluations is provided in the tutorial in the supplement.

Furthermore, many problems of RCTs arise at the design stage, well before the actual analysis. Therefore, before concluding this primer, we also want to summarize a few more general shortcomings of RCTs as they are frequently observed in mental health research. Some of these problems are “human-made” and can be avoided by improving research practices and trial designs. Others are inherent limitations of RCTs in the mental health field that we have to keep in mind to draw valid inferences from them.

### Avoidable limitations

One limitation of RCTs that is both widespread and easy to avoid is the lack of prospective registration. There is a broad consensus that the protocol of a trial, including its design, planned sample size, inclusion criteria, and primary outcome should be published *before* the first patient is recruited. The International Committee of Medical Journal Editors (ICMJE) has made prospective registration a condition for publication in one of their journals, and this mandate has been in effect since 2005 [94]. Nevertheless, many mental health researchers still fail to prospectively register their trial. For example, two meta-epidemiological studies found that only 15–40% of recent psychotherapy trials were prospectively registered [95–97], and similar numbers are also found in pharmacotherapy trials [98].

Without prospective registration, analytic details can easily be tweaked to make the results of a trial appear better than they really are. One of these “methods” is known as *outcome switching*: if our original primary outcome does not show the desired effect, one can simply switch to another assessed endpoint with better results to show that the intervention “worked”. There is evidence that this and other post hoc discrepancies are widespread in mental health RCTs [33, 96, 99–101]. Naturally, the pressure of producing positive results this way may be most pronounced among trialists with financial interests in the treatment. Antidepressants are a commonly named example here [102], but similar conflicts of interest may also pertain to, e.g., the blooming “digital therapeutics” industry [103], who also need to show that their treatment is effective to sell it. The best way to circumvent these issues is to register a detailed protocol before the beginning of the trial in one of the primary registries listed by the WHO International Clinical Trials Registry Platform (ICTRP) and to analyze and report results in accordance with the original registration. The trial registration may also be supplemented with a statistical analysis plan [104, 105], which should define the trial estimand as well as the exact statistical procedures employed to estimate it. Core outcome sets (COS; see Table 1) should also be included at this stage to ensure that psychometrically valid instruments are used and to make it easier to compare the results to other trials.

A related problem is *allegiance bias*. Even without any obvious financial interests, some trialists may feel a strong sense of commitment to the treatment under study, for example because they have contributed to its development. There is a substantial body of research, especially for psychological treatments, that this type of allegiance can lead to inflated effect estimates in RCTs [106–109]. Allegiance biases can occur through different pathways. Trialist may, for example, provide better training or supervision for the personnel administering their “preferred” treatment, or they may simply have more experience in implementing it [93]. In psychotherapy research, for instance, non-directive counseling is often used as a control condition to which new interventions are compared to. Since the researchers “favor” the new treatment, the non-directive intervention is frequently implemented as an “intent-to-fail” condition [110]. This is in contrast to empirical findings which show that non-directive supportive therapy is an effective treatment in its own right and that its purported inferiority to other psychotherapies may be caused by allegiance bias [111]. One way to prevent allegiance bias is to conduct an independent evaluation by researchers who have not been involved in the development of any of the studied treatments. Guidelines such as the Template for Intervention Description and Replication (TIDieR [112]) also remain underutilized in the mental health field, but can help to clarify the components of interventions or active control conditions, and how well they may compare to other trials.

Another common weak spot are the control groups used in mental health trials. For psychological interventions, waitlists are still one of the most frequently used comparators to determine the effectiveness of the treatment. An advantage of waitlist controls is that they allow to provide the intervention to all recruited participants; patients in the control group just have to wait for it until the end of the trial. However, there is evidence that waitlists may function as a type of “nocebo” in clinical trials: since patients know that they will receive an intervention soon anyway, they may be less inclined to solve their problems in the meantime [113–115]. For treatments of depression, for example, we know that patients on the waitlist typically fare worse than under care as usual and even worse than patients who receive no treatment at all [116, 117]. In this primer, we learned that the causal effect of a treatment can only be defined in reference to some other condition. Thus, to find out if our intervention is really beneficial to patients, we must choose a *plausible* comparator for our research question. This could be, for example, a care as usual group, or another established treatment for the mental health problem under study.

A last avoidable issue of mental health RCTs concerns their sample size. It is commonly understood that the number of participants to be included our trial should be determined in advance using a power analysis [118]. Nonetheless, there is evidence that most mental health trials are woefully underpowered. A recent meta-review found that RCTs in mood, anxiety, and psychotic disorder patients had a median power of 23%, which is far below the accepted level of 80% [26]. This finding is concerning because it implies that most trials in the field are unable to attain statistically significant results for the effect they try to measure. This opens the door for a host of systemic issues that we already discussed above: selective publication, reporting bias, and data dredging, which can all be read as attempts to squeeze out significant effects from studies that do not have the sample size to detect them in the first place. Obviously, clinical trials are costly and most trialists recruit such small samples for logistic reasons, not on purpose. Yet, in this case, it may be helpful to shift to trial designs that make more efficient use of the available sample, such as adaptive [119, 120], fractional factorial [121], stepped wedge [122], or pragmatic trial [123] designs. Failure to recruit the targeted sample size remains a widespread issue in both pharmacotherapy [124, 125] and psychotherapy trials [126–129]. Sometimes, it may still be impossible for trialists to reach the required sample size established via power analysis, but the resulting lack in statistical power should then be clearly named as a limitation of the trial. Naturally, a much better approach is to identify uptake barriers beforehand. Most reasons for recruitment failures have been found to be preventable, and pilot trials are a good way to assess how difficult it will be to enroll patients in practice [130].

### Inherent limitations

There are also limitations of RCTs that are intrinsic to this research design or at least difficult to avoid in mental health research. One example is blinding. Pharmacological trials are typically designed in such a way that patients remain unaware of whether they are receiving the medication or a pill placebo. Similarly, clinicians who evaluate the outcomes are also kept blinded to the treatment assignments. Such double-blinded placebo-controlled trials are considered one of the strongest clinical trial designs, but even they can fail, for example if patients and raters recognize the side-effects of the tested medication [131]. Conducting a double-blinded trial of a psychological intervention is even more challenging and seldom attempted in practice [132]. This is because patients will typically be aware if they were assigned to a placebo condition designed to have no therapeutic effect or a “bona fide” psychological treatment.

Often, it is not easy to define for what exact placebo effects we should control for in RCTs of psychological interventions and what control groups can achieve this without breaking the blinding. For medical devices (e.g., digital mental health interventions), the U.S. FDA recommends using “sham” interventions to control for placebo effects (i.e., interventions that appear like the tested treatment, but deliver no actual therapy); but also acknowledges that constructing such control groups can be difficult [133]. Some authors have argued that the idea of a blinded placebo control does not apply to psychological interventions altogether, since both can be regarded as treatments that work solely through psychological means [134–138]; and that placebo controls should therefore be abandoned in psychotherapy research [136]. This conclusion is not uncontroversial, and others contend that some types of placebo controls can be helpful to test the “true” effect of psychological interventions [139].

Another limitation of RCTs relates to the ongoing efforts to “personalize” mental health care and to explore heterogeneous treatment effects (HTE [140–142]). Randomized trials are an excellent tool to determine the average effect of a treatment. Yet, in practice, we do not treat an “average” patient, but individuals. For mental disorders such as depression, various treatments with proven effects are available, but none of them work sufficiently well in all patients, and many people have to undergo several rounds of treatment until an effective therapy is found [143]. Thus, many researchers want to better understand which treatment “works for whom” and reliably predict which person will benefit most from which type of treatment.

In this primer, we already described that we will never know the true effect that a treatment had on an individual, since this effect is defined by counterfactual information. As described by Senn [144], this has crucial implications once we are interested in determining “personalized” treatment effects. Sometimes, the fact that some patients’ symptoms improve strongly while on treatment, whereas others do not improve, is taken as an indication that the treatment effect must vary among individuals. If we develop a model that predicts the “response” to treatment based on pre-post data alone, we implicitly follow the same rationale. Looking at panel A in Fig. 1, we see that this is a deeply flawed logic, because we do not consider how individuals would have developed without treatment. Contrary to common belief, meta-analyses of variance ratios suggest that the improvements caused by antidepressants are mostly uniform across patients [145], while a higher degree of HTE may exist in psychological treatments [146].

At first glance, RCTs may seem like a more natural starting point to examine HTE because they are based on a counterfactual reasoning. Indeed, subgroup and moderator analyses are often used in RCT evaluations to assess if treatment effects differ for patient groups. The problem is that RCTs are typically not designed for these types of analyses. Most RCTs are barely sufficiently powered to detect the average treatment effect, let alone to provide a usable estimate of the effect in specific patient subgroups [88, 147]. This problem becomes even more severe if we make these subgroups even smaller by prognosticating “individualized” treatment benefits for patients with an identical combination of baseline characteristics, as is typically done in clinical prediction modeling [148, 149]. Several methods have been proposed to deal with this limitation; for example to examine effect modification in individual participant data meta-analysis (IPD-MA), in which many RCTs are combined into one big data set [150] or to develop causally interpretable models in large-scale observational data [151].

A similar problem is that RCTs can show *that* a treatment works, but they typically cannot reveal the mechanisms that make it effective. Unfortunately, more than a single RCT is usually needed to understand which components generate the effects of an intervention. For example, although numerous RCTs have demonstrated that psychotherapy is effective, there is a decades-long debate about its working mechanisms that has not been resolved until today [152].

A last inherent limitation of RCTs concerns their “generalizability” and “transportability” [153]. By generalizability, we mean that results within our study sample can be extended to the broader population from which our study sample was drawn. Transportability means that results of our trial also translate to another (partly) different context. A frequently voiced criticism of RCTs is that, although they have high internal validity, they often lack external validity [154–156]. Treatments in RCTs are often delivered in a highly standardized way and under tightly controlled conditions. This may not accurately reflect routine care, where healthcare providers may have much less time and resources at their disposal.

If this is valid criticism depends on the goals of trial. For a newly developed treatment, it may be more important to first show that it has a causal effect under optimized conditions, while tightly controlling for potential biases. On the other hand, pragmatic trials can be conducted to examine the effects of more established treatments under conditions that are closer to routine care and therefore also have a greater external validity [123]. Please to prioritize “real-world evidence” over RCTs have also been criticized on the grounds that high internal validity of randomized evidence is a prerequisite of external validity [157, 158] and that regulators should rather focus on reducing the bureaucratical burden associated with conducting RCTs [158].

A related concern involves the fact that RCTs often have very strict inclusion criteria, and therefore their findings may not be transportable to the actual target population [159]. Many trials exclude, e.g., individuals with subthreshold symptoms or comorbidities, or they may fail to recruit from minority groups and hard-to-reach populations [160–163]. Furthermore, RCTs only include patients who actively decide to participate in such a study, which means that trial samples can often be very selective. This means that a treatment may be effective in a homogenous trial sample, but less so in the more diverse target population in which it should be implemented.

Representativeness is an important consideration in clinical trial design, but it is not automatically true that randomized evidence from more restricted samples is not transportable to another population. This may only be the case if there is heterogeneity of treatment effects [153]. We mentioned that, even though it might look different at first, the true effect of some treatments can be quite homogeneous. If there are no strong effect modifiers, there is also no strong reason to believe that the treatment will have a substantially different effect in populations with varying patient characteristics. In this case, the ATE of our RCTs also provides an internally valid estimate of the effect we can expect in the target population. Of course, there are many scenarios in which HTE are indeed plausible. Then, we have to use methods that extend beyond the trial data to accurately estimate the causal effect of our treatment in a different target population of interest [164–166]. Mueller and Pearl [167] make the compelling case that RCT and routine care data, when combined, can allow to make more informed decisions about the individual risks or benefits of a treatment that may remain undetected when looking at RCTs alone. This underlines that experimental and observational studies both have their place in mental health research and that we obtain better inferences if various sources of data are considered—a practice that some refer to as “data fusion” [168].

This concludes our journey through some of the pitfalls that we should keep in mind when we evaluate and interpret the results of an RCT. We hope that this primer and tutorial delivered some helpful insights for mental health researchers. More importantly, we also hope our introduction illustrates that trial methodology is a fascinating topic worthy of further exploration.

### Supplementary Information

**Additional file 1.**

## Data Availability

All material used to compile the tutorial presented in the supplement is openly available on Github (github.com/mathiasharrer/rct-tutorial).
