# Supplementary material for: Evaluation of randomized controlled trials: a primer and tutorial for mental health researchers
Source: Trials. 2023 Aug 30;24:562. doi: 10.1186/s13063-023-07596-3 (PMC10469910; doi:10.1186/s13063-023-07596-3)
Supplement: Supplementary file 1 — Additional file 1. [file 13063_2023_7596_MOESM1_ESM.pdf]

Evaluation of Randomized Controlled Trials Using R: A Tutorial for Mental Health Researchers

Mathias Harrer†, *Pim Cuijpers*†, *Lea K. J. Schuurmans*, *Tim Kaiser*, *Claudia Buntrock*, *Annemieke van Straten*, *David Ebert*

†shared first authors

Summary. This tutorial aims to serve as an accessible introduction to the evaluation of randomized controlled trials (RCTs) in mental health research. Based on example data of a real-world clinical trial, we will cover essential steps of RCT analyses and demonstrate their implementation using the statistical programming language R.

PART 1 | Introduction

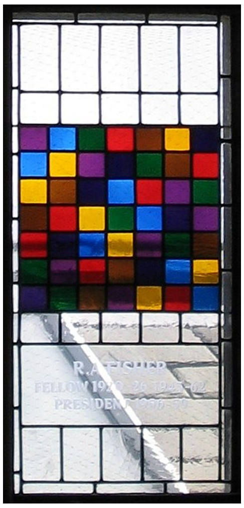

In the following pages, we give a hands-on introduction to the analysis of RCT data using R. A conceptual understanding of essential statistical methods is provided, and we then showcase how these methods can be applied in real-world examples.

An overview of the covered topics is presented on the following page. We show how to conduct a descriptive analysis of RCT data, how to deal with study dropout and other types of missing data, and how to assess if a treatment had an effect on continuous or binary endpoints in our study. We also discuss multiplicity issues in clinical trials, how to incorporate longitudinal data and provide tips on the reporting of RCT results.

The tutorial is written specifically for mental health researchers. We assume some familiarity with common research questions in this field; as well as a basic knowledge of fundamental statistical concepts (i.e., means, standard deviations, correlation, P values). Mathematical notations will be used at times throughout the tutorial, but rest assured that we will take the time to explain the meaning of each symbol, and focus mainly on the key message conveyed by each formula. Mathematical notations often allow us to precisely describe the concepts we are talking about, and having seen those formulas also makes it easier to understand more advanced texts you may want to read further down the line.

Some knowledge of R will certainly be helpful to complete the tutorial but is not required. In the first section, we describe how to install R, as well as a computer program that makes working with R much more convenient. In the first part, we provide a gentle introduction to R itself and cover operations needed throughout the rest of the tutorial.

Figure 1. The image to the right shows a stained-glass window at the Gonville and Caius College, Cambridge. It commemorates Ronald A. Fisher, whose works *Statistical Methods for Research Workers* (1925) and *The Design of Experiments* (1935) laid the groundwork for much of modern statistics. Fisher's works had a monumental impact because they provided researchers with the statistical tools needed to draw inferences from their experiments. Many methods introduced by Fisher, such as analysis of variance, remain relevant in the analysis of RCTs to this day.

The window displays a 7-by-7 *Latin square*, an experimental design that Fisher used to test the yield of fertilizers while working at the Rothamsted Experimental Station (Lehmann, 2011). While undoubtedly one of the most important statisticians of all time, Fisher was also a staunch supporter of eugenics, which is why the window was removed in 2020 (Busby, 2020). *Schutz/CC BY-SA 2.5*

Contents

Evaluation of Randomized Controlled Trials Using R: A Tutorial for Mental Health Researchers 1

PART 1 | Introduction 1

PART 2 | Preparation 3
2.1 | R and RStudio 3
2.2 | Packages 4
2.3 | Importing Data 6
2.4 | Functions 7
2.5 | Objects 9
2.6 | Operators 11
2.7 | Slicing & Indexing 13

PART 3 | Descriptive Analysis 16

PART 4 | Missing Data 21
4.1 | Missing Data Mechanisms 22
4.2 | Multiple Imputation 26
4.3 | Rubin's Combination Rules 44
4.4 | Functional Programming 48

PART 5 | Effectiveness Analysis 49
5.1 | Continuous Endpoints 49
5.2 | Binary Endpoints 61

PART 6 | Miscellaneous Topics 77
6.1 | Multiple Testing 77
6.2 | Longitudinal Data 79
6.3 | Reporting & Reproducibility 84

PART 7 | Further Reading 87

Test Your Knowledge: Solutions 88
R Package Information 89

Data & Downloads 89

Errata & Corrections 89

References 90

Index 98

PART 2 | Preparation

In this section, we will be discussing all the steps that are required to set up your computer for the analyses we present in this tutorial. In particular, we will be introducing the statistical programming language R, the technicalities of which can often serve as a first obstacle for aspiring RCT analysts.

For many mental health researchers, working with R represents the first-ever exposure to a programming language, and we will cover what distinguishes R from Graphical User Interface (GUI)-based statistical software, which is still frequently used in practice. While this is sometimes exaggerated, R does indeed have a steep learning curve and requires continuous practice to become proficient in. While it is not possible to provide an in-depth introduction into R, we will describe how to set up an R environment on a personal computer and cover some fundamental operations that are needed on a day-to-day basis.

If you are an experienced R user, most of the following information will be nothing new and this section can therefore be skipped. Nevertheless, it might be helpful to go through this section anyway as a brief refresher.

The Iceberg Metaphor

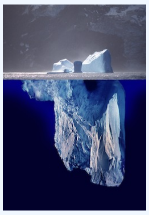

R, like any programming language, has to be actively learned and used to become an expert. Frustration is a natural part of this process. Especially in the beginning, R might often feel unnecessarily cumbersome, especially if users are already accustomed to working with more "user-friendly" GUI-based software.

We all know that the greatest part of an iceberg lies below the water's surface. Likewise, when learning R, it can take some time and practice until one sees the progress one has made. Learning R is still worth the effort: it provides us with arguably the most comprehensive, commonly used, and up-to-date statistical toolbox for our RCT analyses to date; and it is completely open-source and free.

Uwe Kils/CC BY-SA 3.0

2.1 | R and RStudio

To begin this tutorial, we first have to install the right version of R for our operating system (i.e., Windows or Mac). R comes in different versions, and it is advisable to always install the latest version. At the time we are writing this, the latest version of R is 4.2.2.

Besides R, we also install a so-called *integrated development environment* (IDE). IDEs are essentially computer programs that make working with programming languages like R more convenient. One of the best options for R specifically is **RStudio**, which can be downloaded here. *Posit*, the company behind RStudio, also offers a professional version but installing the free version is more than sufficient. RStudio is helpful because it is designed specifically for R. It makes it easier to write and execute code, as well as handle data, packages, and outputs.

R versus RStudio
R and RStudio are not identical: RStudio simply makes working with R easier; to use RStudio, R has to be already installed on our computer.

When opening RStudio, we are presented with a window with three *panes*:

- **Console**: The console is the "heart" of RStudio: here, R code can be typed in and can be executed by pressing the Enter key.

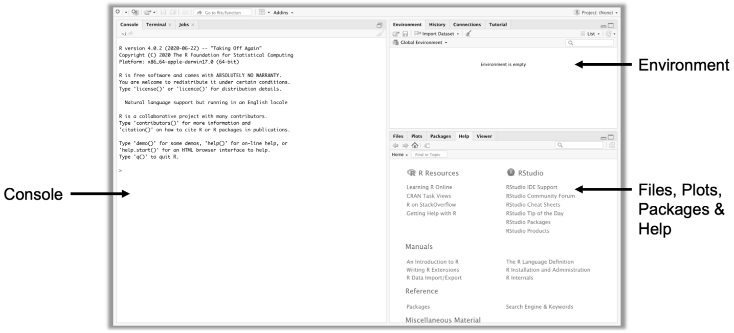

- **Environment**: All objects that we define in R are displayed here.
- **Files, Plots, Packages, and Help**: This pane fulfills several functions: it displays files and folders on our computer, graphs, and installed packages. The help pages are also displayed here.

A fourth pane, the *editor*, opens when a new R file is created (by clicking, in the toolbar on top, on "File" > "New File" > "R Script"). R scripts in the editor can be used to store important code. The scripts can be saved later via "File" > "Save" in a file with the extension `.R` on the computer.

R code is always executed in the console, and the editor is only used to write and store code. To execute code in the editor, we must pass it to the console. To do this, we have to select the relevant code chunk with our mouse, and then either hit "▷ **Run"** in the upper right corner of the editor or the key combination `Ctrl/ Cmd + Enter`.

2.2 | Packages

One of the great strengths of R is that it provides functionality for almost any statistical method imaginable. In contrast to much other software, R is not restricted to the functions and tools that its developers originally created for it. Being a full programming language, experts all around the world can write extensions for R, so-called *packages*.

Packages represent a collection of R *functions* that may be useful for a particular problem or application. For example, the package The `ggplot2` package (Wickham, 2016) provides functions for creating graphs. Packages can be developed by experts around the world and then downloaded and used in R by anyone. The *Comprehensive R Archive Network* (CRAN) alone currently lists more than 16000 installable packages.

Some functions, operators, data sets, and plotting tools are already "pre-installed" in R. Many of these functions have existed since R was first developed in the early 1990s. These functionalities are collectively referred to as **Base R** and can be used without having to install additional packages. Some packages, such as `stats` or `MASS` (Venables and Ripley, 2002), are also automatically installed with the first installation of R, and are therefore considered "Base R" too.

Besides Base R, there is the so-called **tidyverse** (Wickham et al., 2019). This is a selection of packages, all newer than Base R, which aim to simplify the manipulation and visualization of data using R. Three examples (two of which we also use later in our hands-on analysis) are:

- **`ggplot2`**: A package to generate various types of graphs and visualizations. The package can also create highly advanced visualizations, as showcased by the R Graph Gallery. However, in this tutorial, we will keep things simple and only use the plotting tools that are already pre-installed in R (see e.g. Chapter 5.2.1).
- **`dplyr`**: A package that contains helpful tools to manipulate data. Some basic familiarity with this package is highly recommended because it makes working with imported data (including RCT data) using R much more convenient. We will cover a few helpful functions included in `dplyr` in the next sections.
- **`purrr`**. A package to easily implement *functional programming* in R. This package is particularly helpful when we have to work with multiple versions of a data set at the same time, which is

a common problem in RCT analyses that employ multiple imputation. Later in this text (), we will discuss the core functions of this package in greater detail.

Packages of the tidyverse are widely used in the R community and are particularly beginner-friendly. Therefore, the `tidyverse` is the first package we will be installing in this tutorial.

The *Comprehensive R Archive Network*, or **CRAN**, is an online package repository maintained by the R Foundation. CRAN hosts the bulk of R packages we need on a daily basis, which we can install on our own computer using the `install.packages` function. To let CRAN know which package we want to have installed on our computer, we have to provide its name in brackets. In our case, the first function we have to execute using R is:

```
install.packages("tidyverse")
```

In other words, we have to type in the code above in the Console and then hit Enter. Please also note that R is **case-sensitive**, so please make sure that all letters in your call are lowercase, like in the box above. After hitting Enter, the console will print out information on the progress of the installation. Once the installation is complete, the `tidyverse` package will be added to your *system library* and will appear in the *Packages* pane, typically in the bottom-right of your RStudio window.

To be able to actually *use* functions included in a package, we have to load a package from the system library first. This is achieved using the `library` function:

```
library(tidyverse)
```

Also note how, in the call to `install.packages`, we used quotation marks (`"tidyverse"`), while, for the `library` function, this is not necessary. Another important thing to remember is that packages only have to be *installed* once, but that they typically have to be loaded from the library every time we restart R.

Installing & Loading Packages: A Few Additional Tips

- The `install.packages` function is usually not included in R scripts: when scripts are shared with others, it is considered "unseemly" to change settings on other people's computers; but this is essentially what running `install.packages` does.
- When writing an R script, it is recommended to add all packages that have to be loaded to execute it (the so-called *dependencies*) at the beginning of the script, using the `library` function.
- Some functions with the same name are included in different packages, which can lead to unnecessary confusion or potential errors in the code once these packages are loaded at the same time. The following notation can be used to specify exactly from which package the function should be taken: `package::function`. For example: `ggplot2::ggplot` means that the `ggplot` function from the package will be used.

The `tidyverse` will not remain the only package we will be using as part of this tutorial. Every time we import a new package using the `library` function, make sure you already have this package in your system library, and, if this is not the case, you first have to install it using `install.packages` in the same way we did before.

2.3 | Importing Data

Naturally, before we can apply any functions to it, we first have to **import** our data into R. Especially for beginners, this is often easier said than done. R works with so-called *working directories*; a folder on our computer which R can access to import data sets that have been saved there, and into which new files can be written. For now, let us create a new working directory on our computer that already contains all the example RCT data that we will be analyzing later on:

1. Download the .zip folder containing all the required example data **here**.
2. Save the unzipped folder `rct-tutorial` in your preferred location.
3. In RStudio, find and open the newly created folder within the bottom-right *Files* pane. The folder should contain our example data set `data.xlsx`.
4. Click on the gear wheel on top of the *Files* pane and then on *Set As Working Directory* in the drop-down menu.

The currently opened folder `rct-tutorial` is then set as the working directory within R. Setting the working directory within the user interface provided in RStudio will typically be most convenient, but it is also possible to do this directly in the console using the R function `setwd`. Within this function, we have to provide the path to the folder we want to set as the working directory, e.g. `setwd("~/Documents/rct-tutorial")`.

Once the working directory is set up, we can import our data. For novices, the most intuitive way to do this is to **click** on the file we want to import (in our case `data.xlsx`) in the bottom-right *Files* pane. Then, after clicking on *Import Dataset* in the drop-down menu, a preview of the data set pops up. If we are satisfied with the current settings, clicking on *Import* will import the data into R as an object with the name `data`. To confirm that the import was successful, one can have a look at the *Environment* pane in the top right corner of RStudio: here, the data set should be listed as an object `data` with 546 observations and 21 variables.

Another option is to import the data set via code. If, like in our case, the data set is saved as an MS *Excel* file, the `openxlsx` package (Schauberger and Walker, 2021) has to be loaded first. Then, we can use the `read.xlsx` function to import the data. Since our working directory is already set, we only have to provide the file name in parentheses:

```
library(openxlsx)
data <- read.xlsx("data.xlsx")
```

A Few Notes On *Excel* Files

As we have seen above, importing MS *Excel* files into R is not too complicated and it, therefore, makes sense to prepare RCT data as .xlsx files before analyzing them using R. Nevertheless, there are a few **"Dos and Don'ts"** when preparing *Excel* sheets for the import (Harrer et al., 2021, chap. 2.4):

- When preparing the *Excel* sheet for R, the names of the spreadsheet require special attention. If you have already named the columns of your spreadsheet appropriately in *Excel*, you can save a lot of time later because your data does not have to be converted with R. One can "name" the spreadsheet columns simply by writing the name of the variable in the first row of the column; R will then automatically recognize that this is the name of the column.
- Column names should not contain spaces. To separate two words in a column name, you can use underscores or periods (for example, "column_name").
- It doesn't matter how the columns are arranged in your *Excel* spreadsheet. They just need to be labelled correctly.

- It is also not necessary to format the columns (e.g., add cell colors or make some of the text bold). When you enter the column name in the first row of your spreadsheet, R will automatically recognize it as the column name.
- It is important to know that the import may distort special characters such as ä, ü, ö, á, é, ê, etc. You may want to convert these to "normal" letters before proceeding.
- Make sure that your *Excel* file contains only one sheet.
- If you have one or more empty rows or columns that used to contain data, make sure you delete those columns/rows completely, as R may think those columns contain (missing) data and import them as well.

2.4 | Functions

Now that we have successfully imported our data set, we can finally learn a bit more about R itself. Naturally, in this tutorial, we can only cover some of the basics, and we will focus on common operations that are needed to complete the rest of the tutorial. We will barely scratch the surface of what there is to know about R and its intricacies, but more in-depth introductory texts thankfully exist (Navarro, 2013; Wickham and Grolemund, 2016), and are highly recommended to novices.

We begin our tour of the fundamentals in R programming with one of the language's core constituents: *functions*. Functions are at the heart of R, and, while they can vary drastically in their complexity, they are all based on the same idea: we provide the function with some **input**, the function uses these inputs to perform some operation and then provides us with the **output**.

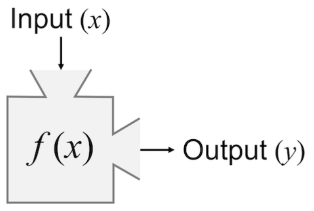

Simplified representation of a function.

Functions allow to run of pre-defined operations using R. There is a parallel between the mathematical notation of a function, $f(x)$, and the way we write functions in R:

```
function(argument_1 = value_1, argument_2 = value_2, ...)
```

We also see this logic at play when looking at, for example, the square root. Mathematically, we can write the square root as a function based on some number x, so $f(x) = \sqrt{x}$. In R, this turns into `sqrt(x)`; where x can be any kind of number (e.g. `sqrt(x=4)`). This input x to the function is also known as a *function argument*, and functions in R can also have more than one argument.

R is sometimes called a **"functional" programming language**. While this is not strictly true, functions do indeed play a greater role in R compared to other, more "object-oriented" programming languages, such as Python or Javascript.

Time to make this more concrete with a few hands-on examples. If we want to know, say, the square root of 9, we can use the following code:

```
sqrt(9)
```

```
## [1] 3
```

Or we can also use the max function to find the maximum recorded age of all participants in our imported data set data:

```
max(data$age)
```

```
## [1] 81
```

Also, note what happens if we want to calculate the mean value of the cesd.1 variable in our data set:

```
mean(data$cesd.1)
```

```
## [1] NA
```

In R, NA encodes that a value is missing. The result of the first line of code is **"not available"** (NA) because some values in cesd.1 could not be recorded at post-test, and thus the mean value can not be calculated. By setting the additional argument na.rm to TRUE, the mean is calculated using only the observed values, and we are presented with a sensible output again:

```
mean(data$cesd.1, na.rm = TRUE)
```

```
## [1] 19.74169
```

This is certainly a helpful argument; but how should one know that it exists in the first place? Some functions in R contain countless additional arguments, and it is impossible, even for highly experienced users, to know them all by heart. This problem is solved by the **R Documentation**, which provides a detailed description for each function. This documentation can either be accessed via the bottom-right *Help* pane in RStudio; or by running ?function_name in the console, for example ?mean.

Please also note that documentation entries of functions are written by the respective package developers themselves. Therefore, they may sometimes not be as informative or beginner-friendly as one may have hoped for. Outside RStudio, the R documentation can also be viewed in each browser via rdocumentation.org or rdrr.io.

Default Arguments & Position Matching

Default Arguments are arguments of a function whose value is predefined and used automatically. These arguments therefore only need to be added to the function call if their values explicitly deviate from the default settings. Default values of a function can be viewed in the *Usage* section of each R Documentation entry. If we open the documentation for the mean function, for example, we are presented with the following usage description:

```
mean(x, trim = 0, na.rm = FALSE, ...)
```

Another important aspect to keep in mind when writing functions in R is **position matching**. If we provide inputs to a function without specifying the argument names, R will automatically match the provided value with the position in which an argument is defined in the function's *Usage* section. In practice, this means that we can sometimes leave out the argument name within a function call, as long as the argument order is correct. Position matching also explains why running sqrt(x = 4) and sqrt(4) yields the same result: in both calls, 4 is used as the first argument.

2.5 | Objects

Objects can be seen as the counterpart of functions in R. Previously, we learned that functions require an input x to perform some operation, after which the output y is returned. In R, these inputs and outputs are usually stored as objects.

Before we can use objects in R, we have to assign them a variable name. This is achieved using the **assignment operator** `<-`. Imagine that we want to store some person's birth year in R. To do this, we have to assign this year (e.g. 1985) to a meaningful variable name. Here, we choose the variable name `birth_year`. We can then assign the year to this variable name using the following code:

```
birth_year <- 1985
# Call the saved object "birth_year"
birth_year
```

```
## [1] 1985
```

We have now assigned a new variable, which is saved in R. As we have seen, this object can be called directly from the console by its variable name, after which its value is returned. Also note, that this is not the only way to assign variable names in R; we could have also used any of the examples below, with the same effect:

```
1985 -> birth_year
birth_year = 1985
assign("birth_year", 1985)
```

Once objects have been assigned to a variable name, they are displayed in the *Environment* pane in the upper right corner. This means that the object is (temporarily) stored in our programming environment, and is available for further operations. Also note, that existing objects can be overwritten; running `birth_year <- 1990`, for example, would change the value of `birth_year` to 1990, and the previous year is irrevocably lost. Instead of overwriting, the `rm` function can be used to delete objects from the environment altogether, e.g. `rm(birth_year)`.

> **!** **Variable Names: Dos & Don'ts**
> Object names must start with a letter and can only contain letters, numbers, underscores and dots. It is advisable to remain consistent when naming variables: e.g. always `separate.words.with.dots` or `useCamelCase`.

After this general introduction to objects and how we can assign them, let us now explore some of the core objects **classes**, or specific "types" of objects that we can define using R.

We begin with **vectors**, which can be any type of value (e.g. numbers, words, factor levels). If a vector only consists of one number, we speak of a *scalar*. Vectors can be defined using the `c` (concatenate) function:

```
vector <- c(8, 74, 1, 6)
```

Vectors come in different "flavors". In `numeric` or `double` vectors store data as numbers (e.g., the age of several people); `character` vectors can store words and/or letters; `logical` vectors are special because they can only display if a condition is `TRUE` or `FALSE`. Like their `numeric` counterpart, `factor` vectors also store information as numbers, but each number represents a distinct factor level (e.g. 1 = low, 2 = medium, 3 = high).

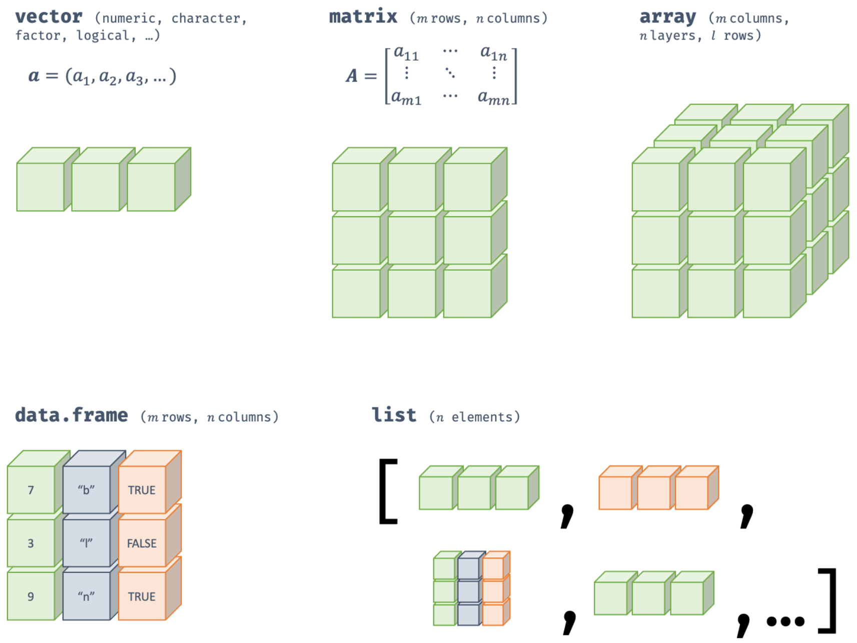

Object classes in R.

In R, these vector "flavors" can be checked using the `class` function. For example, we can check the class of the age vector in our data set data that we imported previously:

```
class(data$age)
```

```
## [1] "numeric"
```

If we want to check all the vector classes in our data set `data`, we can use the `glimpse` function included in the tidyverse:

```
glimpse(data)
```

```
## Rows: 546
## Columns: 21
## $ id          <chr> "id_1", "id_2", "id_3", "id_4", "id_5", "id_6", ...
## $ badssf.2    <dbl> 18, NA, 41, 23, 3, 38, 20, NA, 30, 19, 21, 27,  ...
## $ badssf.1    <dbl> 20, NA, 42, 18, 30, 48, 27, NA, 22, 28, 30, 32, ...
## [...]
```

Besides vectors, one of the most commonly used object types are **data frames**. Data frames are used to collect multiple vectors within one object. They essentially function like tables: for each row entry m, there are values of various variables n. Data frames can be built out of several vectors with the same length. In contrast to a similar object type, the **matrix**, different vector classes (numeric, logical, character, ...) can be bundled together in data frames.

As an example, let us build our own data frame from scratch. Imagine that, for some reason, we want to record the name and age of some of our co-workers, and if they like liquorice. Therefore, we first define three vectors which contain this information:

```
name <- c("Sarah", "Paula", "Antonia", "Peter")
```

```
age <- c(45, 31, 31, 27)
likes_liquorice <- c(FALSE, FALSE, FALSE, TRUE)
```

Now that these three same-length vectors are defined, we can bind them together in a data frame, using the `data.frame` function. Here, we call this data frame `df`.

```
df <- data.frame(name, age, likes_liquorice)
```

This is the result we get when calling `df`:

```
##      name age likes_liquorice
## 1   Sarah  45           FALSE
## 2   Paula  23           FALSE
## 3 Antonia  31           FALSE
## 4   Peter  27            TRUE
```

Next, we apply a few functions to `df` that often come in handy when working with data frames:

```
colnames(df)   # Show the data frame's columns names
ncol(df)       # Show the number of columns
rownames(df)   # Show the data frame's row names
nrow(df)       # Show the number of rows
```

```
## [1] "name" "age" "likes_liquorice"
## [1] 3
## [1] "1" "2" "3" "4"
## [1] 4
```

The last object type we will cover here are **lists**. Lists are the most flexible data structure in R. They allow to collect any kind of object in one "overarching" object (e.g. data frames, vectors, arrays, matrices, scalars, ...).

Say that, looking back to the example data frame above, we also want to add a short description of the data we just created. We can do this by combining the `df` object above with a simple character vector within a list (which we also call `list` here):

```
name <- "liquorice data"
list <- list(data = df, title = name)
```

2.6 | Operators

Now that we discussed the two basic "ingredients" of R, functions and objects, we will now devote some time to some basic **operators** that are needed on a regular basis.

Since R is a programming language developed for statistical computations, all basic arithmetic operations are readily available "out of the box". In other words, R can also be used as a simple pocket calculator:

```
# Using a semi-colon, we can write several lines of code in one:
5+8; 6^3; 6*(10/3)
```

```
## [1] 13     [1] 216     [1] 20
```

Furthermore, we have already used operators in previous sections: for example, the assignment operator (<-, ->, or =) we used to define new objects; or the *"pull"* operator ($) that we employed to "extract" a specific variable from our data frame `df`.

There is also a third type of operator that can be used to evaluate "logical" statements. This includes so-called *relational operators* such as > (greater than), >= (greater or equal), < (smaller), <= (smaller or equal), != (not equal), and == (equal); as well as *Boolean operators*: & (and), | (or), and ! (not). These relational and Boolean operators are helpful, especially when combined with each other, to determine if certain elements within a vector, data frame or list fulfill a certain condition or not. Time to illustrate this with a few examples.

```
# Check if "Capitalized" is equal to "capitalized"
# Note the upper vs. lowercase spelling
"Capitalized" == "capitalized"
```

```
## [1] FALSE
```

```
# Define two objects (x,y); then test if two conditions are fulfilled:
x <- 20; y <- 9
x > 10 & y != 10
```

```
## [1] TRUE
```

Let us also explore what happens if we apply operators to vectors with multiple elements. Below we check which elements in the age variable in our data set `data` have a value above 45:

```
data$age > 45
```

```
## [1]  TRUE FALSE FALSE  TRUE  TRUE  TRUE  TRUE ...
```

A last, and somewhat special operator we cover here is the *pipe*, which is written like this: %>%. This operator is largely R-specific and has no direct equivalent in other programming languages. Furthermore, the pipe is the only operator that is not part of Base R; it has been introduced by the tidyverse, and therefore one of its packages has to be loaded for it to be available[1]. Pipes have now become one of the most widely used tools in R because they come with two big advantages:

1. Functions can be applied to an object without having to name the object again in the function.
2. Pipes can be used to **chain** several functions **together**.

Let us use an example to illustrate how helpful the pipe operator can be at times. Say that I want to extract the age variables from my data frame `data`, calculate the mean of the variable, and then log-transform that value. Using pipes, this is possible in just one line of code:

```
data %>% pull(age) %>% mean() %>% log()
```

[1] Since R version 4.1.0, a pipe operator is also available in Base R (meaning that no additional packages need to be loaded to use it). This Base R pipe works similarly to the tidyverse version that we will cover in this tutorial but uses the |> symbol instead. A detailed comparison of the tidyverse and Base R pipe is provided in this article.

```
## [1] 3.813315
```

The `pull` Function

The `pull` function is the equivalent of the `$` operator within pipes. The function function "pulls" a variable from the data set and passes it to the next function.

2.7 | Slicing & Indexing

The last part of our introduction to R involves data slicing. RCT analysis data is almost always stored in data frames; therefore, it is helpful to learn how we can extract and reshape chunks of our dataset to perform further operations on them.

There are various ways to extract data from a larger data frame. Some options, like the `$` operator or the `pull` function to extract single columns we already discussed before. Another, and more general method to extract slices of data from a data frame is to use **square brackets** `[,]`.

The square bracket is added to the name of the data frame, and typically has two components separated by a comma: the first is used to indicate the *rows*, while the second determines the column from which a value should be extracted. This type of indexing follows the mathematical notation of matrices:

$$\texttt{A[2,1]} = \mathbf{A}_{2,1} = \overbrace{\begin{bmatrix} a_{11} & a_{12} & \cdots & a_{1n} \\ a_{21} & a_{22} & \cdots & a_{2n} \\ \vdots & \vdots & \ddots & \vdots \\ a_{m1} & a_{m2} & \cdots & a_{mn} \end{bmatrix}}^{\text{variables}}$$

Thus, the general form of slicing data frames using the square bracket operator is `data[row, column]`. To use the operator, we have to provide it with some form of index; typically, the number of the row and/or column as it appears in the data frame is used to do this. For example, if we want to extract the element of the third row in the seventh column of our data set `data`, we can use the following code:

```
data[4,7]
```

```
## [1] 19
```

Using the concatenate function `c`, we can also extract a slice spanning multiple rows and columns. If adjacent rows or columns should be extracted, we can alternatively use a colon (`:`); e.g., if columns 5 to 10 should be extracted, we could write the index as `5:10`. Putting these pieces together, imagine that we want to see the first three entries of the 6th and 16th column. Using bracket notation, we can achieve this like so:

```
data[1:3, c(16, 6)]
```

```
##   cesd.0 cesd.2
## 1     24     30
## 2     38     NA
## 3     35      0
```

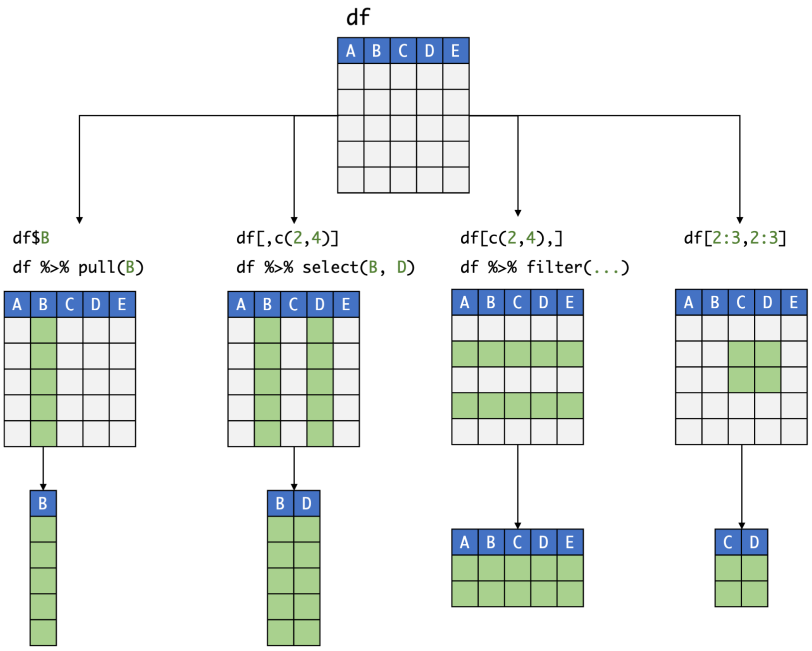

Some ways to subset a data frame, with the corresponding R *code.*

If one of the slots in the bracket (either the left or the right-hand side) is left empty, the entire row or column, respectively, is extracted:

```
data[,21]
```

```
## [1] 0 0 1 0 0 1 1 1 0 1 0 0 0 1 0 1 0 1 1 1 1 0 1 1 1 1 0 0 [...]
```

Instead of numbers, we can also use the name of a column as the index. Please note that, since rows typically do not have a name defined in the data frame, this is usually only possible for rows. To illustrate this, let us extract the first-row entry of the variable age:

```
data[1,"age"]
```

```
## [1] 57
```

Previously, we learned that relational operators can be used to determine if some condition (e.g. `age > 40`) is `TRUE` or `FALSE`. These resulting logical values can also be used as an index in the square brackets. Combining square brackets with relationship operators is particularly helpful because this allows filtering out data based on some pre-defined criteria. For example, if we want to extract the CES-D values of all individuals older than 40, we can use the following code:

```
data[data$age > 40,"cesd.0"]
```

```
## [1] 24 35 36 28 34 42 38 27 37 27 25 20 37 21 17 26 28 32 [...]
```

Slicing is also possible using the `filter` and `select` functions, which are part of the tidyverse. A special feature of these functions is that they are very "pipe-friendly", which leads to less convoluted

code when complex filters are applied. As an example, let us filter out all patients in `data` who are male (i.e. the variable `sex` equals `0`) and older than `40`, then select their CES-D values at all three assessment points, and then inspect the first three rows of the extracted data. That is a quite complex operation, but using a pipe makes it considerably easier to implement it:

```
data %>%
  filter(age > 40, sex == 0) %>%
  select(cesd.0, cesd.1, cesd.2) %>%
  head(3)
```

```
##   cesd.0 cesd.1 cesd.2
## 1     37     13      5
## 2     27     31     38
## 3     25     33      6
```

This concludes our brief journey into the R universe. Naturally, this brief overview is not enough to become a proficient R user, but the basics we covered here are a solid basis to proceed to the next chapter, in which we will begin with our hands-on analysis of RCT data. Before starting with the next chapter right away, it might be helpful to practice what we have learned in this part of the tutorial a little further. In the "Test Your Knowledge" box below, we have compiled a few coding exercises, with solutions provided at the end of the tutorial.

!

***Don't Panic*: A Note on Error Messages**

Especially for beginners, error messages and how to fix them tends to be one of the most excruciating parts of learning R. In GUI-based software, getting an error message usually means that something *really, really bad* has happened, so it is understandable that people tend to get nervous once the first red error messages start popping up. Thus, it is important to remember that everyone, even expert R programmers, get error messages *all the time*.

Deciphering and fixing error messages becomes easier with time, but even so, we have at our disposal one of the best troubleshooting tools thinkable: *Google*. If you get an error message that you find confusing, do not hesitate to copy and paste it into Google and search for results. There are countless programming forums on the Internet, and chances are high that someone was confronted with the same problem before. If your error message is not in English, it helps to set your locale to English first (using `sys.setenv(LANG = "en")`, because most programming content on the Internet is written in that language. If you run the code again, all messages should appear in English, and you are ready and set to search for help for them on the Internet.

Lastly, also make sure that your message is, in fact, an *error* message. R distinguishes between simple `Messages`, `Warnings`, and `Errors`. Only a message preceded by `Error` means that there was a fatal problem and that the code could not be run. Warnings alert us that something (may) have not worked as intended, but that the function could still be run. Messages work more like notes and tell us that some kind of operation or action has been performed for us by the function.

Test Your Knowledge

To complete the following exercises, our example RCT data set must be imported under the name `data` (see section 2.3):

1. Log-transform the variable age in `data` and save the result as `age.log`.
2. Square all values in `cesd.1` and save the result as `cesd.1.squared` *within* the data set `data`.
3. Calculate the mean and standard deviation (SD) of the variable `cesd.2`. If necessary, use the Internet to find out which function in R calculates the standard deviation of a numeric vector.
4. Save the calculated mean and standard deviation of `cesd.2` in a list element.
5. Does the variable `atsphs.0` in `data` have the desired class `numeric`? Try to confirm this using R code.
6. Create two new variables in `data`: (1) `age.65plus`, a `logical` variable which indicates if a person's age is 65 or above; and (2) `cesd.diff`, a variable that contains the difference between `cesd.0` and `cesd.1` for each patient.
7. Using the pipe operator, filter out the records of all patients who are male (`sex=0`) and part of the intervention group (`group=1`); then, in this subset, select the variable `age` and calculate its mean.
8. In the fifth and sixth row of `data`, change the value of `degree` to `NA` (missing).

The solutions for these exercises are provided in the appendix.

■

PART 3 | Descriptive Analysis

We start our evaluation with a descriptive analysis of our trial data. The data we will be working with for the remainder of this tutorial can be downloaded **here**, or from the tutorial's Github repository (MathiasHarrer/rct-tutorial). We refer readers who have skipped the first part of the tutorial to Chapter 2, where we covered how to correctly set up one's R environment and import the data used in the following analyses.

First, let us familiarize ourselves with the data. Our dataset is based on a "real-world" RCT which evaluated the effectiveness of an Internet-based treatment for subthreshold depression to a waitlist control condition (Ebert et al., 2018). Notably, the data set provided here does not consist of the original individual patient data collected in the trial; we have simulated a "clone" of this trial that shares most of its statistical properties but does not contain any actual patient data. The simulation code is provided in the Github repository (see here).

Our primary outcome in this trial evaluation will be patients' depressive symptom severity at post-test (7 weeks after randomization), as measured by the *Center for Epidemiological Studies' Depression Scale* or **CES-D** (Radloff, 1977). The scores obtained by this instrument can take values from 0 to 60, with higher values indicating higher depressive symptom severity. The CES-D was also used to measure depressive symptoms severity at baseline and at a 3-month follow-up. This means that our data set contains three variables: `cesd.0`, `cesd.1` and `cesd.2`, which store the CES-D values at baseline, post-test and 3-month follow-up, respectively. The other recorded variables are:

- **Behavioral activation** (`badssf`), as measured by the *Behavioral Activation for Depression Scale*, BADS-SF (Manos et al., 2011). This outcome was measured at baseline, post-test, and 3-month follow-up.
- **Anxiety** (`hadsa`), as measured by the anxiety subscale of the *Hospital Anxiety and Depression Scale*, HADS-A (Zigmond and Snaith, 1983). This outcome was measured at baseline, post-test, and 3-month follow-up.

- **Age** (age), measured in years.
- **Sex** (sex), which is coded as 0 = male, 1 = female.
- **Previous psychotherapy experience** (prevpsychoth), which can be 1 (yes) or 0 (no).
- **Previous health training experience** (prevtraining), which can be 1 (yes) or 0 (no).
- **Children** (child), which can be 1 (yes) or 0 (no).
- **Employment** (employ), which can be 1 (yes) or 0 (no).
- **Relationship** (rel), which can be 0 (single), 1 (married/in a relationship), 2 (divorced/separated), or 3 (widowed).
- **Education** (degree), which is encoded as 0 (no formal education completed), 1 (education up to high school only; 7-9 years), 2 (high school education; 12-13 years), 3 (education after highschool), 4 (post-graduate education; > 17 years), 5 (Other).
- **Attitudes toward seeking professional help** (atsphs.0), as measured by the ATSPPH-SF (Picco et al., 2016), whereby higher value indicate a higher willingness to seek professional psychological help.
- **Credibility and expectancy** (ceq), as measured by the *Credibility/Expectancy Questionnnaire* (Devilly and Borkovec, 2000).
- **Trial condition** (group), where 1 indicates the treatment group (Internet-based intervention for subthreshold depression), and 0 the control group (waitlist).

An excellent method to get an overview of all variables included in a data frame is to use the skim function included in the skimr package (Waring et al., 2022). Once this package has been installed on our computer, we can run the following code:

```
library(skimr)
skim(data)
```

```
## -- Data Summary ------------------------
## Values
## Name                       data
## Number of rows             546
## Number of columns          21
## [...]
##
## -- Variable type: character ---------------------------------
##   skim_variable n_missing complete_rate min max empty n_unique
## 1 id                    0             1   4   6     0      546
## 2 sex                   0             1   1   1     0        2
## 3 prevpsychoth          0             1   1   1     0        2
## 4 prevtraining          0             1   1   1     0        2
## 5 child                 0             1   1   1     0        2
## 6 employ                0             1   1   1     0        2
## 7 degree                0             1   1   1     0        4
##
## -- Variable type: numeric ------------------------------------
##    skim_variable n_missing complete_rate  mean     sd
## 1  badssf.2            165         0.698 27.0   8.00
## 2  badssf.1            155         0.716 27.9   6.96
## 3  hadsa.2             165         0.698  7.78  3.93
## 4  hadsa.1             155         0.716  7.95  3.47
## 5  cesd.2              165         0.698 20.1  10.3
## 6  cesd.1              155         0.716 19.7   9.15
## 7  age                   0         1     45.3  11.5
```

```
## 8  rel                0          1      0.855  0.695
## 9  cesd.0             0          1     26.6    7.13
## 10 atsphs.0           0          1     13.9    2.57
## 11 ceq                0          1     34.7    8.44
## 12 badssf.0           0          1     26.3    6.02
## 13 hadsa.0            0          1     10.0    3.04
## 14 group              0          1      0.5    0.500
```

There is a lot to unpack here, so let us go through the output (which we simplified a little here) step by step. First, we presented with a summary of our data set: we have 546 rows, corresponding to the N=546 patients included in our trial. We also see that we have 21 variables in our data, details of which are provided below. The `skim` function splits the displayed summary statistics by `class`; the first section shows columns encoded as `character` variables, and the second section shows the `numeric` columns.

This is also a good way to check if imported variables have indeed the desired class, which is not the case in our example: the only "real" character variable in our data set is `id`; all other columns are actually numeric (although one could also encode them as factors). Thus, let us convert those variables first, which can be done using the `as.numeric` function:

```
data$sex <- as.numeric(data$sex)
data$prevpsychoth <- as.numeric(data$prevpsychoth)
data$prevtraining <- as.numeric(data$prevtraining)
data$child <- as.numeric(data$child)
data$employ <- as.numeric(data$employ)
data$degree <- as.numeric(data$degree)
```

To further eyeball the data, it is also helpful to run the `skim` function separately for the intervention and control group. We can achieve this by using the `filter` function within a pipe:

```
data %>% filter(group == 0) %>% skim() # Control group
data %>% filter(group == 1) %>% skim() # Intervention group
```

Splitting up the results by group is particularly helpful to see differences in the primary outcome. For now, let us focus on the values of `cesd.1` and `cesd.2`, which measure the depressive symptom severity at post-test and 3-month follow-up, respectively:

```
##-- Variable type: numeric ---------------------------
##   skim_variable n_missing complete_rate  mean    sd
## 5 cesd.2               81         0.703 22.5  10.4
## 6 cesd.1               76         0.722 23.1   8.51
## [...]
## 5 cesd.2               84         0.692 17.7   9.66
## 6 cesd.1               79         0.711 16.4   8.54
```

The upper rows show the output for the control group and the lower ones for the intervention group. First, we see that, depending on the group and outcome, data are available for 69.2-72.2% of all patients. That means that roughly one-third of all data at post-test and follow-up is missing, which is considerable. We see that the `complete_rate` is somewhat higher at post-test and approximately similar in both groups. More importantly, we see that, at both assessment points, the mean CES-D value in the intervention group is lower than in the control group. This is a preliminary sign that the intervention could have had an effect on the recruited sample, but of course, a more rigorous test is required to confirm this.

It is also helpful to have a look at the distribution of our primary outcome at baseline, post-test and 3-month follow-up. To create a histogram of all three variables, we can use the `multi.hist` function included in the psych package (Revelle, 2022):

```
library(psych)
multi.hist(data %>% select(cesd.0, cesd.1, cesd.2), ncol = 3)
```

There are a few interesting things to see here. First, compared to baseline, the CES-D scores at post-test and follow-up are shifted slightly to the left. This could be caused by an inherent effect of the intervention in the treatment group; but other causes such as regression to the mean or spontaneous remission could also play a role. We also see that, while all three histograms roughly follow a bell curve, the post-test and follow-up values seem to be more dispersed, with more values on the lower end of the scale, but also some very high scores on the other end.

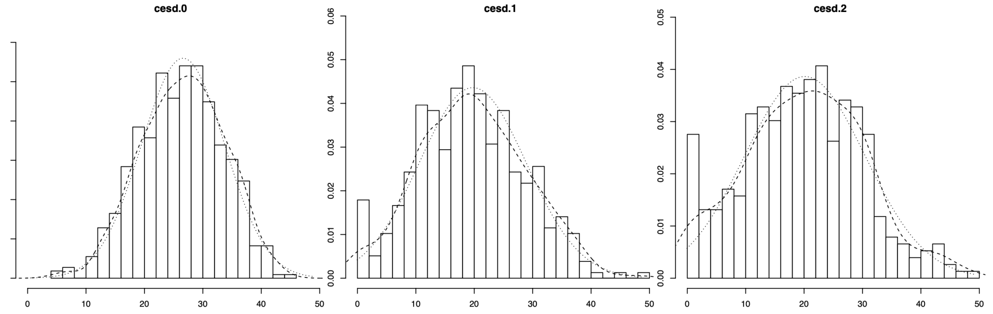

The last step in our descriptive analysis is to quantify to **amount of missing data** at all assessment points. This part is crucial since loss-to-follow-up is also a mandatory part of the Consolidated Standards of Reporting Trials (CONSORT; Moher et al., 2012) flow chart, which we will be creating later.

To create an overview of the missing outcome data, we focus on our primary outcome, the `cesd` variables. To count the missings, we can use the very helpful `is.na` function. This function checks if elements in a vector are missing (`NA`). It returns `TRUE` if that is the case, and `FALSE` otherwise. We can then apply the `sum` function to this result to count the number of missings in a vector. Here is a small example:

```
x <- c(1, 2, 3, 4, 5, NA, NA, 200)      # Create vector
sum(is.na(x))                           # Count the number of missings
```

```
## [1] 2
```

We will now put this function into practice and extract the number of missings in the `cesd` variables at all three assessment points. To simplify this, we use the `with` function, which tells R that all code written within the function should be run with our data set `data`. This means that we do not have to extract each variable from `data` using the `$` operator. We save the result as `na.all`. Then, we divide the results by the number of rows in `data` to get the amount of missing data in percent, and save this result as `na.all.p`.

```
# All trial data
with(data, {
  c(sum(is.na(cesd.0)),
    sum(is.na(cesd.1)),
    sum(is.na(cesd.2)))
}) -> na.all

# Calculate percentages
na.all.p <- na.all/nrow(data)
na.all.p
```

```
## [1] 0.0000000 0.2838828 0.3021978
```

We are not only interested in the *overall* dropout but also in the individual missingness in each randomized group. Therefore, let us repeat the procedure from before for the intervention and control group specifically.

```
# Do the same for intervention group only...
data %>%
  filter(group == 1) %>%
  with({
    c(sum(is.na(cesd.0)),
      sum(is.na(cesd.1)),
      sum(is.na(cesd.2)))
  }) -> na.ig

na.ig.p <- na.ig/nrow(data %>% filter(group == 1))

# ... and control group only
data %>%
  filter(group == 0) %>%
  with({
    c(sum(is.na(cesd.0)),
      sum(is.na(cesd.1)),
      sum(is.na(cesd.2)))
  }) -> na.cg

na.cg.p <- na.cg/nrow(data %>% filter(group == 0))
```

Next, we compile all calculated values in one data frame:

```
# Collect all data in data frame
na <- data.frame(na.all, na.all.p = na.all.p*100,
                 na.ig, na.ig.p = na.ig.p*100,
                 na.cg, na.cg.p = na.cg.p*100)

# Give the rows fitting names
rownames(na) = c("t0", "t1", "t2")
round(na, 2)
```

```
##    na.all na.all.p na.ig na.ig.p na.cg na.cg.p
## t0      0     0.00     0    0.00     0    0.00
## t1    155    28.39    79   28.94    76   27.84
## t2    165    30.22    84   30.77    81   29.67
```

We can now use this information to populate the study flow chart. Such flow charts are mandated by the CONSORT reporting guidelines that most biomedical journals have endorsed, and which should be seen as a standard part of every RCT analysis report. It is possible to generate CONSORT flow charts directly in R using the `consort` package (Dayim, 2023). However, especially for beginners, it may be more convenient to simply download the official PDF or MS Word template from the CONSORT website, and then populate it with the numbers that we just calculated.

In the graphic on the next page, we have used the missingness data we calculated above to indicate the number of participants providing data, as well as the number of participants being lost to follow-up at each assessment point. Please note that the CONSORT flow chart also contains information on the screening process (how many individuals were screened, how many were excluded from the trial, and for what reason?). This information is not included in the final RCT data set and is something that must be recorded while the trial is running.

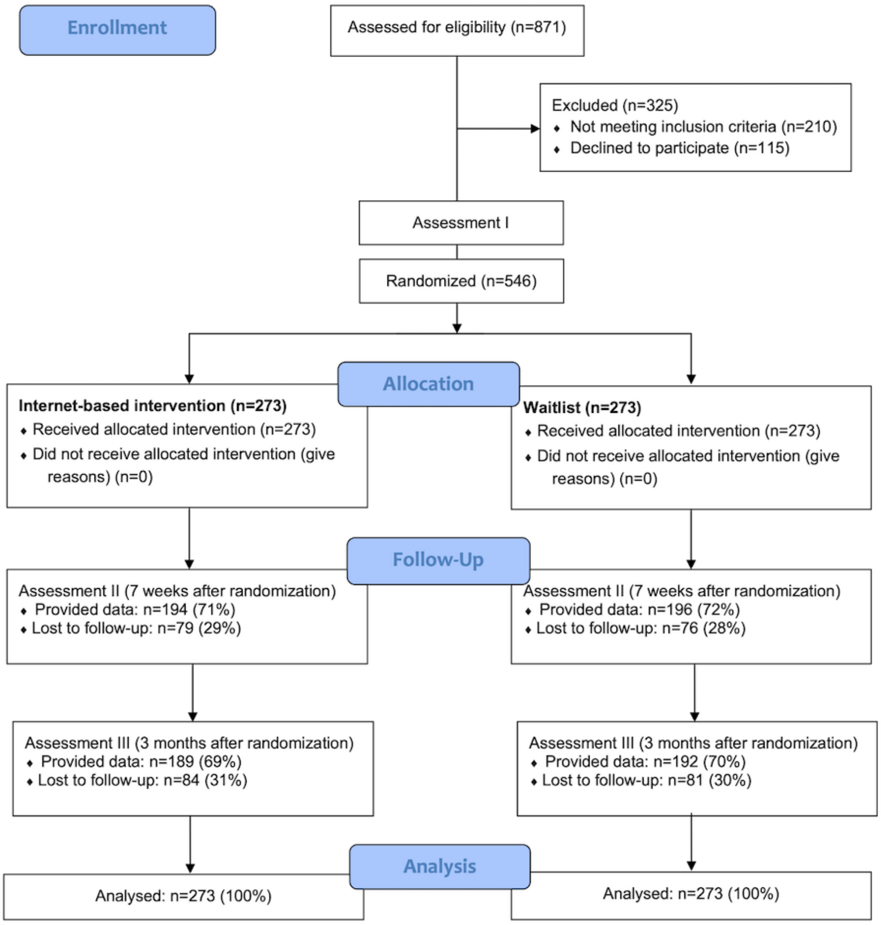

Exemplary CONSORT flow chart for our trial.

The CONSORT Statement has been first introduced in 1996 to improve the reporting of randomized clinical trials in health research. Its goal is to provide a set of standards that all RCT reports should adhere to. The CONSORT guidelines are supported by almost all relevant journals in the mental health field, so adhering to them is highly recommended.

The latest 2010 version of the CONSORT statement consists of a main paper (Schulz et al., 2010) and an elaboration and explanation article (Moher et al., 2012), both of which are extremely helpful resources for novice RCT analysts. The core parts of the CONSORT Statement are the trial flow chart, which we now learned how to create, and the 25-item *CONSORT Checklist*, which should be filled out and provided with every RCT report, and which can be downloaded here.

PART 4 | Missing Data

Having completed our descriptive analysis, we have now had the chance to familiarize ourselves with the structure of our data, as well as some of the patterns we find in it. Most importantly, we have found out that roughly 30% of all data at the post and follow-up assessment are missing. Missingness

of this magnitude is not uncommon in "real-world" evaluations of psychological interventions, but this does not imply that we should take it lightly. In the primer article, we described that missing data poses a real risk to the validity of RCTs. We do not know for sure which mechanism created the missing data; but if the data are not *missing completely at random* and we do not account for that, it is very much possible that our estimates will be biased.

In the next section, we will therefore take some time to discuss how missing data mechanisms are typically classified in the literature, and what implications this has on our analysis. You might be wondering why we are making such a fuss about missing data. The reason is that some degree of missing data is almost unavoidable, even in relatively controlled designs such as RCTs; and that the way missing data is currently handled in (mental) health trials still leaves much room for improvement (see Table 1 in the primer paper for an overview). Unfortunately, R does not provide adequate missing handling "out-of-the-box" either. Let us illustrate that with a little example. Below, we fit a simple linear model using the `lm` function. Our dependent variable `y` is regressed on `x`, but `x` has a few missings:

```
# Define some example data; x has three missings
y <- 1:10
x <- c(1, NA, NA, NA, 3, 5, 8, 10, -1, 10)
summary(lm(y ~ x))
```

```
## [...]
##             Estimate Std. Error t value Pr(>|t|)
## (Intercept)   4.9403     1.7738   2.785   0.0387 *
## x             0.3172     0.2709   1.171   0.2945
## ---
## Signif. codes: 0 ‘***’ 0.001 ‘**’ 0.01 ‘*’ 0.05 ‘.’ 0.1 ‘ ’ 1
##
## Residual standard error: 2.904 on 5 degrees of freedom
## (3 observations deleted due to missingness)
```

The most important information can be found in the last line, in which R tells us that the three observations were discarded before the model was fitted, a strategy that is also known as *listwise deletion*. This is a common *ad hoc* solution to deal with missing data in practice and is still often found in mental health research. However, it is not a recommended way to deal with loss-to-follow-up in randomized trials.

4.1 | Missing Data Mechanisms

The way statisticians think about missing data has been shaped in large measure by the seminal work of Donald Rubin (Rubin, 1976; van Buuren, 2018, chap. 2.4.4). The core tenet of Rubin's framework is that the presence and absence of observed data is the result of a probabilistic process. Our goal as analysts is to approximate this process using an adequate *missing data model*.

According to Rubin, these missing data models fall into three "prototypes", which make different assumptions as to why some values were observed, while others "went missing". We already mentioned these subtypes in the primer article, where we learned that data can either be *missing completely at random* (MCAR), *missing at random* (MAR) or *missing not at random* (MNAR). We will now take some time to discuss these types of missingness in greater detail, and what implications they have when analyzing RCT data.

Before doing so, let us introduce some common notation. We are aware that equations such as the ones we are about to discuss can seem frightening at first, but they are actually an excellent way to describe precisely the idea behind these different missingness mechanisms, so bear with us. First, let us list the "ingredients" we need to define each mechanism:

- $\mathbf{Y}$: The variables in our data set, which contain partially missing data: $\mathbf{Y} = (\mathbf{Y}_{\text{obs}}, \mathbf{Y}_{\text{mis}})$.
- $\mathbf{Y}_{\text{obs}}$: All the values in $\mathbf{Y}$ which were actually observed (i.e., are not `NA`).

- $\mathbf{Y}_{\text{mis}}$: The values in $\mathbf{Y}$ which were *not* observed, but exist *in theory*.
- $\mathbf{R}$: A matrix of the same size as $\mathbf{Y}$, but which only consists of 0's (value missing) und 1's (value observed). This is also known as the "response indicator".
- ψ: Parameters of the missing data model (these parameters are typically not relevant to the scientific question itself).

Using these parameters, we can assemble a general formula that builds the basis of all missingness mechanisms:

$$P(\mathbf{R} = 0|\mathbf{Y}_{\text{obs}}, \mathbf{Y}_{\text{mis}}, \psi). \tag{1}$$

As indicated by the P up front, this is a *conditional probability*, where the | part can be read as "given". Taken together, the formula tells us that every missingness model should give us the probability of some value not being recorded ($\mathbf{R} = 0$), *given* the observed data $\mathbf{Y}_{\text{obs}}$, the missing values $\mathbf{Y}_{\text{mis}}$, and some missing data model parameters ψ (which we can ignore for now).

This formula is quite abstract, but there is something important to draw from it. It tells us that all missingness mechanisms make some statements about how $\mathbf{R}$, $\mathbf{Y}_{\text{obs}}$ and $\mathbf{Y}_{\text{mis}}$ relate to each other. As we will illustrate in the next sections, the main difference between the mechanisms is the degree to which they allow the formula above to be "simplified" by removing $\mathbf{Y}_{\text{obs}}$, $\mathbf{Y}_{\text{mis}}$, or both $\mathbf{Y}_{\text{obs}}$ *and* $\mathbf{Y}_{\text{mis}}$ from the equation.

4.1.1 | Missing Completely at Random (MCAR)

If the data are missing completely at random (MCAR), we assume that the missingness of values (as encoded by $\mathbf{R}$) is completely independent of $\mathbf{Y}$. As it says in the name, we believe that missings occurred in a fully random fashion; they are neither associated with any of the observed data $\mathbf{Y}_{\text{obs}}$, nor with the unobserved values $\mathbf{Y}_{\text{mis}}$. Equation 1 from before can therefore be simplified like this:

$$P(\mathbf{R} = 0|\mathbf{Y}_{\text{obs}}, \mathbf{Y}_{\text{mis}}, \psi) \Rightarrow P(\mathbf{R} = 0|\psi), \tag{2}$$

which tells us that the response indicator only depends on ψ, which can be interpreted as the overall missingness probability (i.e., there generally tend to be many or rather few missings in the data). It is quite clear that this is a very strong assumption, which may not be very plausible in practice.

4.1.2 | Missing at Random (MAR)

The missing at random (MAR) assumption presumes that the missingness of values denoted by $\mathbf{R}$ does depend on some information in $\mathbf{Y}$, but in a special way. We assume that $\mathbf{R}$ only depends on the observed information $\mathbf{Y}_{\text{obs}}$, so that $\mathbf{Y}_{\text{mis}}$ drops out of the equation:

$$P(\mathbf{R} = 0|\mathbf{Y}_{\text{obs}}, \mathbf{Y}_{\text{mis}}, \psi) \Rightarrow P(\mathbf{R} = 0|\mathbf{Y}_{\text{obs}}, \psi). \tag{3}$$

Put differently, the MAR assumption states that, by using all the information in the observed data, we can accurately describe why some values are missing. After controlling for these factors, the data are missing completely at random again. We also assume that the missing values $\mathbf{Y}_{\text{mis}}$ themselves carry no special information beyond what is already captured by $\mathbf{Y}_{\text{obs}}$, which is why they can be removed from the equation.

4.1.3 | Missing Not at Random (MNAR)

The missing not at random (MNAR) assumption is the most general of the three "prototypes". When the data are MNAR, we assume that the missingness indicator $\mathbf{R}$ depends on both $\mathbf{Y}_{\text{obs}}$ *and* $\mathbf{Y}_{\text{mis}}$. If this is the case, there is no way to further simplify equation 1:

$$P(\mathbf{R}=0|\mathbf{Y}_{\text{obs}},\mathbf{Y}_{\text{mis}},\psi) \Rightarrow \ ? \quad (4)$$

The main implication of the MNAR assumption is that the missingness of our data as encoded by $\mathbf{R}$ does, at least to some extent, depend on the values of the missing data $\mathbf{Y}_{\text{mis}}$ *themselves*. As analysts, this brings us into somewhat of a conundrum: it means that, if our missing data model should accurately reflect the reality behind our data, we have to account for the information included in $\mathbf{Y}_{\text{mis}}$; but this is impossible because the information in $\mathbf{Y}_{\text{mis}}$ has never been recorded. The fact that these values are missing is the exact reason why we need a missing data model in the first place.

This may sound paradoxical, but there are many scenarios, including RCT evaluations, where the MNAR assumption is very much plausible. Let us assume, as we did in the primer article, that patients did not provide outcome data in the intervention group because they did not experience any benefits of the intervention. In this case, it is likely that our measurement of the outcome (say, depressive symptom severity) is missing precisely *because* of its value. Some patients still experience very high levels of depression, and that is why their depression score was not recorded. We can then say that the data in our trial are MNAR.

4.1.4 | Implications

What implications do these different missing data mechanisms have on our analysis? If our data are really MCAR, the answer is straightforward: there will be no systematic bias in our estimates; we only lose statistical power (i.e., we have to live with broader confidence intervals) because some of the data is missing.

Things get more complicated if the data are MAR. Here we assume that values are missing at random *conditionally* on other variables, meaning that our estimates will be biased if we look at some variable in isolation. Imagine that Y is the primary outcome of our trial (like the CES-D score at post-test in our example data). If the data are MAR, summary statistics calculated from Y (e.g. the mean in the treatment and control group) will be biased. However, if we adjust for the information included in other observed variables, this bias can be avoided. One (of several) ways to achieve this is by constructing an imputation model that makes use of this information in our data, as we will do in section 4.2.

From an analytic perspective, MNAR data arguably poses the greatest challenge. If the data are truly MNAR, our estimates are likely biased, and there is no information *within* our data to "rescue" our analysis. We can only hypothesize how and how strongly the underlying missing data model plausibly departs from the MAR assumptions, and implement statistical approaches that represent these "best guesses". In an individual study, there is no way to *prove* that our chosen approach to handling MNAR data is adequate; just as it is also not possible to *prove* that our data are MNAR in the first place. We quote from Molenberghs et al. (2004), p. 447:

> *"MNAR models are [...] typically highly dependent on untestable and often implicit assumptions regarding the distribution of the unobserved measurements given the observed measurements."*

Overall, this underlines the importance of **sensitivity analyses**: by considering the impact of various plausible missing data assumptions on our results, we can make more qualified statements on the robustness of our findings. Methods to handle MNAR data are an open research topic, and several approaches are available. We will cover some of them in section 4.2.3.

One last distinction we want to introduce here is the one between **ignorable** and **non-ignorable missing data**. If data are MCAR or MAR, we can say that our missing data are *ignorable*, while MNAR means that we have *non-ignorable* missing data. Please note that this is a technical term that does not imply that we do not have to "care" about our missings if they are MCAR or MAR. In fact, ignorability means that we can infer from the joint distribution of the *observed* data to one of the *missing* data. When the data are non-ignorable (i.e., MNAR), this assumption is violated:

$$P(\mathbf{Y}|\mathbf{Y}_{\text{obs}},\mathbf{R}=1) \neq P(\mathbf{Y}|\mathbf{Y}_{\text{obs}},\mathbf{R}=0) \quad (5)$$

Thus, if we are dealing with non-ignorable missings, an estimation (imputation) based on the available values is not possible without further ado; assumptions must be made that "go beyond the data".

Which Missing Data Assumption Should I Make?

As mentioned, strictly speaking, there is no way to confirm if the missings in our trial data are ignorable or non-ignorable. Sometimes, differential dropout[a] is taken as a sign of non-ignorable missing data, but that is not necessarily true: differential dropout can lead to biased results but does not have to; conversely, biased results are also possible even when dropout has been equal between groups (Bell et al., 2013). As we mentioned, sensitivity analyses that account for different plausible missing data mechanisms should be seen as a standard part of every RCT evaluation. This makes the question of which mechanism fits the present best less problematic than one may initially fear, as long as several plausible options are covered.

Nevertheless, when designing the statistical analysis plan of a trial, we have to define *a priori* what the "primary" our "main" effectiveness analysis of our trial will entail. This means that we have to choose some mechanism as a plausible starting point, even though we will accompany it with sensitivity analyses later. There are no iron-clad rules, but it has been frequently mentioned that an analysis assuming MAR may often be a good first start. Let us quote a few authoritative sources here:

> *"[W]e recommend that in trials [...], all data should be used in an analysis that makes a plausible assumption about missing data. Usually, this will be a MAR assumption."* (Bell et al., 2014)

> *"The assumption of ignorability is often sensible in practice, and generally provides a natural starting point."* (van Buuren, 2018, , chap. 2.2.6)

> *"When [...] a complete records analysis is insufficient, the framework suggests an analysis assuming MAR. This is because [...] MAR is the most general assumption about the missing data distribution we can make without recourse to bringing in external information."* (Carpenter and Smuk, 2021)

If we assume that the data are MAR, we should also choose a compatible missing data handling method. One option for this is multiple imputation, which we will cover in the next section.

[a] e.g., when there are considerably more missings in the intervention group than in the control group, as is often the case in mental health care research.

A Few Remarks On *Little's MCAR Test*

It is still quite common to see RCT evaluations in mental health research report the results of Little's analysis (1988) χ^2 test, often with the intention to "confirm" that the data are MCAR. This approach is flawed. Our null hypothesis in Little's test is that the missings in our data appeared randomly, i.e., that the data are MCAR. Our ability to reject this null hypothesis depends on the statistical power of the test, which in turn depends on our sample size; this problem is exacerbated if the data are in fact MNAR (Thoemmes and Enders, 2007); if we cannot reject the null hypothesis, this does not confirm that the data are in fact MCAR.

Even if the null hypothesis is rejected, this means that the data can either be MAR or MNAR. The difference between MAR and MNAR is much more important in practice because conventional missing data handling methods (e.g., most multiple imputation approaches) provide valid results under MAR, but not MNAR.

Furthermore, as we learned above, MCAR is not a very plausible missing data assumption in RCTs to begin with, with the MAR assumption typically providing a better starting point.

Taken together, this should underline that the practical utility of Little's MCAR test is very limited, and it is probably best to avoid using it in RCT evaluations altogether. A more comprehensive treatment of the problems with Little's test is provided in Enders (2010, p.21).

4.2 | Multiple Imputation

Multiple Imputation (MI; Rubin, 1996, 2004) is one of the most flexible and commonly used methods for dealing with missing values. The goal of MI is to estimate ("impute") plausible values for missing data, based on the distribution of the observed values.

Importantly, as part of MI, we generate *multiple* imputations for each missing value to reflect the uncertainty of our estimation. The complete data sets generated in this way are then analyzed simultaneously (we could, for example, calculate a sample mean in all imputed data sets). In the end, all results are then pooled into one estimate.

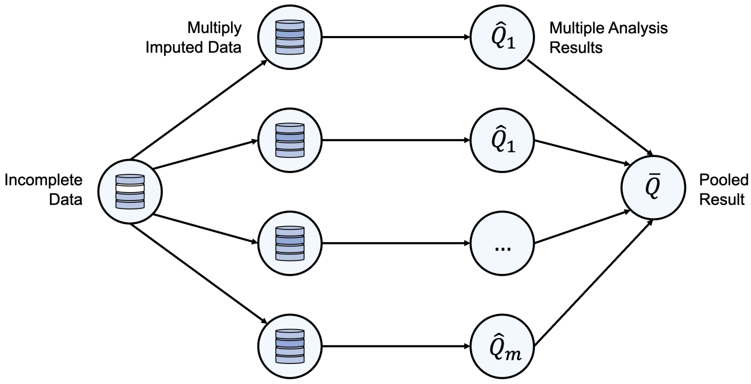

Multiple imputation (adapted from van Buuren and Groothuis-Oudshoorn, 2011a).

MI can be used under the assumption of MCAR and MAR (and under certain conditions also MNAR). When applied correctly, MI results in an unbiased estimation of population parameters (regression weights, population means, correlations, etc.) and their variance ("asymptotically unbiased") despite the presence of missing values (White et al., 2011).

In terms of implementation, two broad "flavors" of MI can be distinguished:

- **Joint Modeling (JM)**: A multivariate distribution ("joint model") for the missing data is specified, and imputations are sampled using Markov Chain Monte Carlo (MCMC; Schafer, 1997).
- **Fully Conditional Specification (FCS)**: Incomplete variables are imputed in a sequential and iterative process, one variable at a time. Unlike JM, it is not necessary to find a multivariate distribution of the missing data for FCS. In this regard, FCS is "atheoretic".

In this tutorial, we will focus on how to implement MI using fully conditional specification (FCS). We do this because FCS is a slightly more flexible method that translates well to new analytic tasks; and because it might slightly more convenient to apply for novice analysts. Joint modeling has other strengths, for example when dealing with clustering or other more complex data structures. Since JM is also more "theory-driven" than FCS, it can also have advantages in achieving what is known as "compatibility" or "congeniality" of the imputation model, a topic that we will cover

in section 4.2.3. An excellent way to implement JM in R is via the jomo package (Quartagno and Carpenter, 2022a). A practical introduction to this package can be found in Quartagno et al. (2019).

4.2.1 | Multivariate Imputation By Chained Equations

> *"The MICE algorithm possesses a touch of magic."*
> (van Buuren and Groothuis-Oudshoorn, 2011a)

The MICE (Multivariate Imputation by Chained Equations) algorithm is one of the most commonly used and best-studied FCS approaches. As it says in the name, MICE is based on the idea of *chained equations*, which is what we discuss now.

Imagine that we have a data set $\mathbf{Y}$ that consists of p variables. We can write: $\mathbf{Y} = Y_1, Y_2, ..., Y_p$. The values of the p variables are partly observed, and partly missing. When building our imputation model, we assume that these variables are created by $\boldsymbol{\theta}$, an unknown vector of population parameters. This multivariate distribution of $\boldsymbol{\theta}$ is what we want to approximate in order to generate accurate imputations.

The MICE algorithm achieves this *implicitly*, by sampling over each incomplete variable Y from its conditional distribution, in the following form (van Buuren and Groothuis-Oudshoorn, 2011a):

$$\begin{aligned} &P(Y_1|\mathbf{Y}_{\setminus 1}, \theta_1) \\ &\vdots \\ &P(Y_p|\mathbf{Y}_{\setminus p}, \theta_p). \end{aligned} \tag{6}$$

Here, the subscript $\setminus p$ indicates that all information in our data $\mathbf{Y}$ is used to sample data for the missing values in some variable Y, *except* the imputed values of Y itself. This is a special feature of the MICE algorithm.

MICE is implemented through a method called *Gibbs sampling*. Based on some starting values, θ is estimated for each variable j, which is then directly used to generate imputed values Y_j^*. These values then form the basis for further sampling. This leads to the following set of "chained" equations for each iteration t:

$$\begin{aligned} \theta_1^{*t} &\sim P(\theta_1|Y_1^{\text{obs}}, Y_2^{t-1}, \dots, Y_p^{t-1}) \\ Y_1^{*t} &\sim P(Y_1|Y_1^{\text{obs}}, Y_2^{t-1}, \dots, Y_p^{t-1}, \theta_1^{*t}) \\ &\vdots \\ \theta_p^{*t} &\sim P(\theta_p|Y_p^{\text{obs}}, Y_1^{t}, \dots, Y_{p-1}^{t}) \\ Y_p^{*t} &\sim P(Y_p|Y_p^{\text{obs}}, Y_1^{t}, \dots, Y_{p-1}^{t}, \theta_p^{*t}). \end{aligned} \tag{7}$$

Typically, this process converges after a certain number of iterations and reaches *stationarity* (i.e., it "stabilizes" around a particular set of values). Since in MICE, the previous imputation Y_j^{t-1} is not directly used in the imputation of Y_j^t, this is achieved relatively quickly (often after 5-10 iterations). This iterative process is performed multiple times in parallel to generate the m imputation sets.

Equation 7 above is complex, and it is not necessary to understand every detail of it. What matters is that the formula reveals something special about the MICE algorithm and FCS in general. While we assume that there is some complex multivariate distribution $\boldsymbol{\theta}$ that generated our data, MICE does not try to approximate this distribution directly through some joint model (like JM), but instead defines a separate model for each variable individually. This makes the MICE algorithm somewhat "atheoretic", but dramatically improves the flexibility of the approach, and makes it applicable to data with all kinds of variable types (continuous, binary, categorical). It has also been shown that MICE/FCS generally performs well across various contexts and data structures (Grund et al., 2018; van Buuren et al., 2006; van Buuren, 2007; Enders et al., 2018; de Silva et al., 2017), despite its atheoretic nature.

Our main task when using MICE in practice is thus to define an apt set of predictors for each imputed variable individually, as well as the method by which imputations should be generated. Table 1 provides an overview of commonly used imputation methods, as well as their implementation within the `mice` package (van Buuren and Groothuis-Oudshoorn, 2011b) which we will also use in this tutorial.

Table 1: Commonly used imputation techniques for MICE, by data type.

**Model/Method**	**Implementation**[a]	**Data Type**
Predictive Mean Matching	`pmm`	various
Bayesian Linear Regression	`norm`	continuous
Unconditional Mean Imputation	`mean`	continuous
Bayesian Logistic Regression	`logreg`	binary
Bayesian Polytomous Regression	`polyreg`	factorial

[a]Name of the implementation within the `mice` package.

One of these methods, *predictive mean matching* (PMM; Morris et al., 2014), deserves a little further attention. PMM is a so-called "hot deck" method, which means that it imputes missings based on the real values recorded for other, but statistically similar individuals in our data. In essence, PMM involves a two-stepped procedure: first, we try to predict the values of a variable with missings using other recorded information in our data set; this allows us to create a *predicted* value for both the individuals that were actually observed, $\hat{y}_i^{(\text{obs})}$, and the predicted values of those whose true values were not observed, $\hat{y}_j^{(\text{mis})}$. Note that we use a hat symbol (ˆ) to indicate that we are using predicted values of y.

In the next step, we gauge these predicted values to identify "donors" for each person with missing values. Donors are individuals *without* missings whose predicted values $\hat{y}_i$ are similar to the one of an individual *with* missing data. Put differently, our goal is to find participants with recorded data on y whose predicted values deviate as minimally as possible from the predicted values of the person for which y has to be imputed:

$$\min_i \left| \hat{y}_i^{(\text{obs})} - \hat{y}_j^{(\text{mis})} \right| \tag{8}$$

In most cases, d=3-10 donor candidates are identified this way, and one is then selected randomly to "donate" its recorded value to the person for whom no value was recorded. In the `mice` package, 5 donor candidates are used by default.

PMM is a broadly applicable and typically robust technique for imputations. One of its perks is that it can be used for various data types, including continuous, binary, and categorical variables. Furthermore, since all values come from observed donors, imputing "impossible" values (e.g., an age of -2) is ruled out *a priori*.

Multiple Imputation using MICE: A Few General Tips

Number of imputation sets. Typically, generating m=20 imputations is already sufficient when the amount of missing data is moderate. However, more imputations may be necessary if the goal is to precisely estimate parameters that are often difficult to estimate (e.g., standard errors or variance components); or when there is a large number of missings (van Buuren, 2018, chap. 2.8). In most practical contexts, the time needed for the imputation will be manageable even when the number of imputations is set to a higher value, so using $m \approx$ 50-100 imputations might generally be a good approach in practice.

Number of iterations. As mentioned above, a key feature of the MICE algorithm is that it converges quite fast, so that 20-30 iterations are often sufficient. A higher number can be chosen when it is unclear if the algorithm has converged. Section 4.2.2 covers how to diagnose convergence issues when using MICE, although some of these problems cannot be solved by simply increasing the number of iterations.

Number of variables. Another key question when building an imputation model is the number of auxiliary variables to be used for imputing missing values. First and foremost, variables should be used as predictors in our imputation model if they are plausibly related to the imputed variable(s) and/or their missingness. Including a wide range of meaningful auxiliary variables makes the MAR assumption more plausible, but also increases the complexity of our imputation model, which can lead to estimation problems. The goal is therefore to find a middle ground when the imputation model fails; i.e., to reduce its complexity without reducing the overall plausibility of the model. To quote White et al. (2011):

> *"In general, one should try to simplify the imputation structure without damaging it; for example, omit variables that seem on exploratory investigation unlikely to be required in 'reasonable' analysis models, but avoid omitting variables that are in the analysis model or variables that clearly contribute towards satisfying the MAR assumption."*

4.2.2 | MICE in Practice

After all this technical input, it is time to implement MICE in practice. To build our imputation model, the `mice` and `miceadds` (Robitzsch and Grund, 2022) packages need to be installed and loaded from the library. The `mice` package is the main workhorse we use to set up and run our model, while the `miceadds` package provides additional helper functions.

```
library(mice)
library(miceadds)
```

First, let us explore the missingness pattern in our data frame object `data`. We do this by plugging it into the `md.pattern` function, which creates the following output:

```
md.pattern(data)
```

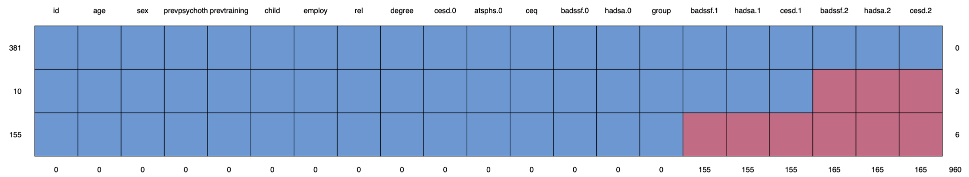

This plot may be difficult to decipher at first, so let us go through it step by step. The rows in the plot above show the distinct missingness patterns, with the numbers to the left indicating how frequently each of them appears in our data. Within each pattern (i.e., row), a blue tile symbolizes that a specific variable (indicated by the columns) has been observed, while a red tile shows that the variable is missing.

This graphic makes it clear that we have three missingness patterns in our data. The first, and most common case (n=381 individuals) is that all variables have been recorded. The second case (n=10 individuals) is that the `badssf.2`, `hadsa.2` and `cesd.2` variables are missing, with the rest being recorded. This pattern coincides with all the patients in our trial who could not be reached at the 3-month follow-up. The last pattern, which is represented by n=155 patients, are individuals whose measurements are missing at both post-test *and* follow-up, but with all the baseline measurements being recorded.

This shows that our data displays what is known as *monotone missingness*: over time, some patients drop out of the trial and cannot be reached anymore, including at later assessment points. This means that the missings gradually increase over time; although, in our case, the number of additional missings at the 3-month follow-up is small. Such a missingness pattern is not uncommon in longitudinal studies in general, and randomized controlled trials in particular.

The next thing we examine is the so-called *influx* and *outflux* pattern of our missings. Using the `flux` function, an influx and outflux coefficient can be calculated for each variable, which can be helpful to determine variables that are more or less relevant for our imputation model. For two variables with the same amount of missingness, a higher influx and/or outflux coefficient means that the variable is more connected with the rest of the data, and might therefore be more relevant for our imputation model.

Calculating the Influx & Outflux Coefficient

The **influx coefficient** I_j of some variable j indicates how well *missing* values in j are covered by *observed* values in other variables; i.e., how much information of other variables can potentially "flow into" j.

Let R_{ij} be the response indicator (1 = observed; 0 = missing) for some person i on some variable j, and let R_{ik} be the response indicator for the same person on some other variable k. The influx coefficient can then be calculated as (van Buuren, 2018, chap. 4.1.3):

$$I_j = \frac{\sum_j^p \sum_k^p \sum_i^n (1 - R_{ij})R_{ik}}{\sum_k^p \sum_i^n R_{ik}} \tag{9}$$

The **outflux coefficient** O_j indicates how well *observed* values in j can be used to cover *missing* values in other variables. For each variable j, we get the following formula:

$$O_j = \frac{\sum_j^p \sum_k^p \sum_i^n R_{ij}(1 - R_{ik})}{\sum_k^p \sum_i^n 1 - R_{ij}}. \tag{10}$$

Let us inspect the results we obtain for our data set `data`. Please note that we add `[,1:3]` to our function call because we only require the first three columns of the output:

```
flux(data)[,1:3]
```

```
##                  pobs    influx outflux
## id          1.0000000 0.0000000 1.00000
## badssf.2    0.6978022 0.2384352 0.00000
## badssf.1    0.7161172 0.2213021 0.03125
## hadsa.2     0.6978022 0.2384352 0.00000
## hadsa.1     0.7161172 0.2213021 0.03125
## cesd.2      0.6978022 0.2384352 0.00000
## cesd.1      0.7161172 0.2213021 0.03125
## age         1.0000000 0.0000000 1.00000
## sex         1.0000000 0.0000000 1.00000
## prevpsychoth 1.0000000 0.0000000 1.00000
## prevtraining 1.0000000 0.0000000 1.00000
## [...]
```

Looking at the output values, we see that many variables in our data have an `influx` coefficient of 0, while their `outflux` is 1, which is the largest possible value. This is because these are baseline variables, which, in our case, have no missings. Naturally, this means that all information is flowing

"out" of these variables, while there is no influx. This could make these variables potentially very valuable as predictors in our imputation model.

We see a different pattern for the variables assessed at post-test and follow-up. Here, the outflux is very low (O_j = 0.03 for post-test variables) or zero altogether (for follow-up measurements). On the other hand, there is some influx for these variables, which is slightly larger for the follow-up variables, because they are covered by both the baseline variables *and* a few post-test measurements.

These results reveal that, since our RCT data displays monotone missingness, the heuristic value of the influx and outflux coefficient is somewhat limited; there is nothing particularly surprising about the results we obtained here. However, when the missingness is not (completely) monotone, the flux indices can indeed be helpful to identify variables that may be more or less helpful to use as predictors in our imputation model.

> **!**
>
> **Limitations of the Flux Coefficients**
>
> The flux coefficients only tell us how well missing values in some variables are connected to observed values in others; they do not assess if a particular variable is actually *predictive* of another. Therefore, we should not rely on them alone to determine which variables to include in our imputation model.

As a last preparation step, we will explore if some of our (completely recorded) baseline variables are associated with the missingness of values at post-test (see e.g. Carpenter and Smuk, 2021). This can be helpful to identify variables that we should consider including as predictors in the imputation model. To do this, we first create a response indicator `R` within our data set, which identifies if our primary outcome, the CES-D score at post-test, has been recorded or not. Then, we split the data by treatment group so that we can examine both separately. We name the resulting two data frames `data.cg` and `data.ig`:

```
data$R <- !is.na(data$cesd.1)
data.cg <- data %>% filter(group == 0)
data.ig <- data %>% filter(group == 1)

# R is not needed for other analyses, so
# we remove it from the original data
data$R = NULL
```

Now, we can run a logistic regression model that predicts response at post-test using our recorded baseline variables. This can be implemented in R using the `glm` function, in which we have to specify `binomial(link = "logit")` so that a logistic regression model is run. In the first argument, we provide the formula, in which we regress `R` on the baseline variables in `data`. Regression models specified in R often take the generic form `y ~ x + z`, which means that `y` is predicted by `x` and `z`.

In the formula argument, we use the `scale` function to center and scale all the continuous variables, which makes it easier to interpret the results. We also plug categorical variables into the `as.factor` function so that they are encoded as factors. This leads to the following code for the intervention group data:

```
glm(R ~ scale(cesd.0) + scale(badssf.0) + scale(hadsa.0) +
      scale(age) + sex + prevpsychoth + prevtraining + child +
      employ + as.factor(rel) + as.factor(degree) +
      scale(atsphs.0) + scale(ceq),
    binomial(link = "logit"), data.ig) %>% summary()
```

Note that, in the end, we use the `summary` function within a pipe to extract the model coefficients. The result is printed below:

```
## [...]
##                       Estimate Std. Error z value Pr(>|z|)
## (Intercept)            2.49061    0.90965   2.738  0.00618 **
## scale(cesd.0)         -0.17242    0.15241  -1.131  0.25791
## scale(badssf.0)       -0.39067    0.16167  -2.417  0.01567 *
## scale(hadsa.0)        -0.35112    0.16134  -2.176  0.02954 *
## scale(age)            -0.24982    0.15666  -1.595  0.11080
## sex                   -0.37733    0.30932  -1.220  0.22251
## prevpsychoth          -1.20921    0.30867  -3.917 8.95e-05 ***
## prevtraining           0.16388    0.38613   0.424  0.67127
## child                 -0.39937    0.31006  -1.288  0.19773
## employ                -0.14342    0.45466  -0.315  0.75243
## as.factor(rel)1       -0.45305    0.34164  -1.326  0.18480
## as.factor(rel)2       -0.47649    0.47114  -1.011  0.31185
## as.factor(rel)3       -2.79634    1.38934  -2.013  0.04415 *
## as.factor(degree)3     0.29925    0.79096   0.378  0.70518
## as.factor(degree)4    -0.28667    0.76578  -0.374  0.70814
## as.factor(degree)5    15.28001  731.88259   0.021  0.98334
## scale(atsphs.0)       -0.29236    0.15425  -1.895  0.05804 .
## scale(ceq)            -0.07254    0.15820  -0.459  0.64656
## ---
## Signif. codes:  0 ‘***’ 0.001 ‘**’ 0.01 ‘*’ 0.05 ‘.’ 0.1 ‘ ’ 1
## [...]
```

As indicated by the asterisks there are several baseline characteristics associated with higher or lower odds that a patient will not be observed at post-test. We see, for example, that higher `badssf.0` and hadsa.0 values make it less likely that patients' post-test score was recorded. The same is true for participants who have previous psychotherapy experience, and this effect is even highly significant (p <0.001). Lastly, we also see that values are more likely to be missing if a patient is widowed (`rel=3`).

There are several takeaways from these results. First, they underline that our data are unlikely to be MCAR; there seem to be systematic factors in the intervention group that predict if a person is more or less likely to drop out from the study. Second, we were able to identify some variables indicative of missingness that we should make sure to include as auxiliary variables in the imputation model.

Lastly, please note that we have mostly focused on the statistical significance of the examined predictors here; naturally, statistical significance also heavily depends on the size of a data set and therefore should not be the only criterion based on which plausibly relevant predictors should be included.

For comparison, let us also examine the results we obtain from the control group:

```
glm(R ~ scale(cesd.0) + scale(badssf.0) + scale(hadsa.0) +
      scale(age) + sex + prevpsychoth + prevtraining + child +
      employ + as.factor(rel) + as.factor(degree) +
      scale(atsphs.0) + scale(ceq),
    binomial(link = "logit"), data.cg) %>% summary()
```

```
## [...]
## Coefficients:
##                       Estimate Std. Error z value Pr(>|z|)
## (Intercept)            3.74120    1.22331   3.058  0.00223 **
## scale(cesd.0)         -0.20275    0.15192  -1.335  0.18201
## scale(badssf.0)       -0.40754    0.15095  -2.700  0.00694 **
```

```
## scale(hadsa.0)      -0.03695   0.14646  -0.252  0.80080
## scale(age)          -0.17722   0.14883  -1.191  0.23375
## sex                 -0.41628   0.31414  -1.325  0.18513
## prevpsychoth        -0.49831   0.29967  -1.663  0.09634 .
## prevtraining        -0.42288   0.38279  -1.105  0.26927
## child               -0.19907   0.29732  -0.670  0.50315
## employ              -0.53185   0.43461  -1.224  0.22105
## as.factor(rel)1     -0.32452   0.34903  -0.930  0.35249
## as.factor(rel)2     -0.47920   0.46621  -1.028  0.30401
## as.factor(rel)3      0.12737   1.29719   0.098  0.92178
## as.factor(degree)3  -1.09337   1.12966  -0.968  0.33310
## as.factor(degree)4  -1.57005   1.11017  -1.414  0.15729
## as.factor(degree)5  12.90396  696.65833   0.019  0.98522
## scale(atsphs.0)     -0.15722   0.15166  -1.037  0.29991
## scale(ceq)          -0.27479   0.15217  -1.806  0.07095 .
## ---
## Signif. codes:  0 ‘***’ 0.001 ‘**’ 0.01 ‘*’ 0.05 ‘.’ 0.1 ‘ ’ 1
## [...]
```

While fewer predictors are significant here, the overall pattern is quite similar. The main exception is the "widowed"-level of the `rel`, the regression coefficient of which has a different sign now, and is not significant anymore.

Based on these results, we can define an **"inlist"**, a selection of baseline variables that should *always* be included in our imputation model. Here, we choose the `badssf.0`, `hadsa.0`, `prevpsychoth` and `rel` variable:

```
inlist <- c("badssf.0", "hadsa.0", "prevpsychoth", "rel")
```

We are now set and ready to initialize our MICE model for the first time. For now, we only provide our data set `data` and specify `maxit = 0` within the `mice` function. This means that a "null imputation" is conducted: the data is checked for potential problems, but no actual imputations are generated. We save the result as `imp0`.

```
imp0 <- mice(data, maxit = 0)
```

```
## Warning message:
## Number of logged events: 1
```

The output tells us that there was one `logged event`. We can use the $ operator to inspect it:

```
imp0$loggedEvents
```

```
##   it im dep     meth out
## 1  0  0     constant  id
```

The function tells us that there is one constant in our data that cannot be used within the imputation model – our patient `id`. Since this variable does not carry any meaningful information anyway, we remove it from our data and save the new data frame as `imp.data`.

```
# Include all variables with logged events ("id" in our case)
# in our "outlist"; then remove those variables from the data frame
outlist <- imp0$loggedEvents$out
imp.data <- data %>% select(-all_of(outlist))
```

Using this new data frame, we can now use the `quickpred` function in `mice` to create a *predictor matrix*. This predictor matrix is the core of our imputation model and indicates which variables will be used as predictors for which variable with missing data. As we learned, MICE as an FCS approach gives us a lot of flexibility in defining this matrix, so it is important to put some thought into this step.

The `quickpred` function takes several relevant arguments that should be specified. First, we have to provide a value of `mincor`, the minimal correlation between a predictor and the imputed variable that is required for that predictor to be used in our imputation model. We set this value to r=0.05, which means that a predictor has to be at least slightly associated with the variable we want to impute; if this is not the case, the variable will be removed from the predictor matrix. The `minpuc` argument in the `quickpred` function follows a similar logic. It lets us define the minimum *proportion of usable cases* within a predictor when imputing another variable with missings. Here, we set this value to p=0.05, which means that at least 5% usable cases are needed and that the variable will be removed as a predictor if this is not the case.

The idea behind both `mincor` and `minpuc` is to *a priori* remove predictors that do not carry a substantial amount of meaningful information in imputing a variable with missings. This is a way to simplify our imputation model so as to avoid computational problems further down the line.

The last argument in `quickpred` we want to specify is `include`. This argument can take a selection of variables that should *always* be used to predict missing values. Previously, based on our inspection of the missingness pattern in both trial groups, we already defined our `inlist` of potentially important predictors, and it makes sense to provide this selection of variables within the function. We save the results of our call to `quickpred` as `pred`.

```
pred <- quickpred(imp.data,
                  mincor = 0.05,
                  minpuc = 0.05,
                  include = inlist)
```

Next, we can inspect the matrix. As we learned before, a great asset of multiple imputation (and FCS in particular) is that it allows to integrate the information captured by auxiliary variables, which can help to make the MAR assumption more plausible. Naturally, this means that, for each missing variable, a sufficient amount of potentially meaningful predictors is used in our imputation model. To check this, we can calculate the *mean number of predictors* for each variable with missings, using the following code:

```
# Mean number of predictors
table(rowSums(pred))[-1] %>%
  {as.numeric(names(.)) %*% . / sum(.)}
```

```
##          [,1]
## [1,] 13.33333
```

The output shows that approximately 13 predictors are used to impute each variable with missings in our data. This is not too bad, given that our data frame `dat.imp` only has 20 variables.

As a next step, we have to slightly adapt the matrix. When imputing data of clinical trials, it is sensible to generate imputations *seperately* for the intervention and control group, because this allows to also capture patterns that might differ between the two groups. Some baseline variables in our data (e.g. depressive symptom severity, age, etc.) could be *effect modifiers*, which means that their influence on the imputed outcome differs between the two groups. Imputing separately for both groups can help to address this issue. However, to implement a group-wise imputation, we first have to make sure our `group` variable is *not* used as a predictor in the matrix. This is because once we start to impute for each group separately, the `group` variable does not carry any information anymore (since it is either all `0` or all `1`).

The remove the `group` variable from our imputation model, we have to set its column in the imputation matrix to zero, which we can do like this:

```
# Set predictor "group" to zero
pred[,"group"] = 0
```

Once these preparations are completed, we can also visualize the imputation matrix. To do this we have to make sure that the `plot.matrix` package is installed and loaded from the library. Then we can use the `plot` function to generate a graph:

```
library(plot.matrix)
plot(pred, main = "Imputation Matrix",
     col = c("grey", "blue"),
     xlab = "predicts",
     ylab = "is predicted",
     adj = 0, las = 2, cex.axis = 0.5)
```

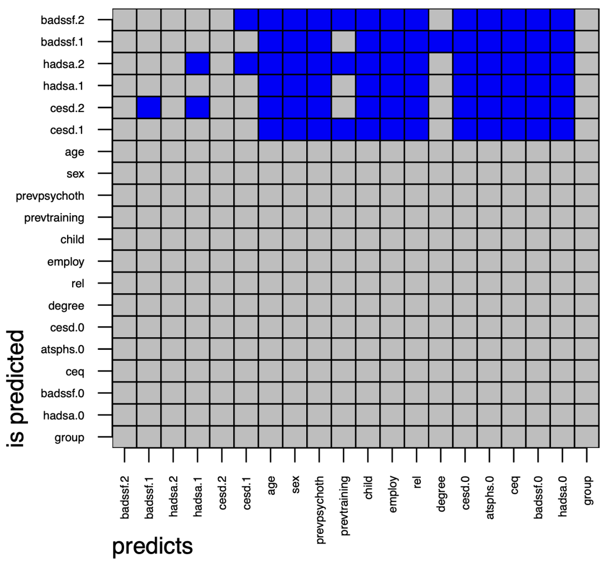

This predictor matrix is the core of our imputation model, and it makes sense to include it in our RCT analysis report (for example in the supplementary material). This is because the matrix tells us exactly which predictors are used to impute which variable with missing values. To better understand its structure, it helps to go through each row of the matrix step by step. As indicated by the y-axis label, the rows tell us the variable that should be imputed, while the columns indicate the predictors that will be used. However, variables only serve as predictors when their respective field is highlighted in blue.

This allows us to observe a few general patterns. First, we see that the `quickpred` function selected most baseline co-variates as predictors for the missing post-test and follow-up assessments. One exception are `prevtraining` and `degree`, which have been removed for some variables based on their correlation and proportion of usable cases. Second, we see that predictors are only defined for a few variables within our data. These are the outcome assessments that actually have missings, while the rest are complete baseline measurements that do not have to be imputed.

Lastly, the matrix also points us to a *weakness* of our imputation model. To estimate missing values in the outcome variables, most of the information we can rely on is based on the baseline characteristics of a person. Only some post-randomization measurements can be used as predictors, which is the result of us setting the minimal proportion of usable cases to 5%. This is not uncommon with RCTs, in which data is often missing monotonously. This should remind us that multiple imputation is not a panacea and that we will need a few sensitivity analyses to assess the robustness of our results. We will elaborate on this point in sections 4.2.3 and 5.1.3.

With our predictor matrix set up, we now have to determine the imputation *method* we should use. As shown in Table 1, several techniques are available for this, and we have to decide which one fits best with the type of data we aim to impute. A good first start is to let `mice` preselect a fitting imputation method for us, which can then be changed if necessary. To do this, we have to run a null imputation again, but this time we already provide our predictor matrix in the `predictorMatrix` argument. We can then extract the `method` object, which contains the imputation technique that `mice` selected for us.

```
imp0 = mice(imp.data, maxit = 0,
            predictorMatrix = pred)
imp0$method
```

```
## badssf.2      badssf.1      hadsa.2      hadsa.1       cesd.2
##    "pmm"         "pmm"        "pmm"        "pmm"        "pmm"
##   cesd.1          age          sex prevpsychoth prevtraining
##    "pmm"           ""           ""           ""           ""
##    child       employ          rel       degree       cesd.0
##       ""           ""           ""           ""           ""
## atsphs.0          ceq     badssf.0      hadsa.0        group
##       ""           ""           ""           ""           ""
```

We see that for all variables with missings, predictive mean matching ("pmm") has been chosen. Given that all variables with missings are continuous, this might indeed be a good default strategy.

Alas, we are not done here. Since we want to impute separately for each group, our imputation method has to be slightly adapted. First, we create an index named `no.missings`, which indicates if a variable has no missings. This is true for all variables for which the imputation method was set to `""` by `mice`.

```
# Find variables without missings
no.missings = imp0$method == ""
```

Next, we have to replace the imputation method of all variables *with* missings from `"pmm"` to `"bygroup"`, which tells the `mice` function to impute separately by trial group. We save the resulting object as `imp.method`.

```
# Set imputation method to "bygroup"
imp0$method %>%
  replace(., . != "", "bygroup") -> imp.method
```

Now that we have an object that tells `mice` to impute by groups, we also need another object which specifies the actual imputation technique used within each group. To do this, we extract all elements from the original vector of imputation techniques in `imp0` associated with variables with missings, and then convert them to a `list` element. We save the resulting object as `imp.function`.

```
# Define the actual imputation method for all
# "bygroup" variables
imp0$method[!no.missings] %>% as.list() -> imp.function
```

Lastly, we have to produce a `list` element that encodes the *grouping variable* for each imputed variable in our data (in our case, this is `group`). We can achieve this using the code below; please note that the `purrr` package (Henry and Wickham, 2020), which is part of the tidyverse, has to be loaded first for this to work. We save the resulting list as `imp.group.variable`.

```
library(purrr)
rep("group", length(imp.method[!no.missings])) %>%
  set_names(names(imp.method[!no.missings])) %>%
  as.list() -> imp.group.variable
```

Preparing these different lists and vectors is quite tedious. Fortunately, we now have all the ingredients we need to run our penultimate call to `mice` in which the data are finally imputed.

There are a few things to mention here. First, we specify the `predictorMatrix` argument again and provide our predictor matrix `pred`. In the `method` argument, we plug in our `imp.method` object, which tells `mice` that imputations should be performed by group. The `imputationFunction` argument is used to specify the actual imputation technique that is used within the two groups. In the `group` argument, we provide our list specifying the grouping variable for each outcome with missings.

The `m` argument specifies the number of imputed data sets we want to generate. Here, we use `m = 50`. Since we want to conduct an actual imputation now, we set the `maxit` argument, specifying the number of MICE iterations, to 50. Lastly, we can also specify a seed within `mice`. This allows us to offset R's random number generator, which is helpful if we want to keep our results reproducible at a later stage. Please also note that the `miceadds` package needs to be loaded for the code below to work.

> **!** When specifying the `mice` function, please always make sure that its results are **saved to an object** using the assignment operator. If results are not assigned to an object, the imputation will run regularly, but the imputed data sets will not be saved. In our example below, we save the results as `imp`.

```
mice(imp.data,
     predictorMatrix = pred,
     method = imp.method,
     imputationFunction = imp.function,
     group = imp.group.variable,
     m = 50, maxit = 50,
     seed = 123) -> imp
```

If everything has been correctly specified, the `mice` function will now start imputing, showing the progress in the console. Depending on your hardware the imputation should take approximately 5-10 minutes to finish. The current code only saves the imputed data locally in our environment, so it makes sense to also save it as an external file right away. A good way to do this is to use the `save` function, which allows to save the `imp` object with a ".rda" extension. This creates an *R Data* file on our computer, which is particularly easy to import into R later on.

```
save(imp, file="imp.rda")
```

Next, it is important to check our imputations for potential problems. We want to confirm that, for all imputed variables, the MICE algorithm has converged to a stable solution. A good way to diagnose potential convergence issues is to generate a *trace plot* for all imputed variables:

```
plot(imp, layout = c(4, ceiling(sum(!no.missings)/2)))
```

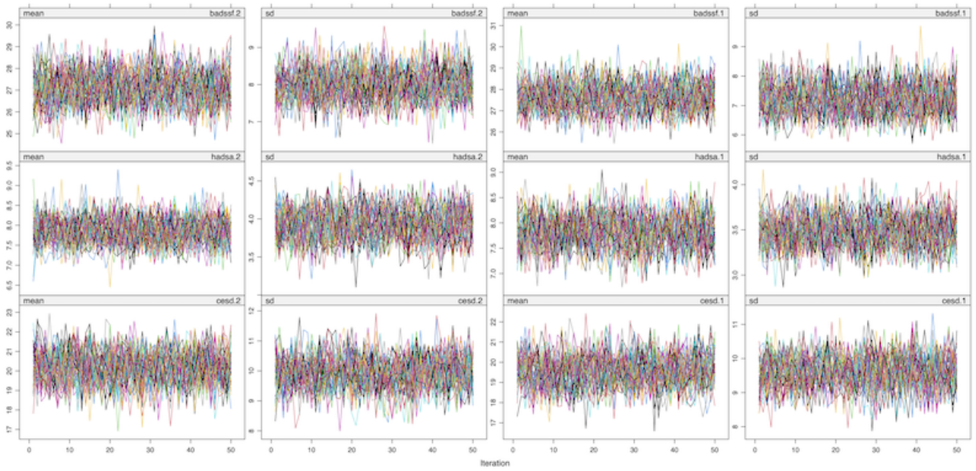

The trace line plot shows, for all imputed variables, the mean and standard deviation of the imputed values $\dot{\mathbf{Y}}_{\text{mis}}$ on the y-axis, while the x-axis shows the specific MICE iteration at which this value was obtained. Since we generated m=50 imputation sets, 50 lines are shown in each plot.

If the algorithm has converged, our imputed values should hover randomly around some specific value, with no particular trend visible over time. Furthermore, we also want that all trace lines are properly mixed and no imputation set displays consistently higher or lower values than others.

A good metaphor, as mentioned by Lunn and colleagues (2012, chap. 4.4.1), is that the trace lines should appear like a "fat hairy caterpillar". This is arguably the case in our example, which increases our confidence that the imputations are usable for further analyses.

Examples of Non-Convergence

Given that the trace lines in our example above behaved quite "orderly", we also simulated examples in which this is not the case (cf. van Buuren, 2018, chap. 4.5.7).

Panel **A** below displays *slow convergence*, which can sometimes occur if the number of missings is very large. As we can see, the imputed values display a clear downward trend roughly until the 50th iteration; after that, the chains become stationary. In this scenario, the MICE algorithm does converge, but it is necessary to have a sufficient amount of iterations (approximately 50-100 in this case) before trustworthy estimates trickle through.

A much more troublesome case of actual *non-convergence* is displayed in panel **B**. In this scenario, even after 200 iterations, no clear stationarity is reached: the different trace lines scatter around without any visible pattern, and the chains do not mix either. Trace lines like this would be cause for concern but thankfully are rarely seen in practical applications.

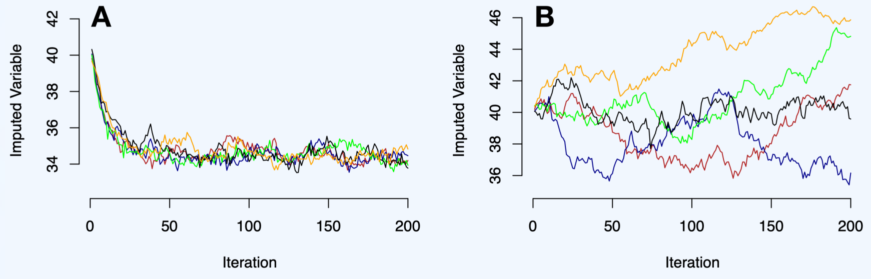

Another thing we can do to check our imputations is to create a density plot. The `mice` package has a special function called `densityplot` for this purpose. For now, we focus on inspecting the imputed value distributions in our primary outcome, the CES-D score at post-test and follow-up. The `densityplot` function also allows us to define a formula within the call, which can be used to generate separate plots for the intervention and control group:

```
densityplot(imp, ~ cesd.1 + cesd.2 | as.factor(group))
```

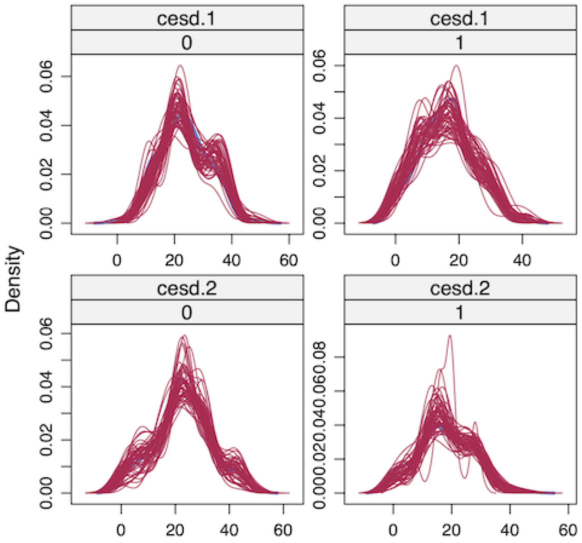

These plots show, in red, the distribution of the imputed values for each of the 50 imputation sets. Looking closely, we also see the original distribution of observed outcomes, which is plotted in blue. Typically, a plausible imputation model should produce imputed values $\dot{\mathbf{Y}}_{\text{mis}}$ that have a distribution similar to the one of $\mathbf{Y}_{\text{obs}}$, as is the case here.

However, please note that under MAR, systematic differences in the distribution of $\dot{\mathbf{Y}}_{\text{mis}}$ and $\mathbf{Y}_{\text{obs}}$ are also plausible (e.g., differences in the mean or dispersion of values). It is *extreme* divergences or an excess of very unrealistic results that may point to potential problems with the imputation model. The distribution of imputed and observed values can also differ substantially when only a few values had to be imputed. If only, say, 3-4 values were imputed, this of course makes it much more likely that their density does not follow a bell-type shape like many of the red lines in our example above do.

4.2.3 | Reference-based Imputation

In the last section, ran our first imputation model using the MICE algorithm. As we mentioned before, these imputations were generated under the MAR assumption. We imputed values separately for the intervention and control group, assuming that the information recorded for both groups is sufficient to generate a valid approximation of the missing values.

The MAR assumption can be a plausible "first start" to handle missing outcomes, provided a sufficient amount of meaningful auxiliary variables is available. However, it is by no means guaranteed that the data are indeed MAR, and that our imputations are valid. Typically, it is not advisable to blindly trust that the MAR assumption will hold in our particular data set; instead, we should conduct *sensitivity analyses* that account for potential departures from MAR (Tan et al., 2021; Cro et al., 2020).

This means that additional imputation methods should be used that handle missing values under the MNAR assumption. This is a challenging task, since we do not know, and cannot prove, to which extent our missingness pattern is related to the missing values themselves.

Several techniques have been developed to handle MNAR data in the past. One example are *selection* (Little, 2014; Heckman, 1979) and *pattern-mixture models* (Thijs et al., 2002; Glynn et al., 1986). These approaches add a shift parameter δ to the imputed values, thus modeling the assumption that missing values differ systematically from the recorded ones. This allows us to, for example, model the assumption that patients with missing values had systematically worse outcomes at post-test, and that this is why they are missing. This adjustment requires one to assume a plausible value of δ, or a range thereof.

A related method that is sometimes used in pharmaceutical research is *tipping point* analysis, in which one studies how severely the missing values would have to differ from the observed ones to render the effect of an analysis non-significant (Liublinska and Rubin, 2014).

A more recent method that lends itself particularly well to clinical trial evaluations is *controlled multiple imputation*, specifically the so-called *reference-based imputation* methods as described by Carpenter, Roger and Kenward (2013). The particular type of reference-based imputation that we will implement next in our own example trial uses the so-called **jump-to-reference** (J2R) approach (Carpenter et al., 2013; White et al., 2020). So let us take some time to explain this method a little.

At first glance, the J2R approach closely resembles a MICE-based imputation under the MAR assumption. It selects a set of covariates from our data to impute missing values, and, like MICE, it is also an MI technique that generates multiply imputed data. After fitting the desired models in all data sets, estimates are then pooled into one result. This is often (but not always; Bartlett and Hughes, 2020; Bartlett, 2023) done using the same methods that are used for "conventional" MI.

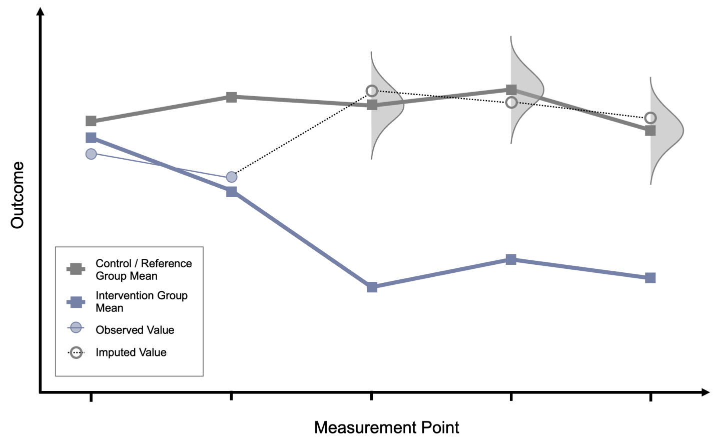

Figure 6: *Illustration of reference-based imputation ("jump-to-refence" approach).*

The key distinction is that J2R relies on specific assumptions about the trajectory of patients' outcomes starting from the point at which data are missing. The J2R approach assumes that, in terms of their outcomes, individuals in the intervention "jump" to the reference group (i.e., the control group) once they are lost to follow-up (see Figure 6). This assumption is very plausible in trials that do not include *off-treatment* assessments, as is often the case in pharmacological research. In such studies, individuals are not followed up anymore once they stop using the treatment; for example when they discontinue the medication under study.

Obviously, in this scenario, the missing outcome values are likely to differ systematically from the recorded ones: the patients who do not respond well, or experience negative side effects, are also the ones most likely to have missing outcomes. The J2R approach tries to account for this by imputing values *as if* the participant was now part of the control group. Thus the name "jump to reference".

Computationally, this is typically done by fitting a Bayesian multivariate normal model with meaningful baseline covariates in both groups. Missing values of intervention group participants are then drawn from the distribution of the reference group to generate imputed values for each specific time point, and based on the participant's baseline characteristics.

The J2R method offers a sensible method to impute missing values in trials without off-treatment assessments. In this case, it is often even used as the main imputation model. However, it may be less

appropriate in other contexts. The J2R method essentially assumes that intervention participants lose all treatment benefits they may have experienced before being lost to follow-up. This is, arguably, quite an extreme position (Iddrisu and Gumedze, 2019). It is also unclear how sensible such an assumption is in, for example, psychological treatments, which typically do not have a clear-cut dose-response relationship (Robinson et al., 2020; Shalom and Aderka, 2020). Furthermore, many trials at least *try* to obtain outcome assessments from the intervention group no matter if patients used the treatment as intended or not. This approach was also followed in our example trial. If a considerable amount of off-treatment assessments are available, this also strengthens the plausibility of an imputation under MAR.

Nevertheless, the J2R method might lend itself quite well to sensitivity analyses. This is *because* its assumptions are quite extreme. Analyzing J2R-imputed data allows us to obtain an estimate of the treatment effect under a plausible worst-case scenario in which we assume that all intervention participants who dropped out of our trial did so because the intervention was ineffective for them.

There are now several packages that allow to implement reference-based imputation in R, for example, `rbmi` (Gower-Page and Noci, 2022) or `mlmi` (Bartlett, 2021). However, the package we will be using here is `RefBasedMI` (McGrath, 2023), which is based on the *mimix* program in STATA.

At the time we are writing this, the `RefBasedMI` package is not available on CRAN. That means that we need to install the package directly from its online repository. To do this, we have to install and load the `remotes` package (Csárdi et al., 2021) first, after which we can use the following code to install `RefBasedMI`.

```
remotes::install_github("UCL/RefBasedMI")
library(RefBasedMI)
```

Next, we have to reshape our trial data set to a format the `RefBasedMI` can work with. Currently, our data is in the *wide* format: each participant of the trial is represented by exactly one row. We now have to convert the data to the *long* format, where each participant has two rows: one for the post-test assessment, and another one with the 3-month follow-up scores.

Reshaping data frames in R is not an easy task, especially for beginners. Here we show code based on the `pivot_longer` function (which is included in the tidyverse) that can be used to bring our data into the desired format. Please note that this approach is "re-usable" for other trial data sets too, as long as the same coding guidelines are followed.

First, we have to create a new numeric `id` variable in our data set, like so:

```
data$id = 1:nrow(data)
```

Next, we use the `pivot_longer` function to reshape our data into the desired long format. We save the results as `data.long`

```
pivot_longer(data = data,
             ends_with(c(".1", ".2")),
             values_to = "value",
             names_to = c("outcome", "time"),
             names_pattern = "(.*).(.)") -> data.long
```

The reshaping creates a new variable in our data set called `time`, which encodes the post-randomization measurement point at which a value was obtained. We can examine this variable a little further:

```
data.long$time
```

```
##  [1] "1" "1" "1" "2" "2" "2" "1" "1" "1" "2" "2" "2" "1" "1" "1" "2" [...]
```

We can see that the value of this variable is either "1" or "2", indicating if the value was obtained at post-test or 3-month follow-up. Using the mutate function, we now recode the two values to 7 and 12, respectively, indicating the actual number of weeks after randomization at which participants were assessed.

```
data.long %>%
  mutate(time = recode(time, `1` = 7, `2` = 12)) -> data.long
```

Next, we have a glimpse at the reshaped data. For now, we focus on the first ten rows and a few relevant columns.

```
data.long[1:10, c("id", "cesd.0", "group",
                   "outcome", "time", "value")]
```

```
##     id cesd.0 group outcome  time value
##  1   1     24     0 badssf      7    20
##  2   1     24     0 hadsa       7     5
##  3   1     24     0 cesd        7    33
##  4   1     24     0 badssf     12    18
##  5   1     24     0 hadsa      12    10
##  6   1     24     0 cesd       12    30
##  7   2     38     0 badssf      7    NA
##  8   2     38     0 hadsa       7    NA
##  9   2     38     0 cesd        7    NA
## 10   2     38     0 badssf     12    NA
```

We see that, for each of our outcomes assessed after randomization (badssf, hadsa, cesd), the data frame now provides values for the post-test measurement (time=7) and follow-up (time=12) in the value column. Importantly, note that the baseline measurement of, e.g., the CES-D is included as a separate column (cesd.0), which has the same value across all measurement points and outcomes for each participant.

The reference-based imputation method implemented in RefBasedMI is less flexible than mice. For example, it is necessary that all co-variates that we want to include in the imputation model are completely recorded. Furthermore, reference-based imputations can only be generated for one outcome at a time, which means that we have to run the function separately for the CES-D, BADS-SF and HADS-A values. Since we use J2R as a sensitivity analysis in this example, we only focus on our primary outcome for now, the CES-D values. This means that we can filter out all cesd measurements using the outcome variable. We save the resulting data frame as data.long.cesd.

```
data.long %>%
  filter(outcome == "cesd") -> data.long.cesd
```

We are now set and ready to perform our J2R imputation using the RefBasedMI function. Let us go through the required arguments step by step:

- In the covar argument, we can specify the baseline variables conditional on which imputations should be generated. Here, we use the same baseline variables as were included in the predictor matrix when using MICE (see section 4.2.2).
- The depvar argument can be used to specify the outcome variable we want to impute; in our case, this is the value column.
- The treatvar, idvar and timevar arguments specify the treatment, ID and assessment point variable, respectively.

- In the `method` argument, we specify the imputation approach we want to apply. Several options are available, for example `"MAR"` for a "simple" MAR imputation, `"CR"` for "copy reference", which is another type of reference-based imputation; or `"J2R"`, which implements the J2R approach we want to use for our example.
- In the `reference` argument, we have to specify the value of the group variable provided in `treatvar` that corresponds with the reference/control group. In our case, this is `0`, since this value has been used for participants in the waitlist control group.
- The `M` argument controls the number of imputation sets that are generated (m=50 in our case), while the `seed` argument can be used to set a random number generator seed.

We now assemble these pieces in our call to `RefBasedMI`, after which we use the `as.mids` function in a pipe to convert the results to a `mids` object. This is helpful because this is the same type of object that is also created by `mice`, which means that the resulting multiply imputed datasets can be analyzed in the same way. We save the result as `imp.j2r`.

```
imp.j2r <- RefBasedMI(data = data.long.cesd,
                      covar = c(age, sex, prevpsychoth,
                                prevtraining, child, employ,
                                rel, cesd.0, atsphs.0, ceq,
                                badssf.0, hadsa.0),
                      depvar = value,
                      treatvar = group,
                      idvar = id, timevar = time,
                      method = "J2R", reference = 0,
                      M = 50, seed = 123) %>%
                   as.mids()
```

This implementation should be faster than `mice`, with imputations generated within 1-2 minutes. It is advisable to save the imputed data right away as an `.rda` file:

```
save(imp.j2r, file="imp.j2r.rda")
```

Congeniality of the Imputation & Analysis Model

Introduced by Meng (1994), the term *congeniality* refers to the idea that our imputation and analysis model should be "of the same kind". One of the advantages of MI is that it allows us to analyze our data using the same model we would have used if all data had been observed; this is also known as the *substantive analysis model.*

Congeniality means that the assumptions we make in our imputation model should be *compatible* with what we assume about our data in the substantive analysis model. For this reason, congeniality is also sometimes referred to as substantive model *compatibility* (Bartlett et al., 2015; Quartagno and Carpenter, 2022b).

One example of this are clustered data (e.g. individual participant data from several studies, or multi-centre RCT data). In this case, a multilevel imputation model has to be used, because the substantive analysis model also assumes a clustered data structure (e.g. if we used mixed-effects or IPD meta-analytic models); otherwise, estimates that we obtain from the model may not be valid. A package that is designed particularly for multilevel imputation models is `mitml` (Grund et al., 2021). However, this package can also be helpful when the data are not clustered, and we will use some of its functionality later in this tutorial.

There are now several packages in R that implement so-called *substantive model compatible multiple imputation* (SMC-MI), which ensures that the imputations are

> strictly compatible with the substantive analysis model we want to apply in the next step; for example the `smcfcs` package (which uses an FCS-based approach; Bartlett et al., 2022), or the `JointAI` package (which uses a joint Bayesian approach in which data are imputed and analyzed in one step; Erler et al., 2021). These implementations may be beneficial if our substantive analysis model assumes, e.g., complex interactions or non-linear relationships.

4.3 | Rubin's Combination Rules

After having generated our multiply imputed data, we can now proceed to the next step, in which our analysis model is fitted in each data set and then pooled. This adds another level of complexity: when the same analysis is conducted in m data sets simultaneously, this also means that we obtain m results, which differ more or less from each other due to our imputation uncertainty. Our goal, however, is to come up with one result (e.g. one estimate of the mean difference between groups, and whether this difference is significant) that we and others can interpret. To generate such a pooled result across all imputed data sets, we use a set of formulas that are commonly known as **Rubin's Rules** in the literature (Rubin, 2004; Barnard and Rubin, 1999; van Buuren, 2018, chap. 2.4, 5.2).

Rubin's combination rules take into account that all estimates we can obtain from our data are subject to uncertainty. In the case of multiply imputed data, there are *two* reasons for this:

1. Participants in our trial are only a *sample* from a much greater population that we want to study, which means that some degree of *sampling error* can be expected.
2. Our data contain missing values that had to be imputed. We do not know the true value of these imputations, which means that they also make our estimates less certain. This uncertainty is reflected by the fact that multiply imputed values may differ slightly between imputation sets.

In essence, the goal of Rubin's rules is to generate pooled estimates (and, even more important, confidence intervals around them) that properly integrate these two sources of variation. It allows to obtain results that reflect both our uncertainty due to sampling error, which occurs even if all data had been recorded and the additional uncertainty due to the fact that some of our data are mere imputations; not "real" values that we know for sure.

Let us introduce some notation. Let Q be a true value (or a vector of true values) in the population that we want to estimate (e.g. a population mean, regression coefficient, or the like). Due to the reasons we named above, Q is unknown and must be approximated by an estimate thereof, $\hat{Q}$. This is only possible using the observed values Y_{obs}. The expected value $\mathbb{E}$, the best possible approximation of Q given our observed values Y_{obs} is (van Buuren, 2018, chap. 2.3.2):

$$\mathbb{E}[Q|Y_{\text{obs}}] = \mathbb{E}\left[\mathbb{E}[Q|Y_{\text{obs}}, Y_{\text{mis}}] \,\middle|\, Y_{\text{obs}}\right] \tag{11}$$

This formula looks very abstract and complex, but it helps to read it from the outside to the inside. It tells us that the best approximation of Q given Y_{obs} is to take the *expected value of the expected values* in all imputed data sets. Let us make this more concrete: if Q is, for example, a population mean, we can take the *average* of *all the means* calculated in each of our multiply imputed data sets to obtain the best possible approximation given our data. We can also formalize this. Let $\hat{Q}_\ell$ be the estimate of Q in some imputation set ℓ, where m is the total number of imputation sets we generated. The "pooled" estimate of Q is thus:

$$\bar{Q} = \frac{1}{m}\sum_{\ell=1}^{m}\hat{Q}_\ell. \tag{12}$$

This formula can be used in practice to obtain a pooled point estimate within our multiply imputed data: we calculate the value of the point estimate (e.g., a sample mean or regression weight)

in each imputed data set individually, and then take the average of these calculated values across all imputation sets. Please note that this simple aggregation method cannot be used if we want to pool test statistics (e.g. t or F values), and it is also not advisable if we want to combine correlations. We will cover how to deal with such estimates later in this chapter.

Naturally, we are not only interested in point estimates but also in their uncertainty. To calculate P values and confidence intervals around our estimates, we also need to calculate the pooled *variance* of Q in our multiply imputed data. Here, we cannot simply analyze all data sets as if they would contain no missings and then average the results. As mentioned before, we have to take into account that there are *two sources of variation* that determine the uncertainty of our estimate:

$$\mathrm{Var}[Q|Y_{\text{obs}}] = \underbrace{\mathbb{E}\left[\mathrm{Var}[Q|Y_{\text{obs}}, Y_{\text{mis}}] \,\middle|\, Y_{\text{obs}}\right]}_{\substack{\text{mean of the variances across MI sets} \\ \rightarrow \textbf{Within-Variance } (\bar{U})}} + \underbrace{\mathrm{Var}\left[\mathbb{E}[Q|Y_{\text{obs}}, Y_{\text{mis}}] \,\middle|\, Y_{\text{obs}}\right]}_{\substack{\text{variance of the means across MI sets} \\ \rightarrow \textbf{Between-Variance } (B)}} \tag{13}$$

As shown in the equation above, these two components are the "general" variance $\bar{U}$ that we find *within* each imputed data set, and the variation B of our estimate of Q *between* imputation sets. These components coincide with the two sources of variation we discussed before: $\bar{U}$ represents the (averaged) variance due to sampling error, while B is the variation due to imputation uncertainty. In practice, the pooled variance $\hat{V}$ can be calculated using Rubin's rules:

$$\begin{aligned}\hat{V} &= \overbrace{\left(\frac{1}{m}\sum_{\ell=1}^{m}\bar{U}_{\ell}\right)}^{\bar{U}} + \left(1+\frac{1}{m}\right)\overbrace{\left(\frac{1}{m-1}\sum_{\ell=1}^{m}(\hat{Q}_{\ell}-\bar{Q})(\hat{Q}_{\ell}-\bar{Q})'\right)}^{B} \\ &= \bar{U} + \left(1+\frac{1}{m}\right)B \\ &\Rightarrow \bar{U} + B \quad \text{as} \quad m \rightarrow \infty\end{aligned} \tag{14}$$

It is not essential to understand every detail of this equation. The core part is indicated by the braces, which shows that using Rubin's rules, we combine the within-variance $\bar{U}$ and between variance B to obtain the correct estimate of our total variance $\hat{V}$. The parts of the equation displayed in blue show a third source of variation: in practice, we can only generate a finite amount of imputation sets m, and this *simulation variance* adds another source of uncertainty that is accounted for in the formula. The equation above also shows that, as m goes to infinity, this variation source drops out of the equation, leaving only the $\bar{U}$ and B components that we discussed before. This part of the Rubin formula is one of the reasons why it is sensible to have a larger number of imputed data sets since this means that the term $(1 + 1/m)$ becomes smaller. Especially when very accurate variance estimates are necessary, a high number of imputation sets is therefore recommended. A low m leads to confidence intervals that are slightly wider than when $m \rightarrow \infty$ (statisticians call this lower *efficiency*). However, this difference is typically manageable in practice.

4.3.1 | Metrics for Parameter Estimates in MI

There are a few metrics that can be derived from the estimate of the within- and between-variance $\bar{U}$ and B. These metrics allow us to better understand the extent to which missing data has impacted the uncertainty of our pooled results. One of these metrics is the *relative increase in variance due to non-response*, or RIV, which quantifies the amount of between-imputation variation of Q in relation to the "real" variance in our imputed variable. It is calculated like this:

$$r_Q = \frac{B_Q/m + B_Q}{\bar{U}_Q}. \tag{15}$$

Whereby values of $r_Q > 1$ indicate that the imputation variance is greater than the average

within-variance in our data.

Another metric is the *fraction of missing information due to non-response* (FMI). This is the fraction of information about Q that was "lost" due to our imputation uncertainty. It is calculated using the following formula:

$$\gamma_Q = \frac{(r_Q + 2)/(\nu_Q + 3)}{1 + r_Q}. \tag{16}$$

Where r_Q is the RIV from above, and ν_Q (*nu*) are the degrees of freedom in the estimation of Q. There is a special way in which these degrees of freedom are calculated in analyses of multiply imputed data, as we will see in the next section.

4.3.2 | Degrees of Freedom in MI Analyses

The degrees of freedom of a model are typically defined as the number of observations minus the number of model parameters: $\nu = n - k$ (e.g., for the mean: $\nu = n - 1$). With MI, some elements of n are only estimates of observations that were not recorded, which means that ν also has to be corrected accordingly for this:

$$\nu_{\text{(MI)}} = (m - 1)\left(1 + \frac{1}{r^2}\right) \tag{17}$$

Where r is the relative increase in variance we discussed in the previous chapter. This formula has a special property that we see when setting r, the RIV, to zero. When $r = 0$, this means that all data has been recorded and that there has been no increase in variance due to non-response. In this scenario, the value of $\nu_{(MI)}$ returned by the formula is infinity:

$$\lim_{r \to 0} \nu_{\text{(MI)}} = \infty \quad \text{and} \quad \lim_{r \to \infty} \nu_{\text{(MI)}} = m - 1 \tag{18}$$

Put differently, the formula works under the assumption that the degrees of freedom of the complete data model are infinitely large. This large-sample approximation is only sensible when our sample is relatively big.

For small samples, an adapted formula needs to be used (Barnard and Rubin, 1999). This requires us to first determine the "hypothetical" degrees of freedom of our model if our data had been fully recorded. We call this the complete data degrees of freedom, ν_{com}, which are calculated from the number of observations n and parameter number k:

$$\nu_{\text{com}} = n - k \tag{19}$$

This way, we can determine the degrees of freedom of the observed values, ν_{obs}:

$$\nu_{\text{obs}} = \frac{\nu_{\text{com}} + 1}{\nu_{\text{com}} + 3}\nu_{\text{com}}\left(1 - \frac{r}{r+1}\right), \tag{20}$$

which in turn are needed to obtain a small-sample-corrected version of $\nu^*_{\text{(MI)}}$:

$$\nu^*_{\text{(MI)}} = \frac{\nu_{\text{(MI)}}\nu_{\text{obs}}}{\nu_{\text{(MI)}} + \nu_{\text{obs}}}. \tag{21}$$

MI Degrees of Freedom: A Practical Note

The unadjusted degrees of freedom formula is the default used by the `mitml` package that we will be using in our analysis later on. Please note that, if this formula is used, the degrees of freedom of a parameter can be *larger* than the total number

of observations n. Also, since the degrees of freedom are merely approximated by the formulae above, the value of ν also does not have to be a natural number (values like, e.g., ν=256.5669 are also perfectly possible).

4.3.3 | MI-Based Confidence Intervals

The calculated degrees of freedom can be used to construct a confidence interval around our point estimate. For the inference of *scalars* (i.e., when $\bar{Q}$ is a single value), a t-distribution is typically assumed. The t-value is determined based on the reference value of our null hypothesis Q_0, which is usually 0:

$$\frac{\bar{Q} - Q_0}{\sqrt{\hat{V}}} \sim t_\nu \tag{22}$$

Whereby the test of t depends on the degrees of freedom ν. Using the calculated MI degrees of freedom $\nu_{(\text{MI})}$, we can then calculate a confidence interval around $\bar{Q}$ that accounts for our imputation uncertainty:

$$\bar{Q} \pm t_{\nu^{(*)}_{(\text{MI})},0.975} \times \sqrt{\hat{V}}. \tag{23}$$

The `mitml` package that we will be using in this analysis contains functions that allow constructing such confidence intervals directly for us.

4.3.4 | Analysis of MI Data: Caveats & Special Cases

Before we complete this chapter, we want to cover a few special cases and caveats to keep in mind when pooling the results obtained from multiply imputed data.

Our first point pertains to the aggregation of **test statistics**, such t, χ^2, or F values obtained by some model. Such test statistics are often calculated in practice, but it is not possible to simply pool them using the arithmetic mean, as we did with $\bar{Q}$. The reason for this is that the value of a test statistic depends on the variance and degrees of freedom of our model. However, as we learned, when some missing values had to be imputed, the overall variance increases because imputation uncertainty is added to our estimates. Thus, the pooled value of the test statistic also needs to reflect this. To avoid overestimating test statistics, special aggregation formulas must be used. Luckily, there are now several implementations in R that can do this for us, such as the `micombine.chisquare` and `micombine.F` functions in `miceadds`. In our hands-on analysis, we will showcase how to implement these functions in practice.

Another special case in MI analyses are **correlations**. The value of a correlation ρ cannot exceed the maximum values $[-1; 1]$. This means that the variance of ρ is restricted the closer ρ gets to 1 or -1. As a result, we cannot assume that ρ is asymptotically normal. Thus, to aggregate correlations, it is not advisable to simply calculate the mean of all correlations we obtained in the multiply imputed data. Instead, we should first apply a variance-stabilizing transformation by calculating what is known as Fisher's z. In R, such a transformation is applied automatically if we use the `micombine.cor` function, which is also part of the `miceadds` package.

Lastly, we also want to bring attention to a commonly used, but generally inappropriate approach to dealing with multiply imputed data. In practice, it is often seen that analysts **aggregate all MI sets into *one* data set** without missings, and then proceed to conduct their analyses in this single data set. Naturally, this makes an analysis using conventional statistical software a lot easier; but this approach should definitely be avoided from a statistical perspective. Aggregating into one dataset "pretends" that there is no imputation uncertainty; this leads to incorrect (that is, anti-conservative) P values, confidence intervals, test statistics, and so forth. An analysis of aggregated data should only be performed if no statistical tests or quantification of parameter uncertainty is needed. Yet, this is rarely the case in practice.

4.4 | Functional Programming

The last chapter was quite theoretical, and we can assure you that things will become more hands-on from now on. In this section, we will cover a more practical issue in the analysis of multiply imputed data, and how we can solve it using R.

As we have learned, an appropriate analysis of MI data requires us to fit the desired analysis not only once, but in each of the m generated data sets in parallel. This is easier said than done. If we want to perform some operation in several data sets at the same time, even presumably easy analysis steps can become markedly more complex.

To illustrate this, let us go back to our `imp` object that we created in Chapter 2.2.2. This object contains the multiply imputed data that we generated using MICE. As a next step, we use the `mids2mitml.list` function in the `mitml` package to convert this object to a simple list, in which each list element contains one of the imputed sets. We save the result as `implist`.

```
library(mitml)
implist <- mids2mitml.list(imp)
```

How can we analyze all the imputed data sets in this list simultaneously? Imagine that, for example, we want to calculate the mean of our primary outcome variable `cesd.1` in all data sets, and then average them to get a pooled estimate. A commonly used approach is to use a *for loop* for this purpose. This allows us to go through each list element (viz., imputed data set) one after another, calculate the mean, and save it in a new variable that we will call `means` here. After that, we can take the average of `means` to obtain our final result.

```
# Create an empty vector in which our results will be stored
means <- vector()

# Loop through all m=50 imputation sets
for (i in 1:50){
    means[i] <- implist[[i]] %>% pull(cesd.1) %>% mean()
}
mean(means)
```

```
## [1] 19.77795
```

Do not worry if you have never heard of `for` loops before, or if you do not understand everything about the code that we used here. The example above only goes to show that a rather straightforward operation (taking the mean of a variable) can become tricky when we have to apply it in all data sets simultaneously. The code above is quite complex. Furthermore, if we had performed a more involved analysis than simply extracting the mean, we would have also seen that this approach can lead to quite long computation times.

A better approach to perform operations in multiply imputed data is to use **functional programming**. In a functional programming approach, we use a single function that applies some operation to all imputation sets for us at the same time. Base R contains functions like `apply`, `mapply`, `vapply`, or `Reduce` to do this. However, a particularly user-friendly and broadly applicable option are the `map` functions included in the `purrr` package (Henry and Wickham, 2020). To illustrate this, here is how we can apply the same operation from before using `map`:

```
implist %>% map_dbl(~mean(.$cesd.1)) %>% mean()
```

This code is less convoluted than our `for` loop, and it also runs faster.

In essence, every call to `map` requires two arguments: a `list` object in which each element contains a different imputation set, as well as the function we want to apply in each of these elements.

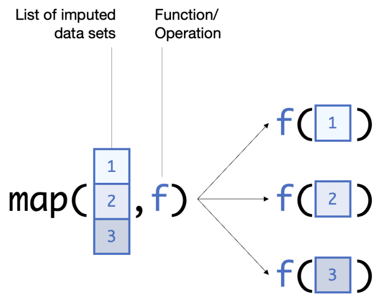

The inner workings of `map`*.*

Once we run the function, `map` will then automatically apply the defined operation in each set and provide us with a list of the results.

The `map` function comes in different "flavors", depending on the type of object that should be returned. The `map_dbl`, `map_chr`, `map_lgl` and `map_dfr` functions can be used depending on whether we want the results to be a numeric, character or logical vector, or a data frame, respectively. In all of the `map` functions, the second argument specifies the type of function that should be performed in all list elements. There are two ways to specify this function; the first is to supply a "fully functioning" (no pun intended) function. This function has exactly one argument `x`, whereby `x` represents one individual list element. Here is an example:

```
# 'x' represents the individual list element in 'list'
map(list, function(x) mean(x$variable))
```

The other option is to use a shortened notation, in which we use a tilde (∼) to indicate the start of the function, and a dot (`.`) to represent the individual list element. Here is the same example from above using this shortened notation:

```
# '.' represents the individual list element in 'list'
map(list, ~ mean(.$variable))
```

It typically requires a little practice until one becomes fully familiar with functional programming as implemented in `map`. Nonetheless, it is worth the effort: analyses of multiply imputed data require us to apply functions in lists of data sets all the time, which makes `map` an indispensable tool in practice.

■

PART 5 | Effectiveness Analysis

5.1 | Continuous Endpoints

It has been a long and winding road, but we have now arrived at the core of each RCT evaluation: the primary effectiveness analysis.

Put simply, the goal of this analysis is to confirm that the intervention has had an effect on the studied population. Yet, this is still very vague. In practice, effectiveness analyses require an *unambiguous* definition of the effect that should be tested. As we mentioned in the primer article, trial estimands provide a sensible approach to clarify precisely what our effectiveness analysis should estimate. As part of the estimand, we also have to define the outcome based on which

intervention effects should be tested. This outcome makes up the *primary endpoint* of our trial: a *specific* variable, measured at a *specific* time, using a *specific* instrument.

Importantly, the determination of the primary endpoint should not be within the purview of us, the trial analysts. Along with the trial estimand, primary endpoints must be specified *a priori*, before the actual patient recruitment has begun. If the primary endpoint was not pre-specified, this should be regarded as a **serious limitation** of the trial. In such instances, there is no way to prove that we have not simply used another outcome than originally intended to "prove" that our intervention works (a questionable research practice known as *outcome switching*).

In the primer article, we showed that the goal of clinical trials is to estimate the average treatment effect, or ATE. Through randomization, an unbiased estimate of the ATE can be obtained by examining the difference between the means of the intervention and control group (IG and CG) with regard to the primary endpoint Y:

$$\widehat{\text{ATE}} = \overbrace{\frac{1}{n_{\text{IG}}}\sum_{i=1}^{n_{\text{IG}}} Y_{\text{IG},i}}^{\text{Intervention Mean}} - \overbrace{\frac{1}{n_{\text{CG}}}\sum_{i=1}^{n_{\text{CG}}} Y_{\text{CG},i}}^{\text{Control Mean}} \tag{24}$$

$$= \hat{\mu}_{\text{IG}} - \hat{\mu}_{\text{CG}} \tag{25}$$

Thus, to show that the intervention had an effect, we have to test if the two group means differ significantly from each other (i.e., that $|\hat{\mu}_{\text{IG}} - \hat{\mu}_{\text{CG}}| > 0$). In RCTs, this is most commonly achieved via an *analysis of covariance* (ANCOVA; Montgomery, 2017; Dunn and Smyth, 2018, chap. 15.3, 2.9).

5.1.1 | Analysis of Covariance

ANCOVAs allow us to examine if two or more groups differ significantly from each other while we control for the influence of one or several covariates. In RCTs, these covariates are patient variables that we recorded before randomization; most commonly the baseline measurement of our primary outcome. In our case, this is depressive symptom severity as measured by the CES-D.

While ANCOVA is sometimes treated as a "special" method for experimental research, it is best understood as a specific type of **linear regression**. The regression formula can be written down like this:

$$Y_{ij} = \mu + \tau_i + \beta(X_{ij} - \bar{X}) + \epsilon_{ij} \tag{26}$$

In this equation, Y_{ij} is the outcome value of some patient j in treatment group i; τ_i is the estimated effect of some treatment group i (in our case, i can either be the Internet-based intervention group or the control group). X_{ij} is the value of a covariate that was recorded for patient j in group i. Lastly, ϵ represents the residuals of the model. These residuals are assumed to follow a normal distribution with mean zero and variance σ^2. This is commonly denoted as $\epsilon \sim \mathcal{N}(0, \sigma^2)$.

The ANCOVA model above assumes that our treatment variable is *effect coded*. In our example, this would mean that the treatment variable is either -1 or 1, depending on the treatment group (1 = Internet-based intervention, -1 = waitlist control). This ensures that the τ_i values sum up to zero:

$$\sum_{i=1}^{a} \tau_i = 0. \tag{27}$$

Where a is the number of treatment groups ($a = 2$ in our example). To test the treatment effect, ANCOVAs resort to the principle of *variance decomposition*, which tells us that the total variation in our data, as measured by the sum of squares (SS), can be partitioned into two components: the variation explained by our model, as well as the unexplained, or residual variation. We can summarize the relationship like this:

$$\text{Data} = \text{Model Fit} + \text{Unexplained Variance} \tag{28}$$

$$\text{SS}_{\text{Total}} = \text{SS}_{\text{Predictor}} + \text{SS}_{\text{Residual}} \tag{29}$$

The significance of the treatment effect is then tested by comparing the predictor and residual sum of squares while accounting for the (predictor and residual) degrees of freedom. This ratio is assumed to follow an F distribution, the shape of which is determined by the numerator and denominator degrees of freedom ν_{num} and ν_{den}. They are called like this because one of them, the number of parameters $p-1$, appears in the numerator of the fraction, while the other, the total sample size $n-p$, is part of the denominator:

$$F_{\nu_{\text{num}},\,\nu_{\text{den}}} = \frac{\text{SS}_{\text{Predictor}}/(p-1)}{\text{SS}_{\text{Residual}}/(n-p)} \tag{30}$$

Variation	Sum of Squares	Degrees of Freedom
Systematic	$\text{SS}_{\text{Predictor}}$	$\nu_{\text{num}} = p-1$
Random	$\text{SS}_{\text{Residual}}$	$\nu_{\text{den}} = n-p$
Total	SS_{Total}	$n-1$

Assuming that the sample size is fixed, high F values (i.e. a high value of $\text{SS}_{\text{Predictor}}$ combined with a low value of $\text{SS}_{\text{Residual}}$) mean that the findings are inconsistent with the null hypothesis of no effect, resulting in a low p-value.

Most introductory statistics books cover analysis of variance, and much of what we explained here may be nothing new to you. The crucial point to understand here is that equation 30 above explains *why* we control for covariates in ANCOVAs. To detect a significant treatment effect, we aim for a high F value; we want the numerator value to be large while minimizing the denominator. This is where covariate adjustment provides a solution: including variables that are predictive of the outcome, such as the baseline symptom severity, allows us to **reduce** the amount of **unexplained variation *within* the treatment and control group**. This means that $\text{SS}_{\text{Residual}}$ decreases, which in turn automatically leads to a higher F value.

Thus, the primary rationale for including covariates in ANCOVAs is to enhance our statistical power in detecting treatment effects. With the same sample size, we can get additional power "for free", just by controlling for factors that explain why patients have lower or higher outcome values *within* the different treatment groups.

There is a caveat here, however, that we can start to understand if we look closely at equation 30 again. In the equation, we can see that the denominator value is also influenced by the number of parameters p. Thus, controlling for covariates will only increase our power *if* they are actually predictive of the outcome, and thus reduce the residual variation in our data. If they do not, the only thing that increases is the number of parameters p, and the F-value actually *decreases*. Thus, it is important to select covariates carefully. In the next section, we explore this topic a little further.

5.1.2 | Covariate Adjustment

Although there is a general agreement that covariate adjustment is a reasonable method in trial analyses, what variables to control for is frequently less clear. Overall, how to best adjust for covariates in RCTs remains an open research question (Permutt, 2020; Kahan et al., 2014, 2016; Tackney et al., 2023; Morris et al., 2022). In this section, we want to provide some tentative guidance on how this topic can be approached in practice.

Generally speaking, our goal should be to include covariates that are prognostic of our primary endpoint. However, this does not mean that we should search for "optimal" covariates in our data, for example by examining their correlations with the outcome. Instead, adjustment variables should be pre-specified *a priori* to rule out that we simply added or removed covariates until the desired results were attained (Assmann et al., 2000).

In the previous section, our ANCOVA model only included one covariate, the baseline measurement of the outcome. However, it is very much possible and often indicated to include several variables. On the other hand, an excessive number of covariates (say 10 or more) should best be avoided. With a large number of covariates, it is likely that many will not explain a sensible amount of variation in our data. Yet they are still counted as additional parameters, thus counteracting our goal to achieve greater power through the adjustment. There are also other reasons that are nicely summed up by a guidance document from the European Medicines Agency (EMA, 2015):

> *"No more than a few covariates should be included in the primary analysis. Even though methods of adjustment, such as analysis of covariance, can theoretically adjust for a large number of covariates it is safer to pre-specify a simple model. [...] Results based on such a model are more likely to be numerically stable, the assumptions underpinning the statistical model are easier to validate and generalisability of the results may be improved."*

While these are more general recommendations, there is one case in which baseline adjustment is *strictly* required. If stratified block randomization was used, it is necessary to **adjust for all stratification variables** in the analysis (Kahan and Morris, 2012). Stratified randomization forces the distribution of the stratification variables to be exactly equal in both groups; random covariate imbalances as they can occur in simple randomization are ruled out *a priori*. Thus, we have to acknowledge this systematic, non-random factor in our experimental design by including the stratification variables as covariates (Kahan and Morris, 2012).

Lastly, we want to emphasize that ANCOVAs, while widely used, are not the only method to adjust for covariates in an effectiveness analysis. In the primer article, we described that trial estimands should be seen as separate from the concrete analysis model; the latter is simply a statistical vehicle that we used to approximate the former. In a similar way, ANCOVAs are not designed to be a "true" parametric model of all the underlying processes that generated our trial data. We use them to obtain an unbiased estimate of the average treatment effect, and including prognostic covariates increases our precision in doing so. Furthermore, if the covariates are truly prognostic of the outcomes, they also allow us to control for chance imbalances that occurred between the randomized groups.

In the ANCOVA model, covariates are included as simple linear terms. In reality, the covariate-outcome relationship may be much more complex than that. There are methods that adjust for the influence of baseline variables in a more sophisticated way; in section 5.2.3, we will get to know *G-computation*, which is one of them. However, at least for continuous outcomes, it is debatable if these approaches are truly preferable to a "simple" ANCOVA. Simulation studies have shown that ANCOVA retains its desired properties over a wide variety of scenarios (Wang et al., 2019; Egbewale et al., 2014), and is only surpassed by other methods when there are strong treatment-covariate interactions and non-linear relationships (Tackney et al., 2023). Adding to this the fact that ANCOVAs are fairly easy to specify and interpret makes a strong case to use them as a default method in practice.

A different picture emerges if we want to estimate treatment effects on a binary outcome (e.g. response or remission), and we will cover this issue later in this tutorial.

5.1.3 | ANCOVA in Practice

With this theoretical foundation, we now use ANCOVA to examine the treatment effect in our example data. To do this, we first need to make sure that the `mitml`, `purrr` and `miceadds` packages are loaded from the library:

```
library(mitml)
library(purrr)
library(miceadds)
```

Before calculating our treatment effect in the multiply imputed data, let us first have a look at the cases without missings. A **complete case analysis** (CCA) can serve as a helpful sensitivity analysis because it allows to examine the impact that our imputations had on the results. It is

important to stress here that this analysis should only be seen as complementary; typically, it cannot replace the primary effectiveness evaluation conducted in the imputed data. Nevertheless, there are some cases in which a CCA may provide a valid approach even for the main effectiveness evaluation, and not only as a sensitivity analysis. We describe this in greater detail in the infobox at the end of this section.

As described in the previous chapter, ANCOVAs are based on a linear model. Thus, as a first step, we can use the `lm` function in R to fit a linear regression. As part of our call to `lm`, a regression formula has to be specified. The box below describes how this is done using R.

Formula Elements in R

Formulas elements in R follow the basic format `y ~ 1 + x`, where y is the outcome to be predicted, x is the name of the predictor, while 1 symbolizes the intercept (the latter is not mandatory and an intercept will be added automatically if we leave it out). Interactions can be built using an asterisk, for example, `x1 * x2`. Using the `scale` function, we can center and scale continuous predictors directly within the formula, for example, `y ~ 1 + scale(x)`.

In our evaluation, we test the intervention's effect on our primary endpoint (CES-D depressive symptoms at post-test; `cesd.1`) while controlling for the baseline measurement of the same variable (`cesd.0`). To conduct a complete case analysis, we can extract the `data` element from our `imp` object that we generated using the `mice` function (see Chapter 2.2.2). This element contains the *original*, unimputed trial data. We save the results of the `lm` function as `m.lm`. This leads to the following code:

```
m.lm <- lm(cesd.1 ~ 1 + group + scale(cesd.0), data = imp$data)
```

By plugging the results into the `summary` function, we can examine the coefficient estimates of our linear regression model:

```
summary(m.lm)
```

```
## [...]
## Coefficients:
##               Estimate Std. Error t value Pr(>|t|)
## (Intercept)   23.05327    0.60838  37.893  < 2e-16 ***
## group         -6.68026    0.86334  -7.738 8.85e-14 ***
## scale(cesd.0) -0.06087    0.42875  -0.142    0.887
## ---
## Signif. codes:  0 '***' 0.001 '**' 0.01 '*' 0.05 '.' 0.1 ' ' 1
## [...]
```

In the output, we can see that the coefficient of the group variable is $\hat{\beta}_{\text{group}} \approx$-6.68. This means that the post-test CES-D scores in the treatment group (group=1) are approximately 7 points lower in the treatment group receiving the internet-based intervention than in the waitlist control group. The control group mean is captured by the intercept: $\hat{\mu}_{\text{CG}} \approx$ 23.05; the mean in the intervention group can therefore be calculated by subtracting the group effect from this value: $\hat{\mu}_{\text{IG}}$ = 23.05 - 6.68 $\approx$ 16.37.

Looking at the t and corresponding P value of our group coefficient, we see that the scores of both groups do indeed differ significantly when controlling for the baseline depressive symptom severity (p <0.001). To conduct a "real" analysis of variance and corresponding F test, we apply the anova function:

```r
anova(m.lm)
```

```
## [...]
##                 Df  Sum Sq Mean Sq F value    Pr(>F)
## group            1  4361.0  4361.0 59.8613 8.888e-14 ***
## scale(cesd.0)    1     1.5     1.5  0.0202    0.8872
## Residuals      388 28266.4    72.9
## ---
## Signif. codes:  0 '***' 0.001 '**' 0.01 '*' 0.05 '.' 0.1 ' ' 1
```

In the output, we can see that the test of our treatment effect is significant: $F_{1,388}$=59.86, p <0.001.

Naturally, these are only the results based on the complete cases. To ascertain that the treatment can be considered effective as per our trial estimand (see Table X in the primer article), an analysis based on all randomized participants (i.e., the "intention to treat" sample) is indicated. Thus, we will conduct our main effectiveness evaluation in the data that we imputed under the MAR assumption using MICE.

> **Complete Case Analysis: Better Than Its Reputation?**
>
> Apart from the trivial case in which the number of missings is zero or negligible, there are some plausible scenarios in which a CCA may actually *also* be valid for our main effectiveness evaluation. Carpenter and Smuk (2021) describe that there are two special cases for which an analysis of imputed data (under the MAR assumption) does not provide any meaningful benefits: (1) when only values in the outcome variable are missing; or (2) when all patients with missing covariate data also have a missing value in the outcome variable. Since baseline measurements are usually more often recorded than follow-ups, both cases are not unrealistic.
>
> However, this reasoning only holds when we have no *auxiliary variables* in our data that have been observed in patients with missing outcomes, and that are good predictors of missing outcome values. If such auxiliary variables are available, as in our example data set, including them in an imputation model allows to recover additional information that we would not have under a complete case analysis. Many trials include a broad baseline assessment of, e.g., clinical symptoms, demographic variables, risk factors, personality scales, and so forth. If this is the case, an analysis of imputed data sets that incorporate all of this potentially helpful information can be expected to produce better results than a simple complete case analysis.

To begin our analysis of the multiply imputed data, we first convert the `imp` object we created using `mice` into an `mitml.list` object. This type of object contains one imputed data set in each list element and can be used to pool the separate analysis results later on. The `mids2mitml.list` function can be used to do this conversion. We save the results under the name `implist`.

```r
implist <- mids2mitml.list(imp)
```

To confirm that this worked, let us check the class of the newly created object:

```r
class(implist)
```

```
## [1] "mitml.list" "list"
```

We see that, as intended, the `implist` object has the class "`mitml.list`" and "`list`". Please note that our imputation list always needs to have this class so that the pooling works. If the object does not have the desired class, it is possible to run `implist <- as.mitml.list(implist)` to reset it.

To run our ANCOVA regression model in each data set, we can use the `with` function. The first argument specifies the list of imputed data sets, while in the second argument, we provide the same regression formula that we also used in our complete case analysis. Then, use the pipe operator to forward the results to the `testEstimates` function. This function is part of the `mitml` package and allows us to obtain the pooled results based on Rubin's combination rules (see Chapter 4.3):

```
with(implist, lm(cesd.1 ~ 1 + group + cesd.0)) %>%
  testEstimates()
```

```
## [...]
##              Estimate Std.Error  t.value       df  P(>|t|)    RIV    FMI
## (Intercept)    23.453     1.642   14.287 1239.179    0.000  0.248  0.200
## group          -7.124     0.851   -8.376  770.443    0.000  0.337  0.254
## cesd.0         -0.004     0.059   -0.072  841.185    0.943  0.318  0.243
##
## Unadjusted hypothesis test as appropriate in larger samples.
```

In the output, we see that the coefficient of the treatment indicator is $\hat{\beta}_{\text{group}} \approx$-7.12. This effect is significant: $t =$-8.376, $p <$0.001. The output also tells us that the degrees of freedom of this test are $\nu =$770.443. As mentioned before, this somewhat "odd" number is a consequence of the formula that is applied to derive the degrees of freedom in multiply imputed data (see Chapter 4.3.2).

In the `RIV` and `FMI` columns, we are presented with the relative increase in variance due to non-response and fraction of missing information for each coefficient estimate. For our group term, these values are $r_\beta =$0.337 and $\gamma_\beta =$0.254, respectively.

The FMI value is typically a better indicator to gauge the actual "costs" that missingness had on our estimates than, say, the percentage of missing values in a variable (Madley-Dowd et al., 2019). In our example, despite the auxiliary variables in our imputation model, approximately 25.4% of the variation in our estimates is due to the uncertainty of our imputations; the r value tells us that this represents an 33.7% increase in variance. This loss of information and the resulting increase in the variance of our estimate could have been avoided had the data been completely recorded, and parts of it could have been avoided had we been able to build an even better imputation model using further auxiliary variables.

Another thing to note about the output is its last line. This tells us that an unadjusted hypothesis test "as appropriate in larger samples" was conducted. By default, `testEstimates` returns results based on the large-sample formula, which assumes that the complete sample degrees of freedom are infinitely large (see Chapter 4.3). Our example trial included N=546 participants, which means that this method may be roughly appropriate. Nevertheless, let us also check if the results change dramatically if the small sample formula is applied. To do this, we first have to calculate the complete-sample degrees of freedom, ν_{com}:

```
# df.com is defined as sample size minus the number of parameters:
df.com <- nrow(imp$data) - length(coef(m.lm))
```

Then, we can provide this value within our call to `testEstimates`, which will automatically apply the alternative formula:

```
with(implist, lm(cesd.1 ~ 1 + group + cesd.0)) %>%
  testEstimates(df.com = df.com)
```

```
## [...]
##               Estimate Std.Error   t.value       df P(>|t|)    RIV   FMI
## (Intercept)    23.453     1.642    14.287  321.113   0.000  0.248 0.204
## group          -7.124     0.851    -8.376  265.274   0.000  0.337 0.258
## cesd.0         -0.004     0.059    -0.072  275.845   0.943  0.318 0.247
##
## Hypothesis test adjusted for small samples with df=[543]
## complete-data degrees of freedom.
```

We see that the degrees of freedom for our group coefficient are now 265.274, considerably smaller than the 770.443 from before. Apart from that, the results are almost identical.

In the last step, we run the "classic" F-test conducted as part of an ANCOVA. This means that we first have to run an analysis of variance using the anova function, and then extract the F value for our treatment effect. Since we are working with multiply imputed data now, we have to employ the map to perform this operation in all list elements simultaneously. We use the map_dbl function here, which returns a vector of F values from each data set that we save as Fvalues.

```
with(implist, lm(cesd.1 ~ 1 + group + cesd.0)) %>%
  map_dbl(~anova(.)$`F value`[1]) -> Fvalues
```

As described before (see Chapter 4.3), it is not possible to pool test statistics by taking their mean; we have to apply specialized formulas instead. In our case, we can use the micombine.F function in the mitml package to aggregate the F values correctly:

```
micombine.F(Fvalues, df1=1)
```

```
## Combination of Chi Square Statistics for Multiply Imputed Data
## Using 50 Imputed Data Sets
## F(1, 722.34)=69.384     p=0
```

The result is $F_{1,722.34}$ =69.38, which is significant: p <0.001. Thus, we can conclude that the internet-based intervention was effective in this trial.

However, we also want to check the robustness of this effect by conducting a sensitivity analysis in data sets imputed using the **"jump-to-reference"** method. In Chapter 4.2.3, we saved the imputed data in an object we called imp.j2r. This object can also be transformed into a list of data sets using the mids2mitm.list function.

However, the resulting data still needs an additional preparation step. To run the imputation model, we had to convert our data into the long format. Now, to run the same type of analysis as in the main effectiveness evaluation, wide-format data is required. Thus, we use the pivot_wider function to return the imputed data sets back to their original format. Since this has to be done in all sets simultaneously, we plug the operation into the map function. Please note that the pivot_wider function needs to be loaded for the code below to work.

If prepared in the same way as in this example, this code should be usable for all kinds of trial data. It may only be necessary to adapt the values of time accordingly. In our trial, assessments were obtained after '7' and '12' weeks; of course, this may differ from study to study, meaning that the code has to be changed correspondingly.

```
implist.j2r <- mids2mitml.list(imp.j2r)
map(implist.j2r, function(x){
  x %>%
    mutate(time = recode(time, `7` = 1, `12` = 2)) %>%
    pivot_wider(names_from = c(outcome, time),
                names_sep = ".",
                values_from = value)
```

```
}) -> implist.j2r
```

If we check the class of the resulting `implist.j2r` element, we see the issue we mentioned further above: the object class has now changed from `mitml.list` to a simple `list`.

```
class(implist.j2r)
```

```
## [1] "list"
```

This means that the `testEstimates` function we use to pool the parameter estimates will not work. To solve this, we have to reconvert the data using `as.mitml.list`:

```
implist.j2r <- as.mitml.list(implist.j2r)
```

Then, we are ready to apply the same covariate-adjusted linear model we used for the main effectiveness analysis:

```
with(implist.j2r, lm(cesd.1 ~ 1 + group + cesd.0)) %>%
  testEstimates()
```

```
##               Estimate Std.Error   t.value        df P(>|t|)    RIV    FMI
## (Intercept)     22.806     1.708    13.352  1087.150   0.000  0.270  0.214
## group           -4.727     0.844    -5.599  1328.702   0.000  0.238  0.193
## cesd.0           0.009     0.060     0.157  1039.298   0.875  0.277  0.219
##
## Unadjusted hypothesis test as appropriate in larger samples.
```

We see that, like in the main analysis, the treatment coefficient is significant: $t_{1328.702}$ =-5.599, p <0.001. Notably, the imputation uncertainty is lower in the J2R-imputed data, as indicated by an FMI value of γ_β =0.193. However, we also see that the reference-based imputation leads to a more conservative estimate of the treatment effect: $\hat{\beta}_{\text{group}}$ ≈-4.73, compared to the value of -7.12 we found in the data we imputed using `mice`.

As we have learned, jump-to-reference imputation is based on the conservative (but not impossible) assumption that all patients behave exactly like the control group once they are lost to follow-up. Given that the number of missings at post-test is substantial, we see that such an assumption almost halves the treatment estimate we obtain when assuming MAR. However, we also see that this effect is still significant, which provides further evidence that the effect detected in the main analysis is indeed robust. In practice, we would also conduct an F-test for the J2R-imputed data using the same code we used for the main analysis. We omit this step here.

One-Sided Testing: A Good Idea?

In the analyses above, we used two-sided tests to confirm that our treatment was effective. These two-sided tests are the default returned by most implementations in R. A two-sided test is based on the null hypothesis of no effect, $\mu_1 - \mu_2 = 0$, which allows testing for both positive treatment effects (meaning that the intervention was beneficial) as well as negative effects.

One could argue that in RCTs, our hypotheses are not really two-sided. We want to know if the evaluated treatment is *better* than the control group, not if it is better or worse. This fits better with a one-sided test, which also happens to give us greater power than two-sided testing. Some have argued along these lines to

propose that one-sided testing is preferable, at least in many contexts (Knottnerus and Bouter, 2001; Owen, 2007).

While there is some controversy surrounding the question of one- vs. two-sided testing, the general consensus in the medical literature seems to be that two-sided testing should be used as the default approach (Lydersen, 2021). There are good reasons for this: if a one-sided test is conducted, we cannot make any statements on potential negative effects that the intervention may have had, even if our data (e.g. the mean difference between two groups) indicates that this is a possibility (Moyé and Tita, 2002). Effects in the opposite direction may not be likely, but they are possible; mental health treatments can indeed be harmful (McKay and Jensen-Doss, 2021; Lilienfeld, 2007; Hengartner, 2017), and it is difficult, if not impossible, to rule out *a priori* that this will not be the case in our trial.

Obviously, no matter if a one- or two-sided testing strategy is used, this must be *pre-specified* in the analysis plan. Otherwise, we may be tempted to switch from a two- to a one-sided testing strategy because our treatment effect would not be significant otherwise. It goes without saying that this would constitute a highly questionable research practice.

5.1.4 | Calculating the Standardized Mean Difference

In the previous chapter, we demonstrated the effectiveness of our intervention. Now, we want to quantify this effect using a measure that is interpretable beyond the specific context of this trial. This is typically achieved by calculating *effect sizes*.

The term "effect size" is not clearly defined. Some people think of effect sizes as standardized mean differences (SMDs), also known as Cohen's d, while others prefer a broader definition. The "narrow" definition may be difficult to uphold, since correlations, odds ratios, Z values, etc., can also express the direction and strength of an effect, and can even be partly transformed into each other (Harrer et al., 2021, chap. 3.1).

Within RCT analyses, effect sizes are used to quantify the size of the treatment effect, and make it comparable to similar studies. For continuous outcomes, the most commonly used effect size measure is the standardized mean difference, the true value θ of which is defined as:

$$\theta = \frac{\mu_{\text{IG}} - \mu_{\text{CG}}}{\sigma} \tag{31}$$

where μ_{IG} and μ_{CG} are the true means of the intervention and control group, and σ is the true standard deviation of the outcome in the population. Notably, none of these three quantities can be directly observed; we can only estimate them from our trial data. This is typically done by replacing the μ's with the intervention and control group means $\bar{Y}_{\text{IG}}$ and $\bar{Y}_{\text{CG}}$, and σ with the pooled standard deviation s_{pooled} of our outcome Y in both groups. This leads to the "classic" formula for what is commonly known as *Cohen's* d:

$$d = \frac{\bar{Y}_{\text{IG}} - \bar{Y}_{\text{CG}}}{s_{\text{pooled}}} = \frac{\bar{Y}_{\text{IG}} - \bar{Y}_{\text{CG}}}{\sqrt{\dfrac{(n_{\text{IG}}-1)s^2_{\text{IG}} + (n_{\text{CG}}-1)s^2_{\text{CG}}}{(n_{\text{IG}}-1)+(n_{\text{CG}}-1)}}} \tag{32}$$

Notably, s_{pooled} can also be replaced by other, external estimates of σ if they are available. This may be particularly helpful when our trial sample is small, or when there are other reasons to believe that s_{pooled} is a bad estimator of the population standard deviation. Sometimes, the control group standard deviation alone is also used to standardize the mean difference, for example when there is more than one intervention group. This, however, leads to a different effect size metric

known as *Glass' Δ*.

Equation 32 above is typically used to calculate the between-group Cohen's d in clinical trials and meta-analyses. In practice, a greater challenge is the construction of valid confidence intervals around this point estimate. For many effect sizes, including d, there are closed-form equations to calculate the sampling variance, which is required to determine the confidence intervals. However, if we use these formulas for effect sizes derived from multiply imputed data, we neglect that our estimates are also additionally affected by imputation uncertainty. This would lead to anti-conservative results; that is, confidence intervals that are too narrow.

A more elegant way to calculate effect sizes is to derive them from the linear covariate-adjusted model we used to estimate the treatment effect. In our model, we estimate β_{treat}, which can be interpreted as the mean difference in outcomes between the two groups: $\hat{\beta}_{\text{treat}} = \widehat{\text{ATE}} = \hat{\mu}_{\text{IG}} - \hat{\mu}_{\text{CG}}$. Covariate adjustment within the model ideally means that our estimate of this treatment effect becomes more precise and that we control for random imbalances in prognostically relevant baseline variables. Using Rubin's rules, we can also obtain valid confidence intervals around our parameter estimate that incorporate our imputation uncertainty.

Therefore, we can calculate an estimate of the true effect size, as well as its lower and upper confidence limit, if we standardize $\hat{\beta}_{\text{treat}}$ by the pooled standard deviation:

$$\hat{\theta} = \hat{\beta}_{\text{treat}} / s_{\text{pooled}} \tag{33}$$

$$\hat{\theta}_{\text{lower}} = (\hat{\beta}_{\text{treat}} - t_{\nu_{(\text{MI})},0.975} \times \text{S.E.}_{\hat{\beta}_{\text{treat}}}) / s_{\text{pooled}} \tag{34}$$

$$\hat{\theta}_{\text{upper}} = (\hat{\beta}_{\text{treat}} + t_{\nu_{(\text{MI})},0.975} \times \text{S.E.}_{\hat{\beta}_{\text{treat}}}) / s_{\text{pooled}} \tag{35}$$

Time to illustrate this in practice. First, we need to calculate s_{pooled}. We can do this by extracting the intervention and control group standard deviation from each imputed data set in `implist`, and then taking the mean value. Then, we use the formula in equation 32 to obtain the pooled value.

```
# Calculate mean SD for both groups across imputed data sets
implist %>%
  map_dbl(~ filter(., group==1) %>% pull(cesd.1) %>% sd()) %>%
  mean() -> s_ig

implist %>%
  map_dbl(~ filter(., group==0) %>% pull(cesd.1) %>% sd()) %>%
  mean() -> s_cg

# Define sample size in both groups
n_ig <- n_cg <- 273

# Calculate pooled SD
sqrt(((n_ig-1)*s_ig^2 + (n_cg-1)*s_cg^2)/
       (n_ig+n_cg-2))
```

```
## [1] 8.585904
```

We see that $s_{\text{pooled}} \approx 8.586$. We can now use this value to standardize the mean difference estimated by the coefficient of `group`. A convenient way to do this is to include the "`AsIs`" function `I()` directly in the model formula. Specifically, we are writing `I(cesd.1/8.586)` in the outcome part of the formula to indicate that the coefficients should be divided by the pooled standard deviation we just calculated. For the main effectiveness analysis, this leads to the following code:

```
(m.es <- with(implist, lm(I(cesd.1/8.586) ~ 1 + group + cesd.0)) %>%
  testEstimates())
```

```
## [...]
##             Estimate Std.Error t.value       df P(>|t|)   RIV   FMI
## (Intercept)    2.732     0.191  14.287 1239.179   0.000 0.248 0.200
## group         -0.830     0.099  -8.376  770.443   0.000 0.337 0.254
## cesd.0        -0.000     0.007  -0.072  841.185   0.943 0.318 0.243
## [...]
```

In the output, we see that the coefficient of our group term is now -0.83, which can be directly interpreted as the value of Cohen's d. To create a confidence interval around the effect, we can plug the model into the `confint` function:

```
confint(m.es)
```

```
##                  2.5 %      97.5 %
## (Intercept)  2.35648956  3.10666882
## group       -1.02413018 -0.63521373
## cesd.0      -0.01404827  0.01305376
```

Thus, we see that the effect size, based on the main effectiveness analysis, is d=-0.83, with the 95% confidence interval ranging from -0.64 to -1.02.

Next, we use the same approach to calculate the effect size for our complete case analysis:

```
(m.es.cca <- lm(I(cesd.1/8.586) ~ 1 + group + cesd.0,
               data = imp$data))
confint(m.es.cca)
```

```
## [...]
## Coefficients:
## (Intercept)        group       cesd.0
##   2.7114437   -0.7780405   -0.0009943
##
##                  2.5 %      97.5 %
## (Intercept)  2.32328249  3.09960484
## group       -0.97573543 -0.58034567
## cesd.0      -0.01476584  0.01277715
```

This results in a roughly comparable estimate of d=-0.78 (95%CI: -0.58 to -0.98). Lastly, we can also calculate the effect size based on our reference-based imputation analysis:

```
(m.es.j2r <-
    with(implist.j2r, lm(I(cesd.1/8.586) ~ 1 + group + cesd.0)) %>%
    testEstimates())
confint(m.es.j2r)
```

```
## [...]
##             Estimate Std.Error t.value       df P(>|t|)   RIV   FMI
## (Intercept)    2.656     0.199  13.352 1087.150   0.000 0.270 0.214
## group         -0.551     0.098  -5.599 1328.702   0.000 0.238 0.193
## cesd.0         0.001     0.007   0.157 1039.298   0.875 0.277 0.219
## [...]
##                  2.5 %      97.5 %
## (Intercept)  2.26582833  3.04649726
## group       -0.74340278 -0.35762665
## cesd.0      -0.01265622  0.01486229
```

As we discussed before, the effect here is considerably lower, with d = -0.55 (95%CI: -0.36 to -0.74).

Interpretation of Cohen's *d*

Values of d are often interpreted in reference to the "rule of thumb" by Cohen (1988), which states that d=0.2, 0.5, and 0.8 represent small, moderate, and large effects, respectively. A better approach is to compare the effect sizes in our trial to other, similar studies; or to established, field-specific reference values.

For depression treatments, for example, it has been found that the minimally important difference that patients would still recognize as beneficial is d = -0.24 (Cuijpers et al., 2014). All the effect estimates we obtained in our example analyses (d = -0.55 to -0.83) are above this threshold, suggesting that the effects of our intervention can be considered clinically meaningful.

5.2 | Binary Endpoints

In our example trial, the primary endpoint was depressive symptom severity, which can be intuitively expressed on a continuous scale. In other trials, the primary endpoint may be *binary*, that is, based on a condition that is either met or not met. A natural example are trials in which the primary outcome is patients' diagnostic status at some follow-up assessment (especially in trials in which all patients fulfilled the diagnostic criteria at baseline).

We may also be interested in how a treatment affects individuals' diagnostic status *over time*. This can be done by comparing the incidence rates of different groups; or, if many assessments over time are available, via a *survival analysis*. The latter is frequently used in prevention trials to examine if some treatment reduces the hazard of developing a mental disorder within the examined time frame. Survival analysis is a multi-faceted topic that we do cover here. Luckily, however, there are helpful resources that detail how such analyses can be implemented using R (Moore, 2016).

In other cases, binary outcomes are derived from endpoints that were originally measured on a continuous scale. This is typically done to transform outcomes into results that can be more easily interpreted by clinicians and laypeople. We achieve this by determining the number of patients who experienced some form of reliable or clinically significant change during the study period, and if these numbers differ significantly between groups. In the following section, we will discuss ways to determine such binary metrics of change and showcase how to calculate the most commonly used one. This will also provide us with a binary endpoint that we will analyze afterwards.

Before we start, we want to issue a cautionary note concerning the "derived" binary outcome measures that we will be calculating next. From a statistical perspective, pigeonholing a continuous outcome into simple yes/no metrics is a questionable practice that is known as *dichotomania* among trial statisticians (Senn, 2005). Dichotomizing continuous endpoints means that we actually lose a lot of outcome information, and it typically forces us to put patients into completely distinct categories, even if they hardly differ in their outcomes. In the context of our example trial, the following analyses should only be seen as supplementary to the main evaluation we conducted in the previous chapter.

5.2.1 | Reliable Change & Minimal Clinically Important Differences

A widely used method to determine which patients improved during the study period is the **reliable change index**, or RCI (Jacobson and Truax, 1992). This metric is particularly common in clinical psychology and often found in psychotherapy trials. The reasoning behind the RCI is that, if we find a difference between some person i's score at baseline and some later assessment, it is unclear if this represents real symptom change, or simply some random variation attributable to measurement error. Thus, we need a measure that allows us to gauge if some pre-post difference $Y_{1,i} - Y_{0,i}$ in the

outcome Y truly represents *reliable* change. The RCI allows us to do this by standardizing the "raw" score difference by an estimated standard error of measurement, s_{diff}:

$$\text{RCI} = \frac{Y_{1,i} - Y_{0,i}}{s_{\text{diff}}} \quad \text{with} \quad s_{\text{diff}} = \sqrt{2(s_0\sqrt{1-r_{YY}})^2}. \tag{36}$$

In this formula, s_0 is the baseline standard deviation of Y, and r_{YY} is the **test-retest reliability** of our instrument. This reliability coefficient has to be determined from previous literature. Typically, estimates of validation studies in similar populations are used for this.

The calculated RCI values of each participant can then be employed to identify those who have achieved reliable change. Similar to Z-scores, we assume that the RCI values follow a standard normal distribution with a critical value of 1.96. Thus, if RCI $\leq$ -1.96, we conclude that a person reliably improved (assuming lower values indicate better outcomes). If RCI $\geq$ 1.96, a person experienced reliable deterioration. All difference scores in between are counted as unchanged:

$$\text{if} \begin{cases} |\text{RCI}| \geq \Phi^{-1}(0.975) \Rightarrow |\text{RCI}| \geq 1.96 : \text{reliable change.} \\ |\text{RCI}| < \Phi^{-1}(0.975) \Rightarrow |\text{RCI}| < 1.96 : \underline{\text{no}} \text{ reliable change.} \end{cases} \tag{37}$$

We have implemented equation 36 above into a function that can be used to calculate RCIs using R. This function is not pre-installed, so we have to let R "learn" it by copying and pasting the code below into the console, and then hitting Enter.

```
rci <- function(y0, y1, ryy){
  diff = y1-y0
  sdiff = sqrt(2*((sd(y0)*sqrt(1-ryy))^2))
  return(diff/sdiff)
}
```

As we have mentioned, the RCI formula requires us to provide an estimate of the test-retest reliability of our instrument. In our example, we use a value of $r_{YY} = 0.85$, which has been calculated in a previous study that validated the CES-D.

```
ryy <- 0.85
```

Before we use the `rci` function in practice, let us first determine the value of s_{diff}. Using `map`, we calculate the value in each imputed data set and then take the mean. Please note that this is not technically necessary in our example trial, in which all baseline values are recorded; but the code below may come in handy if this is not the case. We save the result as `s_diff`.

```
 implist %>%
  map_dbl(~sqrt(2*((sd(.$cesd.0)*sqrt(1-ryy))^2))) %>%
  mean() -> s_diff

s_diff
```

```
## [1] 3.904825
```

We see that in our trial, assuming r_{YY} = 0.85, the standard error of measurement is s_{diff} = 3.905. Thus, we conclude that a reliable symptom change occurred if the change between baseline and post-test is greater than 1.96 × 3.905 = 7.654 points.

Next, we calculate for each of our patients, if they experienced reliable improvement or deterioration based on the RCI. We again have to do this in all imputed data sets, for which we use `map`

again. In the supplied function, we use the `rci` function to create a new variable in which the exact RCI value of each person is stored. Then, we use the `ifelse` function to determine if the RCI value is above 1.96 or below -1.96. This results in binary indicator variables (i.e., a variable that is either 1 or `0`) for reliable improvement (`ri`) and deterioration (`rd`).

```
implist %>%
  map(function(x){

    # Calculate RCI for each person
    x$rci = with(x, rci(y0 = cesd.0, y1 = cesd.1, ryy = ryy))

    # Create binary indicator for
    # reliable improvement & deterioration
    x$ri = ifelse(x$rci <= -1.96, 1, 0)
    x$rd = ifelse(x$rci >= 1.96, 1, 0)
    x

}) -> implist
```

Each element in `implist` now contains three new variables containing the RCI value, as well as the reliable improvement and deterioration indicator for each patient. For now, the outcome we are most interested in is reliable improvement. Thus, we now summarize the number of individuals with reliable improvement in both treatment groups and aggregate the values across all imputation sets. This can be achieved using the code below. We save the result as `table.ri`:

```
# Get the number of imputation sets
m <- length(implist)

# Generate a reliable improvement counts table
# Aggregate the values across all data sets.
implist %>%
  map(~ with(., table(group, ri))) %>%
  {Reduce(`+`, .)/m} %>%
  as.matrix() %>%
  round() -> table.ri

table.ri
```

```
##       ri
## group   0   1
##     0 173 100
##     1 107 166
```

This leaves us with a 2×2 table, with the rows indicating the group (`0` = control, 1 = intervention), and the columns indicating the reliable improvement status (`0` = not improved, 1 = reliably improved). We see that considerably more individuals in the intervention group (n =166) experienced a reliable improvement than in the control group (n=107), although we do not know yet if this is statistically significant.

Based on these cell counts, we can also derive another metric that is commonly calculated in trials: the **number needed to treat**, or NNT. This treatment effect measure is often used because it is easier to understand by clinicians and other stakeholders[2]. NNTs indicate, based on the present

[2]This idea of NNTs being particularly "intuitive" measures of treatment effects is not uncontroversial. Some have argued that NNTs are actually often misunderstood and falsely calculated in the medical literature (Christensen and Kristiansen, 2006; Mendes et al., 2017).

evidence, the number of individuals that needs to be treated with the intervention to cause one additional positive event in the studied population. In our example, this would be one additional case of reliable improvement. Depending on the nature of the outcome, NNTs can also be interpreted as the number of patients we need to treat to *prevent* on additional *negative* outcome (e.g. symptom deterioration).

NNTs not only depend on the treatment effect but also on how common or uncommon an event is *in general*. Even if a treatment is very effective, if the event under study is extremely rare, this still means that we have to treat a lot of patients to cause or prevent one additional case, given that most people would not have experienced the event anyway. Naturally, this leads to a much higher NNT than when the event is very common.

NNTs can be calculated as the inverse of the *absolute risk reduction*, or ARR. The ARR itself is determined by subtracting the event rate in the control group $p_{e_{\text{control}}}$ from the event rate in the intervention group $p_{e_{\text{treat}}}$:

$$\begin{aligned} \text{NNT} &= (p_{e_{\text{treat}}} - p_{e_{\text{control}}})^{-1} = \text{ARR}^{-1} \\ p_{e_{\text{treat}}} &= \frac{n_{e_{\text{treat}}}}{n_{\text{treat}}} \quad \text{and} \quad p_{e_{\text{control}}} = \frac{n_{e_{\text{control}}}}{n_{\text{control}}}. \end{aligned} \tag{38}$$

Based on our 2×2 table, we can now calculate the NNT for reliable improvement.

```
# Calculate sample size in both groups
n.ig <- sum(table.ri[2,])
n.cg <- sum(table.ri[1,])

# Calculate proportion of reliable improvement cases
p.ig <- table.ri[2,2]/n.ig
p.cg <- table.ri[1,2]/n.cg

# Calculate NNT (inverse risk reduction)
(p.ig - p.cg)^-1
```

```
## [1] 4.136364
```

We see that the result is NNT $\approx$ 4.14. Rounding this value up, we can say that five patients need to receive the Internet-based intervention to generate one additional case of reliable improvement. While this may seem high at first glance, this is actually quite a low value for a mental health intervention. In preventive contexts, it is not unusual to find NNTs of 20 or higher.

Lastly, we can also visualize the reliable change patterns in our data. For simplicity, we will only plot the data of one imputation set here, and we will choose the first one. To produce a plot for each group, we generate two separate data sets `dat.plt.ig` and `dat.plt.cg`.

```
dat.plt.ig <- implist[[1]] %>% filter(group == 1)
dat.plt.cg <- implist[[1]] %>% filter(group == 0)
```

Making plots in R can be tricky, especially for beginners. However, it is not crucial to understand everything about the code we use below. The syntax can also be adapted by changing the name of the `cesd.0` and `cesd.1` variable to the relevant outcome measured at baseline and post-test in your particular trial. The same is also true for the axis labels provided in the `xlab` and `ylab` argument.

```
# We require the scales package to be loaded.
library(scales)

# intervention group plot
```

```
plot(dat.plt.ig$cesd.0, dat.plt.ig$cesd.1,
     xlim = c(0,50), ylim = c(0,50),
     xlab = "CES-D (Baseline)", ylab = "CES-D (Post-Test)",
     pch = 16, col = alpha("black", 0.3),
     main = "Intervention Group")
abline(coef = c(0,1), col = "blue")
polygon(c(0,0,50,50), c(0-s_diff*qnorm(.975),0+s_diff*qnorm(.975),
                        50+s_diff*qnorm(.975),50-s_diff*qnorm(.975)),
        col = alpha("lightblue", 0.3), border = NA)
text(50, 0, "Improvement", pos=2, col="darkgreen")
text(0, 50, "Deterioration", pos=4, col="darkred")


# control group plot
plot(dat.plt.cg$cesd.0, dat.plt.cg$cesd.1,
     xlim = c(0,50), ylim = c(0,50),
     xlab = "CES-D (Baseline)", ylab = "CES-D (Post-Test)",
     pch = 16, col = alpha("black", 0.3),
     main = "Control Group")
abline(coef = c(0,1), col = "blue")
polygon(c(0,0,50,50), c(0-s_diff*qnorm(.975),0+s_diff*qnorm(.975),
                        50+s_diff*qnorm(.975),50-s_diff*qnorm(.975)),
        col = alpha("lightblue", 0.3), border = NA)
text(50, 0, "Improvement", pos=2, col="darkgreen")
text(0, 50, "Deterioration", pos=4, col="darkred")
```

These are the resulting plots:

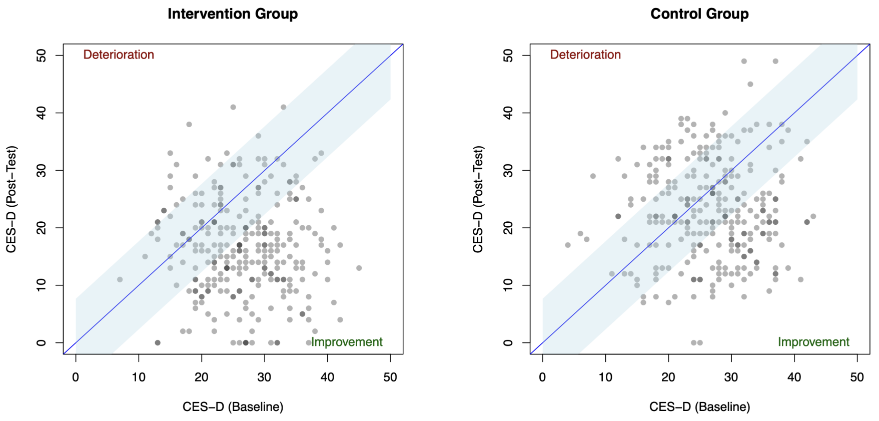

The graphs show, for each participant, the baseline CES-D values on the *x*-axis, and the post-test values on the *y*-axis. When there is no difference between the pre- and post-test scores, individual points will end up exactly on the diagonal. The light blue bands represent score changes that we are willing to attribute to measurement error, meaning that all points in this region are categorized as unchanged. Beyond the blue band, points in the lower triangle represent cases with reliable improvement, and points in the upper triangle are those with reliable deterioration. As we can see, the number of reliably improved patients in the lower triangle is considerably higher in the intervention compared to the control group.

The Perils of Standardization: Some Problems with the RCI

Although widely used in mental health research, the RCI has certain limitations that we should acknowledge (Wise, 2004; McAleavey, 2021). If we go back to the formula

36, we see that, besides the raw change score, the RCI depends on two variables: the reliability coefficient r_{YY}, and the in-sample standard deviation at baseline s_0.

Selecting a good value of r_{YY} can be challenging because it has to be determined from previous literature. It is often unclear if the validation sample is representative of the type of patients in our trial. It also leaves us with many *researcher degrees of freedom*: the size of s_{diff} depends heavily on the reliability coefficient, so we may be tempted to always select the highest value we find in the literature to make sure that the number of "reliably" improved individuals is as large as possible. Furthermore, it is important to remember that only the test-retest reliability can be used to calculate RCIs. Often, measures like Cronbach's α are used instead, which can lead to wildly incorrect results.

This ambiguity also means that the comparability of reliable improvement rates across different trials may be limited. This is further exacerbated by the second variable, s_0, which can vary considerably between trials, even when the same instrument was used in similar populations. We use the RCI to determine if a specific patient in our trial has reliably improved or deteriorated. It is odd that this individual response measure depends on the variability of other people's scores in the trial, but this is exactly what happens if we use the RCI.

Imagine the following scenario: we have two patients with exactly the same pre- and post-test scores, but one happened to be part of a trial with few inclusion criteria and large s_0, while the other was included in a trial with very strict inclusion criteria, and thus a very low baseline standard deviation. Both patients experience exactly the same symptom decrease, but in the first trial we would likely regard this as "unchanged" (because s_0 is large) whereas, in the second trial, we would consider this change to be a reliable improvement. From a patient perspective, it is illogical that my improvement may depend on the specific trial is was lucky or unlucky enough to get assigned to.

The RCI performs a type of "standardization", but since this depends on quantities calculated within our sample, our results may often be less comparable than we think they are. In a famous paper, Cohen (1994) describes that this issue also pertains to all kinds of standardized effect measures, including Cohen's d.

We have now determined the reliable improvement and deterioration status for each of our patients. The `ri` variable we calculated in this chapter will be reused in the next section, where we describe how to run an effectiveness analysis based on binary outcome data.

It is important to stress that the RCI is by no means the only measure we can calculate from continuous outcome data. There is a completely different strain of metrics revolving around the concept of "clinically significant" (CS) change, which is measured by falling below a practically or clinically relevant cut-off. Such cut-offs are available for many commonly used symptom questionnaires in mental health research, for example, the PHQ-9 (Manea et al., 2012), GAD-7 (Spitzer et al., 2006), or CES-D (Vilagut et al., 2016).

The problem with these cut-offs is that they do not account for baseline symptom severity. Say, for example, that a score of $Y \leq 10$ on an instrument represents symptoms that are not clinically relevant anymore. If a person changes from $Y_0 = 11$ to $Y_1 = 10$, we would hardly consider this an important change, but the person would still be considered to have experienced clinically significant change. Thus, the RCI and CS measures are often combined to obtain rates of *reliable and clinically significant improvement* (RCSI), where patients have to show both reliable improvement *and* score below a certain cut-off.

We may also calculate improvement rates using *minimally important differences*, or MIDs. Typically, MIDs are minimum raw change scores for a specific outcome that a patient needs to surpass for it to be considered meaningful from a patient's perspective. A plethora of MIDs have been developed for various mental health questionnaires, but such validation studies unfortunately often differ in their examined populations, methodology, and overall quality (Devji et al., 2021). If MIDs are to be used, it is advisable to search for reference values obtained using so-called *anchor-based* methods. These

approaches determine minimally important changes based on an external "anchor"; for example, a clinician or self-report assessment if a patient feels "slightly better" after the treatment.

Yet another response metric is the *effective dose 50*, or ED50 (Bauer-Staeb et al., 2021), which encodes a 50% probability of patients "feeling better" given their initial baseline severity. This measure is commonly used in pharmacological trials. It is less common in behavioral mental health studies, although it is also applicable in these contexts. In R, this approach can be implemented using the `ed50` package (Gan et al., 2019).

5.2.2 | Generalized Linear Models

In the previous chapter, we learned how to evaluate treatment effects using ANCOVA (i.e., a covariate-adjusted linear model), which is suitable when our primary endpoint is continuous. However, there are many realistic scenarios in which we cannot assume that our outcome is continuous, and approximately follows a normal distribution. A few examples are provided in the graphic below.

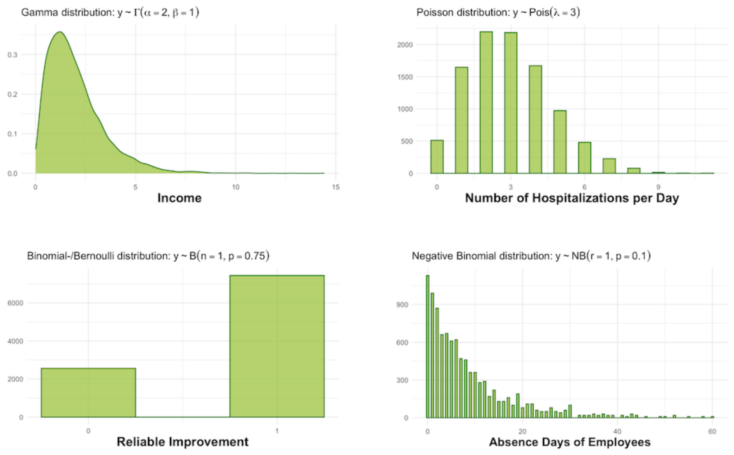

Reliable improvement, which we calculated in the previous section, is a prominent example. We assume that improvement is a binary outcome, where the proportion of improvement and non-improvement is determined by a Bernoulli distribution with an unknown parameter p. To test our treatment effect, we need a linear model that allows to accommodate this alternate distributional assumption for our endpoint. This is where *generalized linear models* (GLMs; Dunn and Smyth, 2018, chap. 5) provide a solution.

As it says in the name, GLMs are a generalization of the linear model to the entire exponential dispersion model (EDM) family. A few distributions in this family are displayed in the figure above. In practice, GLMs are frequently used to model count and event data, or other dichotomous outcomes. Computationally, there is a difference between the "standard" linear model and GLMs: the former can be derived directly with ordinary least squares (OLS); that is, using basic matrix algebra. For GLMs, coefficients need to be determined iteratively using maximum likelihood estimation (MLE). This means that the latter are more computationally intensive. Yet, this is typically barely noticeable in R in terms of computation time.

GLMs consist of two components: a linear predictor η_i, and a **link function** $g(\cdot)$:

$$\eta_i = (o_i) + \alpha + \mathbf{X}_i^\top \beta. \tag{39}$$

$$g(\mu_i) = \eta_i. \tag{40}$$

Where $\mathbf{X}$ stands for all the variables we want to include in our linear model, in our case the treatment indicator and covariates. In this context, the inverse of the link function "translates" values of the linear predictor back into the format of the outcome variable: $g^{-1}(\eta_i) = \mu_i$; $\mu = E(Y_i)$.

The o_i term in equation 39 above represents an *offset*, which is often used in Poisson and negative binomial models. Offsets are *a priori* known values for which no parameter estimation is necessary. They are frequently used to model the time of exposure E in the analysis of certain clinically relevant events. Say, for example, that we conducted a trial in aftercare in which we recorded if patients relapsed, but that the follow-up times differed slightly between patients. In this case, patients differed in the time in which they were *exposed* to the risk of relapse, and this exposure time E should be accounted for in our model.

Data like this are typically fitted using a Poisson model in which E serves as the offset. This offset is used to "standardize" the resulting response by the exposure time:

$$
\begin{aligned}
g(\mu) &= \overbrace{\log_e(\mu)}^{\text{link function}} = \eta \\
g(\mu) &= \log_e\left(\frac{\mu}{E}\right) = \eta \quad \text{(with offset)} \\
&= \log_e(\mu) = \eta + \log_e(E)
\end{aligned}
\tag{41}
$$

It is not essential to understand everything about the formula above. The crucial message is that offsets are a helpful way to incorporate the effect of time into an analysis model. In R, we could fit a Poisson model with exposure time E, treatment indicator `trt`, covariate x and outcome y like this:

```
glm(y ~ trt + x + offset(E), data = data, link = poisson)
```

The code above already displays the core arguments we need when fitting a GLM using R. In the first argument, we specify the model formula, as we have for our ANCOVA model. Here, we additionally use the `offset` function to include an offset term. The special argument in the `glm` function is `link`. Here, we specify the link function that corresponds with the assumed distribution of our outcome variable.

In Table 2 below, we summarize some of the main link functions used in GLMs, and how they can be implemented in R. In Chapter 5.1.3, we already described that the group coefficient in linear models can be elegantly expressed as the estimated mean difference in our trial and that we can derive the treatment effect size expressed as Cohen's d from this value. GLMs follow a similar logic if we use the *antilog* (i.e., the reverse logarithm) on the estimated treatment coefficient β_{treat}. In R, the `exp` function can be used to take the antilog.

The table shows the effect size metric that the resulting value can be interpreted as, for each respective link function. We see, for example, that β_{treat} gives us the odds ratio (OR) for models with a binomial logit-link (more commonly known as logistic regression). For Poisson models, taking the antilog leads to an estimate of the incidence rate ratio (IRR).

Table 2: Overview of link functions in GLMs.

Distribution	Link	Link Function	Support (y)	exp(β_{treat})	`family`
Normal	Identity	μ	$\mathbb{R}$	$\log_e$(MD)	`gaussian(„identity")`
Binomial	Logit	$\log_e\left(\frac{\mu}{1-\mu}\right)$	$\frac{0,1,\ldots,m}{m}$	OR	`binomial(„logit")`
	Log	$\log_e(\mu)$	$\frac{0,1,\ldots,m}{m}$	RR	`binomial(„log")`
	Complemen. Log-Log	$\log_e\left[-\log_e(1-\mu)\right]$	$\frac{0,1,\ldots,m}{m}$	HR	`binomial(„cloglog")`
Negative Binomial	Log	$\log_e\left(\frac{\mu}{\mu+k}\right)$	$\mathbb{N}_0$	(I)RR	log (in `MASS::glm.nb`)
Poisson	Log	$\log_e(\mu)$	$\mathbb{N}_0$	(I)RR	`poisson(„log")`
Gamma	Inverse	$-\mu^{-1}$	$\mathbb{R}_+$	-	`gamma(„inverse")`

As a next step, we will use a GLM to test the effect of our Internet-based intervention on reliable improvement. For now, let us employ the same formula we used in the ANCOVA model in Chapter 5.1.3, where the outcome is regressed on the treatment indicator (`treat`) while adjusting

for baseline symptom severity (`cesd.0`) as a covariate. Naturally, the outcome in our formula is now different: we want to examine effects on reliable improvement as captured by the variable `ri` in our data, which can either be 1 (improved) or `0` (not improved).

With our outcome `ri` being binary and measured at the same time point, the binomial logit is a natural choice for our link function. This means that we can fit a logistic regression model to our data. Logistic regression is the most widely used method for binary outcome data because it has desirable computational properties. As we learned, GLMs need to be fitted using an iterative process, and logistic regression models usually arrive at the optimal solution rapidly and without errors. This is one of the great advantages of logistic regression compared to, say, log-binomial models, where the computation is considerably more challenging, and often fails (Williamson et al., 2013).

Thus, in our call to `glm`, we set the `link` argument to `binomial("logit")`. As before, we use the `with` and `testEstimates` functions in `mitml` to obtain a pooled result from the multiply imputed data. The final code looks like this:

```
implist <- as.mitml.list(implist)
with(implist, glm(ri ~ group + scale(cesd.0),
         family = binomial("logit"))) %>%
   testEstimates() -> m.glm; m.glm
```

Let us have a look at the results:

```
##                 Estimate Std.Error t.value      df P(>|t|)   RIV   FMI
## (Intercept)       -0.764     0.173  -4.413 799.167   0.000 0.329 0.249
## group              1.360     0.246   5.526 840.589   0.000 0.318 0.243
## scale(cesd.0)      1.354     0.157   8.636 618.440   0.000 0.392 0.284
## [...]
```

The structure of the output is identical to the one of our ANCOVA model in Chapter 5.1.3. We are again presented with the relative increase in variance, fraction of missing information, and of course with the estimated coefficients and their significance. We see that the group term is significant ($p < 0.001$), meaning that our treatment seems to have an effect on reliable improvement at post-test.

To interpret the effect, we can go back to Table 2, which tells us that taking the antilog of our treatment effect gives us a model-based estimate of the odds ratio. We can calculate it like this:

```
exp(m.glm$estimates["group", "Estimate"])
```

```
## [1] 3.896621
```

Thus, we can conclude that the treatment effect is equivalent to OR ≈ 3.9.

In Chapters 5.1.1 and 5.1.2, we discussed the benefits of covariate adjustment in greater detail. As we learned, in RCTs, the unadjusted (mean) difference between both groups is already an unbiased estimator of the average treatment effect; the main reason to adjust for covariates is to increase our statistical power, not necessarily to "correct" our effect estimate. Put differently, when the outcome is continuous, both the unadjusted mean difference $\bar{Y}_{\text{IG}} - \bar{Y}_{\text{IG}}$, and the estimated treatment coefficient $\hat{\beta}_{\text{treat}}$ in our ANCOVA model try to estimate the same "true" treatment effect $\mu_{\text{IG}} - \mu_{\text{CG}}$. The latter just typically happens to have more statistical power in doing so.

Unfortunately, this logic does **not generalize to logistic regression models**. We can inspect this by running our model again, but this time, we remove the baseline covariate. This leads to an *unadjusted* model:

```
with(implist, glm(ri ~ group, family = binomial("logit"))) %>%
```

```
    testEstimates() -> m.glm.ua
  exp(m.glm.ua$estimates["group", "Estimate"])
```

```
## [1] 2.664535
```

We see that this suddenly provides us with a considerably lower treatment effect of $\text{OR} \approx 2.66$. This result is not caused by some gross baseline imbalance in CES-D scores that our first model would control for, nor is this a chance finding: when we adjust for a prognostically relevant covariate in logistic regression, the confidence intervals do not tighten up, but the estimated odds ratio increases instead (Jiang et al., 2017; Groenwold et al., 2011).

In the primer article we mentioned that, arguably, covariate-adjusted models lead to a *personalized* interpretation of the treatment effect: it is the expected difference between two individuals with the same covariate values, where one receives the treatment, and the other does not. While we should not overstretch this analogy, it does point to an important insight: while not controlling for baseline covariates leads to a marginal, or *unconditional* treatment effect, covariate-adjusted models produce *conditional* treatment effects (viz., conditional on covariates). It just happens that, with continuous outcomes, this does not change the estimand: both are estimates of the average treatment effect that we as trialists are typically interested in.

This is not the case with logistic regression, where covariate-adjusted models capture a genuinely different conditional effect size. The estimate is not *wrong*, it simply does not approximate the estimand we are actually interested in anymore: the marginal, average treatment effect of our trial.

This mismatch is associated with the so-called *non-collapsibility* of the odds ratio (Groenwold et al., 2011; Daniel et al., 2021; Morris et al., 2022). Much ink has been spilled on this topic, especially in epidemiological research. The best way to understand this phenomenon might be through a concrete example.

Imagine that, as part of our example trial, we also recorded if individuals were currently taking antidepressants, which $n = 182$ (33%) did. Below, we can see the hypothetical results on reliable improvement in the subgroup with and without medication intake. To the right, we also present the counts in the complete sample.

Antidepressants
OR = 5.96
RR = 4.00

	CG	*IG*
Unchanged	82	55
Improved	9	36

No Antidepressants
OR = 3.95
RR = 1.29

	CG	*IG*
Unchanged	55	18
Improved	127	164

Complete Sample
OR = 2.76
RR = 1.47

	CG	*IG*
Unchanged	137	73
Improved	136	200

The table also shows the odds ratio for each specific stratum, and the odds ratio calculated in the entire trial population[3]. This reveals the "oddity" of the odds ratio: in both patient subgroups, the one with antidepressant intake (OR=5.96) and the one without (OR=3.95), the *subgroup-conditional* odds ratio is larger than the *marginal* odds ratio we calculate for the complete sample (OR=2.76).

Even if we attempt to "re-combine" the two conditional odds ratios (antidepressant, no antidepressants) by calculating some (weighted) average of the two, the resulting effect will still hover somewhere between 3.95 and 5.96, which is higher than the unconditional odds ratio we obtain in the entire sample. Thus, the estimates fail to *collapse*. Notably, there are other effect size metrics that do not display this behavior, as is shown by the risk ratios (RR)[4] of both subgroups and the complete sample in the table above.

[3]The odds ratio can be calculated as $\text{OR} = \frac{n_{e_\text{treat}}/(n_\text{treat} - n_{e_\text{treat}})}{n_{e_\text{control}}/(n_\text{control} - n_{e_\text{control}})}$, where n_e is the number of events in the treatment or control group, respectively. For example, for the antidepressant group, this leads to $\frac{36/55}{9/82} \approx 5.96$.

[4]The risk ratio is defined as $\text{RR} = \frac{n_{e_\text{treat}}/n_\text{treat}}{n_{e_\text{control}}/n_\text{control}}$, where n_e is the number of events in the treatment or control group. For the antidepressant subgroup, this leads to $\frac{36/91}{9/91} = 4.00$.

If you find all of this puzzling, you are not alone. There is a lot of confusion surrounding the odds ratio, where its non-collapsibility is often falsely seen as a way of "confounding" or bias (Greenland, 2021; Greenland et al., 1999). In reality, non-collapsibility is a purely mathematical feature of the odds ratio that just happens to be very undesirable in applied research contexts. It suffers from a numerical averaging failure that leads the *average* of the *covariate-specific* treatment effects to differ from the *average treatment effect* itself.

As we mentioned before, the marginal and unconditional odds ratio thus measure two distinct quantities, and problems arise if we start to confuse the two. This is particularly problematic because the covariate-adjusted odds ratios we obtain via logistic regression often tend to be much larger than the marginal effect we are usually interested in within RCT evaluations.

This puts us into a dilemma: on one hand, it is sensible to control for covariates in our analysis model and to do so within a logistic regression, since this usually ensures the convergence of our model. On the other hand, the odds ratio we obtain from this model does not express the treatment effect we actually want to estimate, and it may also be preferable to report collapsible metrics like the risk ratio instead.

An attractive method to "have your cake and eat it too" is to *derive* a marginal risk ratio from the covariate-adjusted logistic regression model that we fitted above. This can be achieved using a method known as *regression standardization* or g-*computation*. We will cover this approach in the next chapter.

5.2.3 | Regression Standardization

The *g*-computation methods we apply in this chapter are often used in epidemiology, where confounding presents a much more acute problem than it typically does in clinical trials. To deal with confounding, researchers frequently tried to adjust for confounding by controlling for specific variables within a regression model, similar to what we did in the linear ANCOVA model, where we included the baseline symptom severity as a covariate.

This was associated with all the problems we discussed before: it changes the interpretation of the estimated treatment effect (from marginal to conditional), where the estimates now depend on the specific set of covariates that one adjusted for. Furthermore, due to the non-collapsibility of measures like the odds ratio, unconditional and conditional effect estimates differ from each other even when there is no confounding at all (Vansteelandt and Keiding, 2011).

One method to circumvent the problems of direct regression adjustment is known as *g* computation (Robins, 1986), parametric *g*-formula (Naimi et al., 2017), marginalization (Morris et al., 2022), or simply regression standardization (Vansteelandt and Keiding, 2011). We use these terms interchangeably in this tutorial. To explain the idea behind this method, we focus on the particular type of regression standardization employed in the `stdReg` package (Sjölander and Dahlqwist, 2021), which we will be used later on.

The idea behind regression standardization is to derive an estimate of the average treatment effect, for example, a risk ratio, from a model that also incorporates the influence of covariates that we measured at baseline. We let $\mathbb{E}[Y|T=t,X]$ denote the mean value of our outcome Y, *given* the specific treatment T, and covariate X. In our example, we use only one covariate, but it is also possible that X is a selection of variables that we wish to control for.

In regression standardization, our goal is to standardize (or *marginalize*) this mean of Y by the distribution of X in our trial sample. In the primer article, we already introduced the concept of *counterfactual* outcomes. Through standardization, we can obtain the counterfactual mean value of Y if everyone in our trial had been assigned to a specific treatment level $T=t$ (i.e., the Internet-based intervention or waitlist). We call this mean value $\mu(t)$:

$$\mu(t)=\mathbb{E}\Big[\mathbb{E}[Y|T=t,X]\Big] \tag{42}$$

We can do this for both groups in our trial, which provides us with two standardized counterfactual mean values: one for the intervention group ($T=t_1$), and another for the control group ($T=t_0$). The difference between these two means is the average treatment effect that we want to

estimate in RCTs: ATE $= \mu(T = t_1) - \mu(T = t_0)$.

To perform this kind of marginalization, we need a model that includes both the effect of treatment T and covariates X on our outcome. As we learned, GLMs (such as logistic regression for binary outcomes) are a suitable way to do this. The great advantage of regression standardization is that we have a lot of flexibility in terms of how the effect of treatment and covariates on Y is modeled. For example, we can also include plausible treatment-covariate interaction (also known as *effect moderators*), which would often make the treatment effect much more difficult to interpret when "conventional" regression adjustment is used.

We use $f(T, X; \boldsymbol{\beta})$ to denote that our analysis model is some type of function that relates the treatment indicator and covariate(s) to our outcome Y through a vector of regression parameters $\boldsymbol{\beta}$. In the previous chapter, we learned that GLMs use a link function $g(\cdot)$ to allow, e.g., binary outcomes to be modeled by a linear predictor. Therefore, we can write down our model formula like this:

$$g(\mathbb{E}[Y|T, X]) = f(T, X; \boldsymbol{\beta}) \tag{43}$$
$$= \beta_0 + \beta_1 T + \beta_2 X + \dots \tag{44}$$

Where the three dots indicate that our linear predictor could also include more terms than an intercept, treatment coefficient, and one covariate weight.

Put simply, the equation above tells us that the first step in regression standardization is to fit the desired GLM for our outcome, as we did previously using the `glm` function. We used logistic regression in our example, but other GLMs link functions (e.g. a Poisson log-link) are also perfectly possible. The crucial step follows next: we use the estimated coefficients of our model $\hat{\boldsymbol{\beta}}$ to predict the outcome Y for each person i in our sample, based on their specific covariate values X_i. This is done while holding the treatment indicator constant: effectively, we predict the outcomes while *pretending* that all our participants were assigned to the same group t.

This is where the counterfactual aspect becomes important. Naturally, not all participants received the specific treatment t (in our example, some received the Internet-based intervention, while others were assigned to the waitlist control). Our goal is rather to predict, based on our model, the outcome of a person with covariates X_i that we *would* expect if this person were assigned to the specific treatment t; no matter if that person actually received this treatment in our trial. If we use a GLM, the inverse link function $g^{-1}(\cdot)$ then has to be used to "translate" the predictions back to the original format. Lastly, we have to average the predictions across participants n to obtain an estimate of the mean $\hat{\mu}(t)$ for our specific treatment t:

$$\hat{\mu}(t) = \frac{1}{n}\sum_{i=1}^{n} g^{-1}\big(f(T = t, X_i, \hat{\boldsymbol{\beta}})\big) \tag{45}$$

We use this method twice: one time to estimate the average outcome in the treatment group $\hat{\mu}(t_1)$, and the second time for the expected mean in the control group $\hat{\mu}(t_0)$. These two quantities can then be used to derive the estimated, marginal treatment effect in the population. For example, if we subtract one mean from the other, we can obtain the risk difference (RD): $\hat{\mu}(t_1) - \hat{\mu}(t_0)$. If we divide the intervention group mean by the control group mean, that is, $\hat{\mu}(t_1)/\hat{\mu}(t_0)$, we get the marginal risk ratio for our trial.

So-called *sandwich estimators* are then employed in the `stdReg` package to calculate standard errors and confidence intervals around the derived effect size. Sandwich estimators are a broadly applicable and commonly used method for robust variance estimation that guards against model misspecification (see e.g. Aronow and Miller, 2019, , chap. 4.2.2).

Do not worry if you do not understand all the computational details that we delineated here. The essential point to understand is that regression standardization is a method that we can use to derive a valid estimate of the *marginal* treatment effect from various types of GLMs that include the influence of covariates. This allows us to use models that have desirable computational properties in the first step, such as logistic regression, and then use standardization to derive the treatment effect estimate that we are actually interested in, such as the marginal risk ratio. In R specifically, the `stdGlm` function within the `stdReg` package can be used to obtain standardized effect estimates

from models that we fitted in the first step using the `glm` function.

Time to put this into practice. Before we illustrate regression standardization for models that we fitted in multiply imputed data, let us first focus on the more simple scenario with only one data set. Below, we fit the same logistic regression model that we also used in the previous chapter, in which we regress reliable improvement on the effect of treatment, while controlling for baseline CES-D scores. However, we only do this in the first imputed data set in `implist` for now. We save the resulting model as `m.glm`:

```
m.glm <- glm(ri ~ group + scale(cesd.0),
             data = implist[[1]], family = binomial("logit"))
```

This is the first step. Next, we use the `stdGlm` to obtain a standardized estimate of the treatment effect on reliable improvement. In the function call, we have to provide the GLM we fitted before, as well as two additional arguments: `X`, which designates the treatment indicator variable (`"group"` in our case), as well as `data`, where we have to provide the data we used to fit the model. We save the results as `m.std`. This leads to the following code:

```
library(stdReg)
m.std <- stdGlm(m.glm, X = "group", data = implist[[1]])
```

The desired effect size can then be returned using the `summary` method. We have to provide the `stdGlm` object we just created, as well as the type of `contrast` (`"ratio"` for the risk ratio, `"difference"` for the risk difference), as well as the `reference` group. In this argument, we should specify the factor level which is used for the control group in our trial data; `0` in our case. First, let us have a look at the estimated risk ratio:

```
summary(m.std, contrast = "ratio", reference = 0)
```

```
## [...]
##   Estimate Std. Error lower 0.95 upper 0.95
## 0     1.00      0.000       1.00       1.00
## 1     1.62      0.127       1.37       1.87
```

The important part of the output is the last line, which tells us the estimated effect is RR=1.62. This effect is significant since the 95% confidence interval (1.37 to 1.87) does not include 1. Naturally, these confidence intervals are still too narrow because the model was only fitted in one imputation set.

We can also generate an estimate of the risk difference by setting `contrast` to `"difference"`:

```
summary(m.std, contrast = "difference", reference = 0)
```

```
## [...]
##   Estimate Std. Error lower 0.95 upper 0.95
## 0    0.000     0.0000      0.000      0.000
## 1    0.235     0.0358      0.165      0.306
```

This tells us that the risk difference between the control and intervention group is RD=0.235, with the 95% confidence interval ranging from 0.165 to 0.306.

To illustrate that `stdGlm` can also be used for a more complex model, let us now fit a GLM in which the treatment effect is allowed to *interact* with three baseline covariates: `cesd.0`, age and sex. We can do this by putting the three variables in brackets in our `glm` formula, and then joining this together with the `group` term using an asterisk (`*`).

```
# Fit model with treatment-covariate interactions
m.glm2 <- glm(ri ~ group*(scale(cesd.0) + scale(age) + sex),
              data = implist[[1]], family = binomial)

# Return marginal risk ratio
m.std2 <- stdGlm(m.glm2, X = "group", data = implist[[1]])
summary(m.std2, contrast = "ratio", reference = 0)
```

```
## [...]
##   Estimate Std. Error lower 0.95 upper 0.95
## 0     1.00      0.000       1.00       1.00
## 1     1.62      0.127       1.37       1.87
```

We see that this also returns an estimate of the ATE expressed as the risk ratio. In this particular case, the results are identical to the ones from before.

Obtaining results from the multiply imputed data is slightly more complex. We have to calculate the marginal risk ratio in all data sets first, and then we have to pool all the estimates as well as construct confidence intervals that incorporate the imputation uncertainty. Let us go through the code step by step.

First, we have to fit the `glm` model in each imputed dataset, and then extract the calculated risk ratio and its standard error using `stdGlm` and `summary`. As before, we do this with the `map` function (or, more precisely, the `map_dfr`). The risk ratios we calculate in each data set should be log-transformed before we pool them. This can be achieved by setting the `transform` argument to `"log"` within `summary`.

```
implist %>%
  map_dfr(function(x){

    # Run GLM and standardize
    std.glm <- glm(ri ~ group + scale(cesd.0), data = x,
                   family = binomial) %>%
                 stdGlm(., X = "group", data = x)

    # Calculate the standardized log-RR
    summary(std.glm, contrast = "ratio", transform = "log",
            reference = 0)$est.table["1", 1:2]

  }) -> std.mi
```

Now, these $m = 50$ log-risk ratio and standard error values have to be pooled into a single estimate using Rubin's rules. This can be achieved by the `pool.scalar` function in `mice`.

```
pool.scalar(
  Q = std.mi$Estimate,
  U = std.mi$`Std. Error`^2)[c("qbar", "t", "df")] -> res.std.rr
```

The `pool.scalar` function returns the pool log-risk ratio and total variance. We can use this information to transform the log-risk ratio back to its original format and construct a confidence interval around it:

```
within(res.std.rr, {

  # Calculate CI based on t-distribution with MI degrees of freedom
```

```
  lower.qbar <- qbar - qt(0.975, df) * sqrt(t)
  upper.qbar <- qbar + qt(0.975, df) * sqrt(t)
  pval <- pt(qbar/sqrt(t), df, lower = FALSE)*2

  # Use antilog to transform logRR values to RR
  rr <- exp(qbar)
  lower.rr <- exp(lower.qbar)
  upper.rr <- exp(upper.qbar)

}) -> res.RR

res.RR[c("rr", "lower.rr", "upper.rr")]
```

```
## $rr
## [1] 1.63489
## $lower.rr
## [1] 1.452034
## $upper.rr
## [1] 1.840772
```

As we can see, this leads to a final value of RR $\approx$ 1.64, with the 95% confidence interval ranging from 1.45 to 1.84. We can thus conclude that the intervention increases the "risk" of reliable improvement by 64%.

The code above is arguably a little complex. However, the most important thing is to get the first block right, in which we define the model to be fitted in each data set, and which marginal effect size should be derived. The rest of the syntax should be transportable "as is" to other RCT analyses.

In a similar way, we can also calculate the pooled risk difference. This time, however, we use a logit-transformation by setting `transform` to `"logit"`:

```
implist %>%
  map_dfr(function(x){

    # Run GLM and standardize
    std.glm <- glm(ri ~ group + scale(cesd.0), data = x,
                   family = binomial) %>%
      stdGlm(., X = "group", data = x)

    # Calculate the standardized log-RR
    summary(std.glm, contrast = "difference", transform = "logit",
            reference = 0)$est.table["1", 1:2]

  }) -> std.mi


pool.scalar(
  Q = std.mi$Estimate,
  U = std.mi$`Std. Error`^2)[c("qbar", "t", "df")] -> res.std.rd

within(res.std.rd, {

  # Calculate CI based on t-distribution with MI degrees of freedom
  lower.qbar <- qbar - qt(0.975, df) * sqrt(t)
  upper.qbar <- qbar + qt(0.975, df) * sqrt(t)
  pval <- pt(qbar/sqrt(t), df, lower = FALSE)*2
```

```
  # Use inverse logit to transform logit RD values to RD
  # We also have to subtract the inverse logit RD for the reference
      group
  rd <- plogis(qbar) - plogis(0)
  lower.rd <- plogis(lower.qbar) - plogis(0)
  upper.rd <- plogis(upper.qbar) - plogis(0)

}) -> res.RD

res.RD[c("rd", "lower.rd", "upper.rd")]
```

```
## $rd
## [1] 0.2311001
## $lower.rd
## [1] 0.1597279
## $upper.rd
## [1] 0.2922169
```

We see that the pooled risk difference is RD $\approx$ 0.23 (95%CI: 0.16 to 0.29). The risk difference is also known as the absolute risk reduction (ARR), although this term arguably makes less sense in our example, in which we want the "risk" (of improvement) to *increase* in our treatment group. The important point is that the risk difference (viz., absolute risk reduction) we calculated here can be used to derive the number needed to treat (NNT), since the NNT is defined as the inverse of the ARR (see Chapter 5.2.1). To obtain valid confidence intervals around the NNT estimate, we also take the inverse of the lower and upper bound of the confidence interval we just calculated. This leads to the following code:

```
as.numeric(res.RD[c("rd", "lower.rd", "upper.rd")])^-1
```

```
## [1] 4.327129 6.260649 3.422115
```

The resulting NNT is 4.32, which means that 5 patients need to receive the treatment to produce one additional case of reliable improvement. This value is very similar to the one we obtained based on the pooled counts in Chapter 5.2.1. One advantage of deriving the NNTs the way we did here is that this directly provides us with an appropriate confidence interval. In our case, the interval ranges from 6.26 (lower bound) to 3.42 (upper bound).

From Zero to Infinity: Confidence Intervals for the NNT

Being defined as the inverse of the ARR, the NNT displays a few numerical properties that can seem odd at first glance. When referring to positive outcomes, *higher* NNT values mean that the intervention performs *worse*; similarly, when the treatment effect is significant, the lower bound of the confidence interval has a higher value than the upper bound.

However, things get even more complicated when the underlying treatment effect is *not* significant (i.e., includes 1 or 0, depending on the metric). In this case, the confidence interval includes infinity, which is the equivalent NNT value of a null effect. Imagine that, in our example from before, we would have instead found a risk difference of 0.03, where the confidence interval ranges from -0.04 to 0.09. This would lead to an NNT of 33, where the upper bound is 11, and the lower bound is -25.

In this hypothetical example, the last value actually does not represent the NNT anymore, but the *number needed to harm* (NNH): we would have to treat 25 patients to prevent one additional positive outcome from happening, due to the negative

effect of the intervention. Notably, these two bounds include the null effect in which there is no difference between the two groups at all, which means that NNT $= \infty$. In this case, Altman (1998) recommended reporting the confidence interval around the NNT in a way that emphasizes that the estimated effect's lower bound is actually a number needed to harm, and that the confidence interval includes infinity. For our example, this would lead to a confidence interval expressed as "NNH 25 to ∞ to NNT 11".

■

PART 6 | Miscellaneous Topics

6.1 | Multiple Testing

In the previous sections, we focused on the fairly straightforward example of a two-arm trial with exactly one primary endpoint. Clinical trials are very costly, and we typically have multiple stakeholders who want to draw insights from them. In practice, this often leads to a whole set of study hypotheses, meaning that more than one test has to be conducted. Typical scenarios are:

- **Multiple primary endpoints**: multiple outcomes have been measured (e.g. symptom severity, quality of life, wellbeing, ...), and we want to confirm a treatment effect for all of them.
- **Multiple assessments of the primary endpoint**: there is only one primary outcome, but it has been measured multiple times, and we want to confirm the treatment effect over the entire study period.
- **Multiple intervention groups**: there is more than one intervention group, and we want to confirm the effectiveness of all the studied interventions compared to the control.
- **Subgroup analyses**: besides the overall intervention effect, we also want to test if the treatment was effective in one or several specific subsets of our sample (e.g. women and men, elderly individuals, ...).

All of these cases can lead to *multiplicity* issues in clinical trials. When conducting a statistical test, we want the nominal significance level to not exceed a predefined threshold, the α level. For better or worse, this level is conventionally set to 0.05, which means that we reject a null hypothesis if $p \leq 0.05$. This ensures that the probability of a type I error, the probability of falsely rejecting the null hypothesis of no effect, does not exceed 5%.

This goal is threatened once we start conducting multiple statistical tests. The more tests we conduct, the likelier it becomes that at least one of them will falsely reject the null hypothesis of no effect. This means that type I error probability starts to increasingly exceed the desired threshold of 5%, in a process that is also known as the *inflation of the family-wise error rate* (FWER; Vickerstaff et al., 2019), or simply α-inflation. This relationship is captured by equation 46 below, where k is the number of tests:

$$\text{P(at least one false-positive result)} = 1 - (1 - \alpha)^k \quad (46)$$

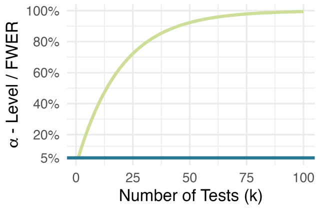

Thus, to avoid an inflation of false-positive findings, and to maintain the nominal significance level despite multiple testing, an α-error correction is necessary. Let us illustrate this using our example trial data.

Imagine we hypothesized that, besides depressive symptom severity, our intervention also has effects on post-test behavioral activation and anxiety levels, as measured by the BADS-SF and HADS-A, respectively. Thus, we now want to confirm that our treatment is effective for three outcomes, and thus three tests have to be conducted. To test the effects, we run the same ANCOVA model already used in Chapter 5.1.3. For each outcome, we use its baseline measurement as the covariate in our model.

```
# ANCOVA for post-test CES-D
with(implist, lm(cesd.1 ~ 1 + group + cesd.0)) %>%
    map_dbl(~anova(.)$`F value`[1]) %>%
    micombine.F(df1=1) -> F.cesd

# ANCOVA for post-test BADS-SF
with(implist, lm(badssf.1 ~ 1 + group + badssf.0)) %>%
    map_dbl(~anova(.)$`F value`[1]) %>%
    micombine.F(df1=1) -> F.badssf

# ANCOVA for post-test HADS-A
with(implist, lm(hadsa.1 ~ 1 + group + hadsa.0)) %>%
    map_dbl(~anova(.)$`F value`[1]) %>%
    micombine.F(df1=1) -> F.hadsa

# Collect all three p-values
(p <- c(F.cesd["p"], F.badssf["p"], F.hadsa["p"]))
```

```
##            p            p            p
## 4.062936e-16 1.521724e-03 5.369427e-08
```

The resulting P values are presented in scientific notation. For example, in the first value, the e-16 part tells us that the number should be multiplied with 10^{-16}. As we can see, all of the P values are well below our threshold of 0.05, which means that we would conclude that the intervention was effective.

Yet, of course, these tests have not been corrected for multiple testing yet, which means that our α level is inflated. Two commonly used methods to adjust for this are the Bonferroni and Holm-Bonferroni (Holm, 1979) correction. The "classical" Bonferroni correction is overly conservative; while correcting for the type I error, it increases the probability of a type II error (i.e., it inflates our risk of keeping the null hypothesis, even when there is a real effect). The Holm-Bonferroni corrects for exactly this problem and may therefore often be preferable in practice unless avoiding false positives is extremely important.

In R, both methods can be implemented using the `p.adjust` function. We only need to provide the vector of P values that should be adjusted for multiple testing, as well as the `method` we want to apply:

```
p.adjust(p, method = "bonferroni")
```

```
##            p            p            p
## 1.218881e-15 4.565173e-03 1.610828e-07
```

```
p.adjust(p, method = "holm")
```

```
##             p             p             p
## 1.218881e-15 1.521724e-03 1.073885e-07
```

We see that, for both methods, the corrected P values remain well below 0.05. Thus, we can conclude that our intervention had effects not only on depressive symptom severity but also on behavioral activation and symptoms of anxiety.

The merits of multiplicity adjustments remain a controversial topic in clinical trial evaluations and elsewhere (Rothman, 1990; Althouse, 2016; Gelman et al., 2012; Gelman and Loken, 2013). The idea of α correction is closely linked to the frequentist branch of statistics; Bayesian statisticians think quite differently about this topic (Sjölander and Vansteelandt, 2019; Gelman et al., 2012).

Logical Issues of Multiplicity Adjustments

Althouse (2016) summarizes a few of the logical inconsistencies of multiplicity adjustments that have been described in the literature. A core criticism is that the number of tests we should adjust for is very much open to interpretation. Should it be for all tests conducted within a specific paper? This would mean that we can avoid adjusting our P value if we simply publish a separate paper for each hypothesis that we have. Such an approach would also "punish" larger, resource-intensive trials, since they typically include more outcomes than exploratory trials. What if other researchers also work on our trial data–should we adjust for *their* tests as well?

At the most extreme, we would have to adjust for all tests we expect to conduct during our scientific career. This would evidently be absurd, apart from being practically impossible.

Despite the logical inconsistencies we described in the box above, multiple testing can indeed jeopardize our inferences in certain contexts, so we have to draw the line somewhere. Below, we summarize a few practical recommendations on how to deal with multiple testing in practice (Li et al., 2017):

- **Multiple outcomes**: A correction is only necessary if all outcome tests are considered *confirmatory*. If there is only one primary and several secondary endpoints, no correction is necessary. However, the results of secondary endpoints are only exploratory, not confirmatory evidence! They cannot "prove" that the intervention is effective for these outcomes, and further studies are needed to confirm that it is.
- **Multiple assessments**: If only one assessment point is pre-specified as confirmatory-primary, no correction is necessary; likewise for tests where modeling is performed across multiple measurement points (e.g. using mixed models). In the next section, we will describe in greater detail how to deal with longitudinal data.
- **Multiple intervention groups**: A correction is necessary when the intervention groups are "related" (e.g., the same medication at different doses; or the same self-guided intervention with and without human support). Adjustments may be of less importance when the interventions are clearly distinct (e.g. antidepressants versus cognitive-behavioral treatment, where both are provided as monotherapies).

6.2 | Longitudinal Data

Nowadays, in most mental health research RCTs, patients are followed up multiple times to assess how effects develop over time. For psycho-behavioral interventions, a frequently used method is to include a post-test assessment as well as (one or multiple) long-term follow-ups. Post-test assessments are usually conducted around the time the intervention is expected to be completed, while long-term follow-ups allow to check if the effects stabilize over time. In pharmacological

research, it is also commonplace to have multiple visits to determine how patients respond over time.

As we discussed, clinical trials usually have only one primary endpoint, which is strictly defined as one specific comparison, measured by a specific instrument, at or within a specific time. Thus, if we have multiple measurements within the trial period of interest, these must be incorporated into a single analysis model to avoid multiple testing. There are several recommended approaches to analyze longitudinal data in RCTs, first and foremost linear mixed-effects models and generalized estimating equations (GEE; Twisk, 2021, chap. 3.4). Here, we will showcase a longitudinal analysis using a special type of mixed-effects model known as *mixed-model repeated measures* (MMRM; Mallinckrodt et al., 2008).

Like conventional mixed models, MMRMs allow us to explicitly model that multiple assessments are available for each patient, and that these values are therefore correlated. In a "vanilla" mixed-effects model, this nested data structure (assessment points-in-patients) is often modeled by including a random intercept for each patient. These random intercepts are assumed to follow a normal distribution with mean zero and variance τ^2. Let us briefly flesh this out in a model formula:

$$Y_{it} = \beta_0 + u_i + \beta_1 T_{it} + \beta_2 x_{it} + \epsilon_{it} \tag{47}$$

$$\epsilon_{it} \sim \mathcal{N}(0, \sigma^2) \qquad u_i \sim \mathcal{N}(0, \tau^2) \tag{48}$$

This equation tells us that the outcome Y_{it} of person i at assessment point t is predicted by two types of regression terms: the *fixed* effects captured by the regression coefficients β, as well as the random participant effect captured by the u_i term. The latter is a participant-specific value that shifts the intercept β_0 up or down for that specific person, and the formula above is therefore known as a random intercept model.

We can also see that the fixed terms are equivalent to the ones we used in the linear ANCOVA model in Chapter 5.1.1: we have one coefficient for the effect of our treatment T, while β_2 captures the effect of the baseline covariate X. Naturally, this model could also include more than one baseline covariate.

MMRMs try to capture the dependencies in longitudinal data in a slightly different way. They do not include special random effects in the model, but instead, specify that the residual errors ϵ_{it} are correlated in a particular way. Equation 47 then simplifies to:

$$Y_{it} = \beta_0 + \beta_1 T_{it} + \beta_2 x_{it} + \epsilon_{it} \tag{49}$$

$$\epsilon \sim \mathcal{N}(0, \mathbf{\Omega}) \qquad \epsilon_i \sim \mathcal{N}(0, \mathbf{\Sigma}_i) \tag{50}$$

The special part of this equation is that we now allow for patient-specific variance-covariance matrices $\mathbf{\Sigma_i}$, which are assembled in a block-diagonal matrix $\mathbf{\Omega}$. Unstructured variance-covariance matrices of this form can be used within MMRMs to model the dependencies in our data, which removes the need to include random effects in the formula above.

This was a brief and somewhat technical introduction. The main takeaway is this: in RCT evaluations, we are typically only interested in fixed effects (e.g. the overall effect of our treatment). MMRMs allow us to estimate exactly that in longitudinal data, without the need to specifically define random effects, and while accounting for the dependencies in our data.

To illustrate MMRMs in practice, we will use the `mmrm` function, which is included in the eponymous package (Sabanés Bové et al., 2023). We will use this function to perform a longitudinal analysis of treatment effects on depressive symptoms, which were measured at a 7-week post-test (`cesd.1`) and 12-week follow-up (`cesd.2`).

To do this, we first have to transform our imputed data sets included in `implist` into the long format. We have already done this before in Chapter 4.2.3, where we prepared our data for the reference-based imputation, and similar code can also be applied here. We save the result as `implist.long`:

```
implist %>%
  map(function(x){
    x %>%
      mutate(id = 1:nrow(x)) %>%
      pivot_longer(ends_with(c(".1", ".2")),
                   values_to = "value",
                   names_to = c("outcome", "time"),
                   names_pattern = "(.*).(.)") %>%
        mutate(time = recode(time, `1` = 7, `2` = 12))
  }) -> implist.long
```

Let us have a look at the resulting data format:

```
# Extract a slice of the first imputed data set
implist.long[[1]][1:10, c("id", "outcome", "time", "value",
                          "cesd.0", "badssf.0", "hadsa.0")]
```

```
##       id outcome  time value cesd.0 badssf.0 hadsa.0
##   1    1 badssf      7    20     24       24       7
##   2    1 hadsa       7     5     24       24       7
##   3    1 cesd        7    33     24       24       7
##   4    1 badssf     12    18     24       24       7
##   5    1 hadsa      12    10     24       24       7
##   6    1 cesd       12    30     24       24       7
##   7    2 badssf      7    24     38       33      11
##   8    2 hadsa       7    11     38       33      11
##   9    2 cesd        7    38     38       33      11
##  10    2 badssf     12    28     38       33      11
```

We see that there are now several rows for each participant as indicated by the id variable. For each patient, we have follow-up data from two measurement points (time=7 and time=12), with three outcomes (badssf, hadsa, and cesd) each. Importantly, the baseline covariates are stored as separate columns and have constant values within patients.

For this analysis, we are only interested in the cesd outcome. Thus, we have to extract this outcome from all imputed data sets. We also convert the id, group, and time variables to factors, since this is a requirement of the mmrm function.

```
implist.long %>%
  map(~ filter(., outcome == "cesd") %>%
        mutate(id = as.factor(id),
               group = as.factor(group),
               time = recode(time, `7` = "7 wks", `12` = "12 wks") %>%
                 as.factor())
      ) -> implist.long
```

Now, let us fit our first MMRM model. For now, we focus only on the first imputed data set. As fixed terms in our formula, we include group and the baseline CES-D values, as we did in previous models. Then, we use the us function[5] in the formula to specify the variance-covariance structure. Here, we use a model that also allows the variance-covariance matrices to vary between trial arms, which leads to us(time|group/id).

Additionally, we set the method argument to "Kenward-Roger", which means that the method by Kenward and Roger (1997) is used to construct confidence intervals and perform statistical tests.

[5]"us" stands for *unstructured* (variance-covariance matrix).

This approach is especially recommended for smaller data sets. We save the results as `m.mmrm`, and then plug this object into the `summary` function:

```
library(mmrm)
m.mmrm <- mmrm(value ~ group + scale(cesd.0) + us(time|group/id),
               method = "Kenward-Roger", data = implist.long[[1]])
summary(m.mmrm)
```

```
## [...]
## Coefficients:
##                Estimate Std. Error       df t value Pr(>|t|)
## (Intercept)     22.9220     0.3965 271.8000  57.814   <2e-16 ***
## group1          -6.0220     0.5435 540.8000  11.080   <2e-16 ***
## scale(cesd.0)  -0.2975     0.2727 542.7000   1.091    0.276
## ---
## Signif. codes:  0 ‘***’ 0.001 ‘**’ 0.01 ‘*’ 0.05 ‘.’ 0.1 ‘ ’ 1
##
## Covariance estimate:
## Group: 0
##          12 wks   7 wks
## 12 wks 105.2159 -1.7908
## 7 wks   -1.7908 74.8074
## Group: 1
##         12 wks   7 wks
## 12 wks 85.8970 -2.9045
## 7 wks  -2.9045 71.8065
```

This provides us with an estimate of the intervention effect across the 12-week study period, $\hat{\beta}_{\text{group}}$ = -6.02. Below, we are also presented with the (co)variance estimates for the two groups across both measurement points.

To fit this model in all imputed data sets, we can use the `map` function. Then, as before, we pool the parameter estimates using the `testEstimates` function in the `mitml` package:

```
implist.long %>%
  map(~ mmrm(value ~ group + scale(cesd.0) + us(time|group/id),
             method = "Kenward-Roger", data = .)) %>%
  testEstimates()
```

```
## [...]
##                Estimate Std.Error t.value      df P(>|t|)   RIV   FMI
## (Intercept)      23.022     0.473  48.698 483.087   0.000 0.467 0.321
## group1           -6.082     0.616  -9.865 815.571   0.000 0.325 0.247
## scale(cesd.0)    -0.224     0.307  -0.730 838.243   0.466 0.319 0.244
## [...]
```

This leaves us with a very similar estimate of the treatment effect, $\hat{\beta}_{\text{group}}$ =-6.082, which is significant (p <0.001). To derive a standardized effect size (i.e., Cohen's d), we have to divide this estimate by the pooled standard deviation (see Chapter 5.1.4). A simple way to obtain this value would be to take the average of s_{pooled} in both measurement points. We omit this step here.

Next, we can also fit a slightly more sophisticated model in which the group term interacts with time. We can do this by joining `group` and `time` together with an asterisk (`*`):

```
implist.long %>%
  map(~ mmrm(value ~ group*time + scale(cesd.0) + us(time|group/id),
```

```
             method = "Kenward-Roger", data = .)) %>%
  testEstimates()
```

```
## [...]
##                    Estimate Std.Error t.value      df P(>|t|)   RIV   FMI
## (Intercept)          22.555     0.757  29.799 531.492   0.000 0.436 0.306
## group1               -4.769     0.977  -4.881 833.057   0.000 0.320 0.244
## time7 wks             0.785     0.980   0.801 659.669   0.424 0.375 0.275
## scale(cesd.0)        -0.224     0.307  -0.730 838.244   0.466 0.319 0.244
## group1:time7 wks     -2.355     1.341  -1.757 794.252   0.079 0.330 0.250
## [...]
```

The output is a little more difficult to decipher now. Since `time` is a factor variable, it is automatically dummy-coded by the function; and since `"12 wks"` comes alphabetically before `"7 wks"`, it is used as the reference category in this model. This means that `group1` now represents the treatment effect after 12 weeks (i.e., when `time=0`). We see that this value is slightly lower than the combined estimate ($\hat{\beta} =$ -4.769) but still significant. This lets us conclude that the effects of treatment taper off slightly with time, but are still largely maintained after twelve weeks.

To obtain the effect estimate after 7 weeks, we need to look at the `"group1:time7 wks"` term. This tells us by how much we must adjust the "original" estimate captured by `group1` to get the effect after 7 weeks. We can do this by adding the two point estimates together, which gives -4.769 - 2.355 = -7.124. This is the average treatment effect at post-test based on our model.

Unfortunately, since the 7-week effect is only defined in reference to the one after 12 weeks, its P value can not be interpreted as a test of the treatment effect itself. We see that the effect of `"group1:time7 wks"` is not significant ($p =$0.079), but this only means that the treatment effects after 7 and 12 weeks do not differ significantly from each other.

To test the average effect after 7 weeks, we can use a little trick. In all of our imputed data sets, we recode the factor levels so that `"7 wks"` now comes before `"12 wks"`, and is therefore used as the new reference category. This can be achieved using the `levels` argument in the `factor` function, where we have to provide the factor levels in the exact order in which we want them to appear. We save this model as `m.mmrm.7wk`. This leads to the following code:

```
implist.long %>%
  map(~ mutate(., time =
                 factor(time, levels = c("7 wks", "12 wks")))) %>%
  map(~ mmrm(value ~ group*time + scale(cesd.0) + us(time|group/id),
             method = "Kenward-Roger", data = .)) %>%
  testEstimates() -> m.mmrm.7wk

m.mmrm.7wk
```

```
## [...]
##                     Estimate Std.Error t.value      df P(>|t|)   RIV   FMI
## (Intercept)           23.340     0.613  38.050 586.915   0.000 0.406 0.291
## group1                -7.124     0.851  -8.376 770.393   0.000 0.337 0.254
## time12 wks            -0.785     0.980  -0.801 659.670   0.424 0.375 0.275
## scale(cesd.0)         -0.224     0.307  -0.730 838.242   0.466 0.319 0.244
## group1:time12 wks      2.355     1.341   1.757 794.253   0.079 0.330 0.250
## [...]
```

As we can see, the 7-week assessment now serves as the reference group, to which the 12-week assessment is compared. The `group1` term now directly shows us the 7-week effect (-7.124), where the point estimate is identical to the one we derived in the previous model. However, now we are also presented with a statistical test for this term, which tells us that the effect is also

significant (p <0.001). Like in Chapter 5.1.4, we can also use the `confint` function here to obtain valid confidence intervals around this estimate:

```
confint(m.mmrm.7wk)
```

```
##                         2.5 %     97.5 %
## [...]
## group1              -8.7934372 -5.4542877
## [...]
```

To calculate confidence intervals for the 12-week effect, we would have to plug the *previous* model into the `confint` function instead.

Mixed Models and Missing Data

In our longitudinal analysis above, MMRMs were fitted in the multiply imputed data, and we then calculated the pooled parameter estimates. Some researchers would probably disagree with this approach and argue that mixed models alone are already sufficient to deal with missing values under the MAR assumption (see Chapter 4.1.2), meaning that multiple imputation is not needed here.

It has indeed been found that linear mixed models can provide valid estimates under MAR even without multiple imputations (Twisk et al., 2013), and this approach is now commonly seen in practice. Unfortunately, this is often used as a blanket statement to argue why multiple imputation was not considered. In practice, things are often a little more difficult.

In Chapter 4.2.1, we learned that multiple imputation models should include *auxiliary variables* to increase the plausibility of the MAR assumption. If we do not use multiple imputation, this task is left to the mixed model itself, and how it is specified. In the MMRM model we fitted above, only baseline symptoms were used as a covariate. We presume that most longitudinal models in practice will not include many more variables than this either. Yet, to satisfy the MAR assumption, we need covariates that predict dropout, and it is questionable if this can be explained by the 1 or 2 covariates we typically include in a mixed model.

This problem can be solved by building a multiple imputation model with plausible predictors of missingness first and then fitting a mixed model in the multiply imputed data that actually addresses our research question. Another advantage is that we can also fit the same type of model to data sets that have been imputed under varying assumptions. For example, we could have also conducted an additional analysis within the "jump-to-reference"-imputed data (see Chapter 4.2.3) to assess longitudinal effects under an MNAR assumption.

We refer the reader to Magnusson (2019) for a more detailed explanation of the argument we presented here.

6.3 | Reporting & Reproducibility

In the final section of our tutorial, we want to provide a little guidance on how to report the results of an RCT evaluation in a scientific paper. As we mentioned before (see Chapter 3), the CONSORT guidelines and checklist should be seen as minimal standards that every RCT report should follow. Besides the CONSORT flow chart we created in Chapter 3, our article should also include a table with descriptives for all measured variables. In the following, we provide a worked example of how to create such a table based on our multiply imputed data.

For the code below to work, we have to let R "learn" a function that we have written specifically for this purpose. To do this, you have to copy the code available here in its entirety, paste it into your R Studio console, and then hit Enter. Please note that the function requires the `skimr`, `dplyr`, and `purrr` packages to be installed on our computer.

In the following, we want to create a descriptive table for the intervention group, control group, and for our entire sample. As a first step, we have to define the number of imputation sets `m` that we created, as well as a vector with the names of all categorical variables (e.g. sex, employment, ...) that we save under the name `catvars`.

```
m <- length(implist)
catvars <- c("sex", "prevpsychoth", "prevtraining",
             "child", "employ", "rel", "degree", "group")
```

Next, we make sure that all these categorical variables are converted to factor vectors:

```
implist %>%
  map(~mutate_at(., catvars, as.factor)) -> implist
```

Then, we can use the `skimReport` function that we just saved in our environment to create the descriptive tables for the complete sample (`num.desc.full`), intervention group (`num.desc.ig`), and control group (`num.desc.cg`). The code below is somewhat involved but should be easy to reuse for other multiply imputed data, as long as it is saved as an `mitml.list` with the name `implist`.

```
implist %>%
  map(~skimReport(.)) %>%
  {.[[1]]$numerics[,1] ->> var.names;.} %>%
  map(~.$numerics[,2:4]) %>%
  Reduce(`+`, .)/m -> num.desc.full

implist %>%
  map(~filter(., group == 1)) %>%
  map(~skimReport(.)) %>%
  map(~.$numerics[,2:4]) %>%
  Reduce(`+`, .)/m -> num.desc.ig

implist %>%
  map(~filter(., group == 0)) %>%
  map(~skimReport(.)) %>%
  map(~.$numerics[,2:4]) %>%
  Reduce(`+`, .)/m -> num.desc.cg
```

Lastly, we bind the three objects together to create the complete table.

```
tab.desc <- cbind(var.names, num.desc.full, num.desc.ig, num.desc.cg)
tab.desc
```

```
##    var.names   mean     sd   n   mean     sd   n   mean     sd   n
## [...]
## 5     cesd.2 20.170 10.217 546 17.786  9.397 273 22.555 10.454 273
## 6     cesd.1 19.777  9.292 546 16.216  8.642 273 23.339  8.528 273
## 7        age 28.245 11.332 546 28.179 11.647 273 28.311 11.029 273
## 8     cesd.0 26.609  7.129 546 26.604  6.990 273 26.615  7.278 273
## [...]
```

As we can see, this gives as the mean, standard deviation and sample size for all continuous outcomes at all measurement points. The left part of the table represents the full sample, the center the intervention group, while the right part contains descriptives for the control group.

If the `openxlsx` package is loaded, we can also save this table as an MS *Excel* file using the `write.xlsx` function:

```
library(openxlsx)
write.xlsx(tab.desc, file="imputed_descriptives.xlsx")
```

Another frequently asked question is how to report the results of the main effectiveness analysis. In most trials, multiple ANCOVAs are run, either because there were several primary endpoints, or because exploratory outcomes were also analyzed. In this case, it makes sense to assemble the results in a table.

Say that we want to report the results of the analysis in Chapter 6.1, in which we treated depressive symptom severity (measured by the CES-D), behavioral activation (measured by the BADS-SF) and HADS-A anxiety as *co-primary* endpoints. One way to do this is to create a table like the one below:

	Regression Coefficient				ANCOVA			Effect Size	
Outcome	$\hat{\beta}_{\text{treat}}$	S.E.	FMI	RIV	F	d.f.	p^a	d	95%CI
Depress. Symptoms	-7.124	0.851	0.254	0.337	69.384	1, 722.34	<0.001	-0.83	[-1.02; -0.64]
Behav. Activation	2.178	0.711	0.305	0.434	10.146	1, 598.23	0.002	0.31	[0.11; 0.52]
Anxiety Symptoms	-1.961	0.349	0.331	0.489	30.666	1, 426.11	<0.001	-0.59	[-0.79; -0.38]

[a]Adjusted for multiple testing (Holm-Bonferroni adjustment).

This provides us with the core outcomes of our linear regression model and ANCOVA: the estimated treatment coefficient $\hat{\beta}_{\text{treat}}$ along with its standard error, as well as the fraction of missing information (FMI) and relative increase in variance (RIV) value. The next section displays the results of the F-test, and the last part gives the calculated effect size and confidence interval.

Code & Data Sharing

Although "open science" practices have become increasingly prevalent in many fields, the sharing of code and data in publications of mental health RCTs remains quite rare. The International Committee of Medical Journal Editors (ICMJE) released a statement that effective 2018, mandates a data-sharing *statement* for all clinical trials (Taichman et al., 2017). However, this does not mandate data sharing itself, and a large gap between the declared and actual availability of data has been found in the literature (Naudet et al., 2018; Danchev et al., 2021).

This is unfortunate; open research materials are usually an excellent way to allow others to examine how we arrived at our results, and facilitate their reproducibility. One way to make research materials openly available is to create an online repository on the OSF (Open Science Framework; osf.io). This allows to upload the R code we used for the analysis, as well as the de-identified trial data itself, and to state under which conditions other researchers can use it. Data sharing is especially helpful for meta-analytic researchers, who often require individual participant data (IPD) of several trials to examine treatment effects across different contexts.

If you decide to make your trial data publicly available, you may also consult the FAIR principles, which state that digital data sets should be **F**indable, **A**ccessible, **I**nteroperable, and **R**eusable.

■

PART 7 | Further Reading

This tutorial should at best be seen as a brief introduction to RCT evaluations, and how they can be implemented using R. Please note that we only covered parallel two-arm randomized controlled trials here, which can be regarded as the most basic RCT design. In practice, many other "flavors" of RCTs are used to do clinical research, including cluster-randomized, cross-over, or stepped-wedge trials, all of which require their own analytic approach. An accessible introduction to these advanced trial designs and their analysis is provided in Twisk (2021), which also provides hands-on examples using STATA.

To learn more about multiple imputation and how to implement it using R, we refer the reader to the book *Flexible Imputation of Missing Data* written by Stef van Buuren, the maintainer of the `mice` package (van Buuren, 2018). This book is openly available online. Another accessible overview of applied missing data handling can be found in Enders (2022). A recommended resource to learn more about R and the tidyverse is *R for Data Science* by Wickham and Grolemund (2016), which is also openly accessible (see here).

■

Test Your Knowledge: Solutions

1. Log-transform the variable age in data and save the result as age.log.

```
age.log <- log(data$age)
```

2. Square all values in cesd.1 and save the result as cesd.1.squared *within* the data set data.

```
data$cesd.1.squared <- data$cesd.1^2
```

3. Calculate the mean and standard deviation (SD) of the variable cesd.2. If necessary, use the Internet to find out which function in R calculates the standard deviation of a numeric vector.

```
mean(data$cesd.2, na.rm=TRUE)
sd(data$cesd.2, na.rm=TRUE)
```

4. Save the calculated mean and standard deviation of cesd.2 in a list element.

```
list(mean = mean(data$cesd.2, na.rm=TRUE),
     sd = sd(data$cesd.2, na.rm=TRUE))
```

5. Does the variable atsphs.0 in data have the desired class numeric? Try to confirm this using R code.

```
class(data$atsphs.0)
```

6. Create two new variables in data: (1) age.65plus, a logical variable which indicates if a person's age is 65 or above; and (2) cesd.diff, a variable that contains the difference between cesd.0 and cesd.1 for each patient.

```
data$age.65plus <- data$age >= 65
data$cesd.diff <- data$cesd.0 - data$cesd.1
```

7. Using the pipe operator, filter out the records of all patients who are male (sex=0) and part of the intervention group (group=1); then, in this subset, select the variable age and calculate its mean.

```
data %>%
  filter(sex==0, group==1) %>%
  pull(age) %>%
  mean()
```

8. In the fifth and sixth row of data, change the value of degree to NA (missing).

```
data[5:6,"degree"] <- c(NA, NA)
```

R Package Information

All code included was tested under R version 4.2.0. The following package versions were used:

```
dplyr_1.0.10        openxlsx_4.2.5      scales_1.2.0
mice_3.14.0         plot.matrix_1.6.2   skimr_2.1.4
miceadds_3.12-26    psych_2.2.3         stdReg_3.4.1
mitml_0.4-3         purrr_0.3.4         tidyr_1.2.0
mmrm_0.2.2.9013     RefBasedMI_0.1.0    tidyverse_1.3.1
```

Data & Downloads

DOI 10.5281/zenodo.7757478

The data we use in this tutorial has been uploaded to a Github repository, which can be found at github.com/MathiasHarrer/rct-tutorial. The repository has also been permanently archived on Zenodo. We also uploaded an R script that includes all the code we used in this tutorial. Quick download links are provided below.

- **`rct-tutorial.zip`** (Download). A zip-folder containing the example trial data set (see `data.xlsx`), as well as the complete tutorial code (see `code.R`).
- **`data.xlsx`** (Download). The original, unimputed example data set, which includes simulated patient data of a randomized controlled trial comparing an Internet-based depression intervention to a waitlist control (N=546).
- **`imp.rda`** (Download). The imputed data set object generated by the `mice` function (see Chapter 4.2.2). After being imported into R, the object has the name `imp`.
- **`imp.j2r.rda`** (Download). The imputed data set object generated by the `RefBasedMI` function ("jump-to-reference" imputation; see Chapter 4.2.3). After being imported into R, the object has the name `imp.j2r`.
- **`implist.rda`** (Download). The `imp` object transformed to a list of imputed data sets (`mitml.list`). This multiply imputed data format can be used to pool analyses using the `testEstimates` function (see Chapter 5.1.3).
- **`implist.j2r.rda`** (Download). The `imp.j2r` object transformed to a list of imputed data sets (`mitml.list`). This multiply imputed data format can be used to pool analyses using the `testEstimates` function (see Chapter 5.1.3).
- **`code.R`** (Open). All code used in the tutorial, collected in one script.
- **`skimReport.R`** (Open). Code of the `skimReport` function, which is used in Chapter 6.3 to generate descriptive tables.

To open `.rda` files, save them in your analysis folder. Then, open the folder through the *Files* pane in R Studio and click on the `.rda` file. This automatically imports the object into your R environment. To see how the `data.xlsx` *Excel* file can be imported, see Chapter 2.3.

Errata & Corrections

Statistical software is constantly evolving, and it is possible that some of the code provided in this tutorial may stop working over time. To ensure you have the most up-to-date information, we also created a "living" digital version of the tutorial at rct-tutorial.mharrer.dev. This online resource will be regularly updated to incorporate software changes or correct potential errors.

References

Althouse, A. D. (2016). Adjust for multiple comparisons? It's not that simple. *The Annals of Thoracic Surgery*, 101(5):1644–1645. DOI: 10.1016/j.athoracsur.2015.11.024. [p79]

Altman, D. G. (1998). Confidence intervals for the number needed to treat. *BMJ*, 317(7168):1309–1312. DOI: 10.1136/bmj.317.7168.1309. [p77]

Aronow, P. M. and Miller, B. T. (2019). *Foundations of agnostic statistics*. Cambridge University Press, 1st edition. DOI: 10.1017/9781316831762. [p72]

Assmann, S. F., Pocock, S. J., Enos, L. E., and Kasten, L. E. (2000). Subgroup analysis and other (mis)uses of baseline data in clinical trials. *The Lancet*, 355(9209):1064–1069. DOI: 10.1016/S0140-6736(00)02039-0. [p51]

Barnard, J. and Rubin, D. B. (1999). Miscellanea. small-sample degrees of freedom with multiple imputation. *Biometrika*, 86(4):948–955. DOI: 10.1093/biomet/86.4.948. [p44, 46]

Bartlett, J. (2021). *mlmi: Maximum Likelihood Multiple Imputation*. R package version 1.1.1. [p41]

Bartlett, J., Keogh, R., and Bonneville, E. F. (2022). *smcfcs: Multiple Imputation of Covariates by Substantive Model Compatible Fully Conditional Specification*. R package version 1.7.1. [p44]

Bartlett, J. W. (2023). Reference-based multiple imputation—what is the right variance and how to estimate it. *Statistics in Biopharmaceutical Research*, 15(1):178–186. DOI: 10.1080/19466315.2021.1983455. [p40]

Bartlett, J. W. and Hughes, R. A. (2020). Bootstrap inference for multiple imputation under uncongeniality and misspecification. *Statistical Methods in Medical Research*, 29(12):3533–3546. DOI: 10.1177/0962280220932189. [p40]

Bartlett, J. W., Seaman, S. R., White, I. R., Carpenter, J. R., and Alzheimer's Disease Neuroimaging Initiative (2015). Multiple imputation of covariates by fully conditional specification: accommodating the substantive model. *Statistical Methods in Medical Research*, 24(4):462–487. DOI: 10.1016/j.cpr.2020.101827. [p43]

Bauer-Staeb, C., Kounali, D.-Z., Welton, N. J., Griffith, E., Wiles, N. J., Lewis, G., Faraway, J. J., and Button, K. S. (2021). Effective dose 50 method as the minimal clinically important difference: Evidence from depression trials. *Journal of Clinical Epidemiology*, 137:200–208. DOI: 10.1016/j.jclinepi.2021.04.002. [p67]

Bell, M. L., Fiero, M., Horton, N. J., and Hsu, C.-H. (2014). Handling missing data in RCTs; a review of the top medical journals. *BMC Medical Research Methodology*, 14(1):1–8. DOI: 10.1186/1471-2288-14-118. [p25]

Bell, M. L., Kenward, M. G., Fairclough, D. L., and Horton, N. J. (2013). Differential dropout and bias in randomised controlled trials: when it matters and when it may not. *BMJ*, 346. DOI: 10.1136/bmj.e8668. [p25]

Busby, M. (2020). Cambridge college to remove window commemorating eugenicist. *The Guardian*. [p1]

Carpenter, J. R., Roger, J. H., and Kenward, M. G. (2013). Analysis of longitudinal trials with protocol deviation: a framework for relevant, accessible assumptions, and inference via multiple imputation. *Journal of Biopharmaceutical Statistics*, 23(6):1352–1371. DOI: 10.1080/10543406.2013.834911. [p40]

Carpenter, J. R. and Smuk, M. (2021). Missing data: A statistical framework for practice. *Biometrical Journal*, 63(5):915–947. DOI: 10.1002/bimj.202000196. [p25, 31, 54]

Christensen, P. M. and Kristiansen, I. S. (2006). Number-needed-to-treat (NNT)–needs treatment with care. *Basic & Clinical Pharmacology & Toxicology*, 99(1):12–16. DOI: 10.1111/j.1742-7843.2006.pto$_4$12.x. [p63]

Cohen, J. (1988). *Statistical power analysis for the behavioral sciences*. L. Erlbaum Associates, 2nd edition. DOI: 10.4324/9780203771587. [p61]

Cohen, J. (1994). The earth is round (p<. 05). *American Psychologist*, 49(12):997–1003. DOI: 10.1037/0003-066X.49.12.997. [p66]

Cro, S., Morris, T. P., Kenward, M. G., and Carpenter, J. R. (2020). Sensitivity analysis for clinical trials with missing continuous outcome data using controlled multiple imputation: a practical guide. *Statistics in Medicine*, 39(21):2815–2842. DOI: 10.1002/sim.8569. [p39]

Csárdi, G., Hester, J., Wickham, H., Chang, W., Morgan, M., and Tenenbaum, D. (2021). *remotes: R Package Installation from Remote Repositories, Including GitHub*. R package version 2.4.2. [p41]

Cuijpers, P., Turner, E. H., Koole, S. L., van Dijke, A., and Smit, F. (2014). What is the threshold for a clinically relevant effect? the case of major depressive disorders. *Depression and Anxiety*, 31(5):374–378. DOI: 10.1002/da.22249. [p61]

Danchev, V., Min, Y., Borghi, J., Baiocchi, M., and Ioannidis, J. P. (2021). Evaluation of data sharing after implementation of the international committee of medical journal editors data sharing statement requirement. *JAMA Network Open*, 4(1). DOI: 10.1001/jamanetworkopen.2020.33972. [p86]

Daniel, R., Zhang, J., and Farewell, D. (2021). Making apples from oranges: Comparing noncollapsible effect estimators and their standard errors after adjustment for different covariate sets. *Biometrical Journal*, 63(3):528–557. DOI: 10.1002/bimj.201900297. [p70]

Dayim, A. (2023). *consort: Create Consort Diagram*. R package version 1.1.0. [p20]

de Silva, A. P., Moreno-Betancur, M., de Livera, A. M., Lee, K. J., and Simpson, J. A. (2017). A comparison of multiple imputation methods for handling missing values in longitudinal data in the presence of a time-varying covariate with a non-linear association with time: a simulation study. *BMC Medical Research Methodology*, 17:1–11. DOI: 10.1186/s12874-017-0372-y. [p27]

Devilly, G. J. and Borkovec, T. D. (2000). Psychometric properties of the credibility/expectancy questionnaire. *Journal of Behavior Therapy and Experimental Psychiatry*, 31(2):73–86. DOI: 10.1016/s0005-7916(00)00012-4. [p17]

Devji, T., Carrasco-Labra, A., and Guyatt, G. (2021). Mind the methods of determining minimal important differences: three critical issues to consider. *BMJ Mental Health*, 24(2):77–81. DOI: 10.1136/ebmental-2020-300164. [p66]

Dunn, P. K. and Smyth, G. K. (2018). *Generalized linear models with examples in R*, volume 53. Springer, 1st edition. DOI: 10.1007/978-1-4419-0118-7. [p50, 67]

Ebert, D. D., Buntrock, C., Lehr, D., Smit, F., Riper, H., Baumeister, H., Cuijpers, P., and Berking, M. (2018). Effectiveness of web-and mobile-based treatment of subthreshold depression with adherence-focused guidance: a single-blind randomized controlled trial. *Behavior Therapy*, 49(1):71–83. DOI: 10.1016/j.beth.2017.05.004. [p16]

Egbewale, B. E., Lewis, M., and Sim, J. (2014). Bias, precision and statistical power of analysis of covariance in the analysis of randomized trials with baseline imbalance: a simulation study. *BMC Medical Research Methodology*, 14(1):1–12. DOI: 10.1186/1471-2288-14-49. [p52]

EMA (2015). Guideline on adjustment for baseline covariates in clinical trials. [p52]

Enders, C. K. (2010). *Applied missing data analysis*. Guilford Publications, New York City, 1st edition. ISBN: 978-1606236390. [p26]

Enders, C. K. (2022). *Applied missing data analysis.* Guilford Publications, New York City, 2nd edition. ISBN: 9781462549863. [p87]

Enders, C. K., Hayes, T., and Du, H. (2018). A comparison of multilevel imputation schemes for random coefficient models: fully conditional specification and joint model imputation with random covariance matrices. *Multivariate Behavioral Research*, 53(5):695–713. DOI: 10.1080/00273171.2018.1477040. [p27]

Erler, N. S., Rizopoulos, D., and Lesaffre, E. M. E. H. (2021). JointAI: Joint analysis and imputation of incomplete data in R. *Journal of Statistical Software*, 100(20):1–56. DOI: 10.18637/jss.v100.i20. [p44]

Gan, Y., Yang, Z., and Mei, W. (2019). *ed50: Estimate ED50 and Its Confidence Interval.* R package version 0.1.1. [p67]

Gelman, A., Hill, J., and Yajima, M. (2012). Why we (usually) don't have to worry about multiple comparisons. *Journal of Research on Educational Effectiveness*, 5(2):189–211. DOI: 10.1080/19345747.2011.618213. [p79]

Gelman, A. and Loken, E. (2013). The garden of forking paths: Why multiple comparisons can be a problem, even when there is no "fishing expedition" or "p-hacking" and the research hypothesis was posited ahead of time. *Department of Statistics, Columbia University*, 348:1–17. [p79]

Glynn, R. J., Laird, N. M., and Rubin, D. B. (1986). *Selection Modeling Versus Mixture Modeling with Nonignorable Nonresponse*, pages 115–142. Springer, New York, NY. DOI: 10.1007/978-1-4612-4976-$4_1$0. [p40]

Gower-Page, C. and Noci, A. (2022). *rbmi: Reference Based Multiple Imputation.* R package version 1.2.3. [p41]

Greenland, S. (2021). Noncollapsibility, confounding, and sparse-data bias. part 2: What should researchers make of persistent controversies about the odds ratio? *Journal of Clinical Epidemiology*, 139:264–268. DOI: 10.1016/j.jclinepi.2021.06.004. [p71]

Greenland, S., Pearl, J., and Robins, J. M. (1999). Confounding and collapsibility in causal inference. *Statistical Science*, 14(1):29–46. DOI: 10.1214/ss/1009211805. [p71]

Groenwold, R. H., Moons, K. G., Peelen, L. M., Knol, M. J., and Hoes, A. W. (2011). Reporting of treatment effects from randomized trials: A plea for multivariable risk ratios. *Contemporary Clinical Trials*, 32(3):399–402. DOI: 10.1016/j.cct.2010.12.011. [p70]

Grund, S., Lüdtke, O., and Robitzsch, A. (2018). Multiple imputation of missing data at level 2: A comparison of fully conditional and joint modeling in multilevel designs. *Journal of Educational and Behavioral Statistics*, 43(3):316–353. DOI: 10.3102/1076998617738087. [p27]

Grund, S., Robitzsch, A., and Luedtke, O. (2021). *mitml: Tools for Multiple Imputation in Multilevel Modeling.* R package version 0.4-3. [p43]

Harrer, M., Cuijpers, P., A, F. T., and Ebert, D. D. (2021). *Doing Meta-Analysis With R: A Hands-On Guide.* Chapman & Hall/CRC Press, Boca Raton, FL and London, 1st edition. ISBN 978-0-367-61007-4. [p6, 58]

Heckman, J. J. (1979). Sample selection bias as a specification error. *Econometrica*, 47(1):153–161. DOI: 10.2307/1912352. [p40]

Hengartner, M. P. (2017). Methodological flaws, conflicts of interest, and scientific fallacies: implications for the evaluation of antidepressants' efficacy and harm. *Frontiers in Psychiatry*, 8:1–7. DOI: 10.3389/fpsyt.2017.00275. [p58]

Henry, L. and Wickham, H. (2020). *purrr: Functional Programming Tools.* R package version 0.3.4. [p36, 48]

Holm, S. (1979). A simple sequentially rejective multiple test procedure. *Scandinavian Journal of Statistics*, 6(2):65–70. [p78]

Iddrisu, A.-K. and Gumedze, F. (2019). An application of a pattern-mixture model with multiple imputation for the analysis of longitudinal trials with protocol deviations. *BMC Medical Research Methodology*, 19:1–23. DOI: 10.1186/s12874-018-0639-y. [p41]

Jacobson, N. S. and Truax, P. (1992). Clinical significance: a statistical approach to defining meaningful change in psychotherapy research. *Journal of Consulting and Clinical Psychology*, 59(1):12–19. DOI: 10.1037//0022-006x.59.1.12. [p61]

Jiang, H., Kulkarni, P. M., Mallinckrodt, C. H., Shurzinske, L., Molenberghs, G., and Lipkovich, I. (2017). Covariate adjustment for logistic regression analysis of binary clinical trial data. *Statistics in Biopharmaceutical Research*, 9(1):126–134. DOI: 10.1080/19466315.2016.1234973. [p70]

Kahan, B. C., Jairath, V., Doré, C. J., and Morris, T. P. (2014). The risks and rewards of covariate adjustment in randomized trials: an assessment of 12 outcomes from 8 studies. *Trials*, 15(1):1–7. DOI: 10.1186/1745-6215-15-139. [p51]

Kahan, B. C. and Morris, T. P. (2012). Reporting and analysis of trials using stratified randomisation in leading medical journals: review and reanalysis. *BMJ*, 345. DOI: 10.1136/bmj.e5840. [p52]

Kahan, B. C., Rushton, H., Morris, T. P., and Daniel, R. M. (2016). A comparison of methods to adjust for continuous covariates in the analysis of randomised trials. *BMC Medical Research Methodology*, 16(1):1–10. DOI: 10.1186/s12874-016-0141-3. [p51]

Kenward, M. G. and Roger, J. H. (1997). Small sample inference for fixed effects from restricted maximum likelihood. *Biometrics*, 53(3):983–997. DOI: 10.2307/2533558. [p81]

Knottnerus, J. A. and Bouter, L. M. (2001). The ethics of sample size:: Two-sided testing and one-sided thinking. *Journal of Clinical Epidemiology*, 54(2):109–110. DOI: 10.1016/s0895-4356(00)00276-6. [p58]

Lehmann, E. L. (2011). Latin squares. In *Fisher, Neyman, and the Creation of Classical Statistics*, chapter 5.2. Springer. DOI: 10.1007/978-1-4419-9500-1. [p1]

Li, G., Taljaard, M., van den Heuvel, E. R., Levine, M. A., Cook, D. J., Wells, G. A., Devereaux, P. J., and Thabane, L. (2017). An introduction to multiplicity issues in clinical trials: the what, why, when and how. *International Journal of Epidemiology*, 46(2):746–755. DOI: 10.1093/ije/dyw320. [p79]

Lilienfeld, S. O. (2007). Psychological treatments that cause harm. *Perspectives on Psychological Science*, 2(1):53–70. DOI: 10.1111/j.1745-6916.2007.00029.x. [p58]

Little, R. J. (2014). Selection model (missing data). *Wiley StatsRef: Statistics Reference Online*, pages 1–5. DOI: 10.1002/9781118445112.stat07871. [p40]

Little, R. J. A. (1988). A test of missing completely at random for multivariate data with missing values. *Journal of the American Statistical Association*, 83(404):1198–1202. DOI: 10.2307/2290157. [p25]

Liublinska, V. and Rubin, D. B. (2014). Sensitivity analysis for a partially missing binary outcome in a two-arm randomized clinical trial. *Statistics in Medicine*, 33(24):4170–4185. DOI: 10.1002/sim.6197. [p40]

Lunn, D., Jackson, C., Best, N., Thomas, A., and Spiegelhalter, D. (2012). *The BUGS book. A practical introduction to Bayesian analysis*. Chapman Hall, 1st edition. ISBN: 9781584888499. [p38]

Lydersen, S. (2021). Why are one-sided hypothesis tests rarely used? *Tidsskrift for Den norske legeforening*. DOI: 10.4045/tidsskr.21.0111. [p58]

Madley-Dowd, P., Hughes, R., Tilling, K., and Heron, J. (2019). The proportion of missing data should not be used to guide decisions on multiple imputation. *Journal of Clinical Epidemiology*, 110:63–73. DOI: 10.1016/j.jclinepi.2019.02.016. [p55]

Magnusson, K. (2019). Why linear mixed-effects models are probably not the solution to your missing data problems. https://web.archive.org/web/20220626101821/https://rpsychologist.com/lmm-slope-missingness/. Accessed on: March 23, 2023. [p84]

Mallinckrodt, C. H., Lane, P. W., Schnell, D., Peng, Y., and Mancuso, J. P. (2008). Recommendations for the primary analysis of continuous endpoints in longitudinal clinical trials. *Drug Information Journal*, 42(4):303–319. DOI: 10.1177/009286150804200402. [p80]

Manea, L., Gilbody, S., and McMillan, D. (2012). Optimal cut-off score for diagnosing depression with the Patient Health Questionnaire (pHQ-9): a meta-analysis. *Canadian Medical Association Journal*, 184(3):191–196. DOI: 10.1503/cmaj.110829. [p66]

Manos, R. C., Kanter, J. W., and Luo, W. (2011). The behavioral activation for depression scale–short form: development and validation. *Behavior Therapy*, 42(4):726–739. DOI: 10.1016/j.beth.2011.04.004. [p16]

McAleavey, A. (2021). When (not) to rely on the reliable change index. DOI: 10.31219/osf.io/3kthg. [p65]

McGrath, K. (2023). *RefBasedMI: Reference-Based Imputation for Longitudinal Clinical Trials with Protocol Deviation*. R package version 0.1.0. [p41]

McKay, D. and Jensen-Doss, A. (2021). Harmful treatments in psychotherapy. *Clinical Psychology: Science and Practice*, 28(1):2–4. DOI: 10.1037/cps0000023. [p58]

Mendes, D., Alves, C., and Batel-Marques, F. (2017). Number needed to treat (NNT) in clinical literature: an appraisal. *BMC Medicine*, 15(1):1–13. DOI: 10.1186/s12916-017-0875-8. [p63]

Meng, X.-L. (1994). Multiple-imputation inferences with uncongenial sources of input. *Statistical Science*, 9(4):538–558. DOI: 10.1214/ss/1177010269. [p43]

Moher, D., Hopewell, S., Schulz, K. F., Montori, V., Gøtzsche, P. C., Devereaux, P. J., Elbourne, D., Egger, M., and Altman, D. G. (2012). CONSORT 2010 explanation and elaboration: updated guidelines for reporting parallel group randomised trials. *International Journal of Surgery*, 10(1):28–55. DOI: 10.1016/j.ijsu.2011.10.001. [p19, 21]

Molenberghs, G., Thijs, H., Jansen, I., Beunckens, C., Kenward, M. G., Mallinckrodt, C., and Carroll, R. J. (2004). Analyzing incomplete longitudinal clinical trial data. *Biostatistics*, 5(3):445–464. DOI: 10.1093/biostatistics/5.3.445. [p24]

Montgomery, D. C. (2017). *Design and analysis of experiments*. John Wiley & Sons, 9th edition. ISBN 9781119113478. [p50]

Moore, D. F. (2016). *Applied survival analysis using R*, volume 473. Springer, 1st edition. DOI: 10.1007/978-3-319-31245-3. [p61]

Morris, T. P., Walker, A. S., Williamson, E. J., and White, I. R. (2022). Planning a method for covariate adjustment in individually randomised trials: a practical guide. *Trials*, 23(1). DOI: 10.1186/s13063-022-06097-z. [p51, 70, 71]

Morris, T. P., White, I. R., and Royston, P. (2014). Tuning multiple imputation by predictive mean matching and local residual draws. *BMC Medical Research Methodology*, 14:1–13. DOI: 10.1186/1471-2288-14-75. [p28]

Moyé, L. A. and Tita, A. T. (2002). Defending the rationale for the two-tailed test in clinical research. *Circulation*, 105(25):3062–3065. DOI: 10.1161/01.CIR.0000018283.15527.97. [p58]

Naimi, A. I., Cole, S. R., and Kennedy, E. H. (2017). An introduction to g methods. *International Journal of Epidemiology*, 46(2):756–762. DOI: 10.1093/ije/dyw323. [p71]

Naudet, F., Sakarovitch, C., Janiaud, P., Cristea, I., Fanelli, D., Moher, D., and Ioannidis, J. P. (2018). Data sharing and reanalysis of randomized controlled trials in leading biomedical journals with a full data sharing policy: survey of studies published in The BMJ and PLOS Medicine. *BMJ*, 360. DOI: 10.1136/bmj.k400. [p86]

Navarro, D. (2013). *Learning statistics with R*. Lulu Publishing. [p7]

Owen, A. (2007). The ethics of two-and one-sided hypothesis tests for clinical trials. *Clinical Ethics*, 2(2):100–102. DOI: 10.1258/147775007781029537. [p58]

Permutt, T. (2020). Do covariates change the estimand? *Statistics in Biopharmaceutical Research*, 12(1):45–53. DOI: 10.1080/19466315.2019.1647874. [p51]

Picco, L., Abdin, E., Chong, S. A., Pang, S., Shafie, S., Chua, B. Y., Vaingankar, J. A., Ong, L. P., Tay, J., and Subramaniam, M. (2016). Attitudes toward seeking professional psychological help: Factor structure and socio-demographic predictors. *Frontiers in Psychology*, 7:547. DOI: 10.3389/fpsyg.2016.00547. [p17]

Quartagno, M. and Carpenter, J. (2022a). *jomo: A package for Multilevel Joint Modelling Multiple Imputation*. [p27]

Quartagno, M. and Carpenter, J. R. (2022b). Substantive model compatible multilevel multiple imputation: A joint modeling approach. *Statistics in Medicine*, 41(25):5000–5015. DOI: 10.1002/sim.9549. [p43]

Quartagno, M., Grund, S., and Carpenter, J. (2019). jomo: a flexible package for two-level joint modelling multiple imputation. *The R Journal*, 11(2):205–228. DOI: 10.32614/RJ-2019-028. [p27]

Radloff, L. S. (1977). The CES-D scale: A self-report depression scale for research in the general population. *Applied Psychological Measurement*, 1(3):385–401. DOI: 10.1177/014662167700100306. [p16]

Revelle, W. (2022). *psych: Procedures for Psychological, Psychometric, and Personality Research*. Northwestern University, Evanston, Illinois. R package version 2.2.3. [p18]

Robins, J. (1986). A new approach to causal inference in mortality studies with a sustained exposure period—application to control of the healthy worker survivor effect. *Mathematical Modelling*, 7(9-12):1393–1512. DOI: 10.1016/0270-0255(86)90088-6. [p71]

Robinson, L., Delgadillo, J., and Kellett, S. (2020). The dose-response effect in routinely delivered psychological therapies: A systematic review. *Psychotherapy Research*, 30(1):79–96. DOI: 10.1080/10503307.2019.1566676. [p41]

Robitzsch, A. and Grund, S. (2022). *miceadds: Some Additional Multiple Imputation Functions, Especially for 'mice'*. R package version 3.12-26. [p29]

Rothman, K. J. (1990). No adjustments are needed for multiple comparisons. *Epidemiology*, 1(1):43–46. [p79]

Rubin, D. B. (1976). Inference and missing data. *Biometrika*, 63(3):581–592. DOI: 10.1093/biomet/63.3.581. [p22]

Rubin, D. B. (1996). Multiple imputation after 18+ years. *Journal of the American Statistical Association*, 91(434):473–489. DOI: 10.2307/2291635. [p26]

Rubin, D. B. (2004). *Multiple imputation for nonresponse in surveys*, volume 81. John Wiley & Sons. ISBN 9780471655749. [p26, 44]

Sabanés Bové, D., Dedic, J., Kelkhoff, D., Kunzmann, K., Lang, B. M., Li, L., Stock, C., Wang, Y., and James, D. (2023). *mmrm: Mixed Models for Repeated Measures*. R package version 0.2.2.9013. [p80]

Schafer, J. L. (1997). *Analysis of incomplete multivariate data*. CRC Press, 1st edition. DOI: 10.1201/9780367803025. [p26]

Schauberger, P. and Walker, A. (2021). *openxlsx: Read, Write and Edit xlsx Files*. R package version 4.2.5. [p6]

Schulz, K. F., Altman, D. G., and Moher, D. (2010). CONSORT 2010 statement: updated guidelines for reporting parallel group randomised trials. *Journal of Pharmacology and Pharmacotherapeutics*, 1(2):100–107. DOI: 10.1186/1741-7015-8-18. [p21]

Senn, S. (2005). Dichotomania: an obsessive compulsive disorder that is badly affecting the quality of analysis of pharmaceutical trials. *Proceedings of the International Statistical Institute*, 55:1–13. [p61]

Shalom, J. G. and Aderka, I. M. (2020). A meta-analysis of sudden gains in psychotherapy: Outcome and moderators. *Clinical Psychology Review*, 76:101827. DOI: 10.1016/j.cpr.2020.101827. [p41]

Sjölander, A. and Dahlqwist, E. (2021). *stdReg: Regression Standardization*. R package version 3.4.1. [p71]

Sjölander, A. and Vansteelandt, S. (2019). Frequentist versus bayesian approaches to multiple testing. *European Journal of Epidemiology*, 34:809–821. DOI: 10.1007/s10654-019-00517-2. [p79]

Spitzer, R. L., Kroenke, K., Williams, J. B., and Löwe, B. (2006). A brief measure for assessing generalized anxiety disorder: the GAD-7. *Archives of Internal Medicine*, 166(10):1092–1097. DOI: 10.1001/archinte.166.10.1092. [p66]

Tackney, M. S., Morris, T., White, I., Leyrat, C., Diaz-Ordaz, K., and Williamson, E. (2023). A comparison of covariate adjustment approaches under model misspecification in individually randomized trials. *Trials*, 24(1):1–18. DOI: 10.1186/s13063-022-06967-6. [p51, 52]

Taichman, D. B., Sahni, P., Pinborg, A., Peiperl, L., Laine, C., James, A., Hong, S.-T., Haileamlak, A., Gollogly, L., Godlee, F., Frizelle, F. A., Florenzano, F., Drazen, J. M., Bauchner, H., Baethge, C., and Backus, J. (2017). Data sharing statements for clinical trials: a requirement of the international committee of medical journal editors. *Annals of Internal Medicine*, 167(1):63–65. DOI: 10.7326/M17-1028. [p86]

Tan, P.-T., Cro, S., van Vogt, E., Szigeti, M., and Cornelius, V. R. (2021). A review of the use of controlled multiple imputation in randomised controlled trials with missing outcome data. *BMC Medical Research Methodology*, 21(1):1–17. DOI: 10.1186/s12874-021-01261-6. [p39]

Thijs, H., Molenberghs, G., Michiels, B., Verbeke, G., and Curran, D. (2002). Strategies to fit pattern-mixture models. *Biostatistics*, 3(2):245–265. DOI: 10.1093/biostatistics/3.2.245. [p40]

Thoemmes, F. and Enders, C. K. (2007). A structural equation model for testing whether data are missing completely at random. In *Annual meeting of the American Educational Research Association, Chicago, IL*. [p25]

Twisk, J., de Boer, M., de Vente, W., and Heymans, M. (2013). Multiple imputation of missing values was not necessary before performing a longitudinal mixed-model analysis. *Journal of Clinical Epidemiology*, 66(9):1022–1028. DOI: 10.1016/j.jclinepi.2013.03.017. [p84]

Twisk, J. W. R. (2021). *Analysis of Data from Randomized Controlled Trials: A Practical Guide*. Springer, Cham, Switzerland, 1st edition. DOI: 10.1007/978-3-030-81865-4. [p80, 87]

van Buuren, S. (2007). Multiple imputation of discrete and continuous data by fully conditional specification. *Statistical Methods in Medical Research*, 16(3):219–242. DOI: 10.1177/0962280206074463. [p27]

van Buuren, S. (2018). *Flexible imputation of missing data*. CRC Press, 2nd edition. DOI: 10.1201/9780429492259. [p22, 25, 28, 30, 38, 44, 87]

van Buuren, S., Brand, J. P., Groothuis-Oudshoorn, C. G., and Rubin, D. B. (2006). Fully conditional specification in multivariate imputation. *Journal of Statistical Computation and Simulation*, 76(12):1049–1064. DOI: 10.1080/10629360600810434. [p27]

van Buuren, S. and Groothuis-Oudshoorn, K. (2011a). mice: Multivariate imputation by chained equations in R. *Journal of Statistical Software*, 45:1–67. DOI: 10.18637/jss.v045.i03. [p26, 27]

van Buuren, S. and Groothuis-Oudshoorn, K. (2011b). mice: Multivariate imputation by chained equations in R. *Journal of Statistical Software*, 45(3):1–67. DOI: 10.18637/jss.v045.i03. [p28]

Vansteelandt, S. and Keiding, N. (2011). Invited commentary: G-computation–lost in translation? *American Journal of Epidemiology*, 173(7):739–742. DOI: 10.1093/aje/kwq474. [p71]

Venables, W. N. and Ripley, B. D. (2002). *Modern Applied Statistics with S*. Springer, New York, 4th edition. ISBN 0-387-95457-0. [p4]

Vickerstaff, V., Omar, R. Z., and Ambler, G. (2019). Methods to adjust for multiple comparisons in the analysis and sample size calculation of randomised controlled trials with multiple primary outcomes. *BMC Medical Research Methodology*, 19(1):1–13. DOI: 10.1186/s12874-019-0754-4. [p77]

Vilagut, G., Forero, C. G., Barbaglia, G., and Alonso, J. (2016). Screening for depression in the general population with the Center for Epidemiologic Studies Depression (CES-D): a systematic review with meta-analysis. *PLOS One*, 11(5). DOI: 10.1371/journal.pone.0155431. [p66]

Wang, B., Ogburn, E. L., and Rosenblum, M. (2019). Analysis of covariance in randomized trials: More precision and valid confidence intervals, without model assumptions. *Biometrics*, 75(4):1391–1400. DOI: 10.1111/biom.13062. [p52]

Waring, E., Quinn, M., McNamara, A., Arino de la Rubia, E., Zhu, H., and Ellis, S. (2022). *skimr: Compact and Flexible Summaries of Data*. R package version 2.1.4. [p17]

White, I., Joseph, R., and Best, N. (2020). A causal modelling framework for reference-based imputation and tipping point analysis in clinical trials with quantitative outcome. *Journal of Biopharmaceutical Statistics*, 30(2):334–350. DOI: 10.1080/10543406.2019.1684308. [p40]

White, I. R., Royston, P., and Wood, A. M. (2011). Multiple imputation using chained equations: Issues and guidance for practice. *Statistics in Medicine*, 30(4):377–399. DOI: 10.1002/sim.4067. [p26, 29]

Wickham, H. (2016). *ggplot2: Elegant Graphics for Data Analysis*. Springer-Verlag, New York. DOI: 10.1007/978-0-387-98141-3. [p4]

Wickham, H., Averick, M., Bryan, J., Chang, W., McGowan, L. D., François, R., Grolemund, G., Hayes, A., Henry, L., Hester, J., Kuhn, M., Pedersen, T. L., Miller, E., Bache, S. M., Müller, K., Ooms, J., Robinson, D., Seidel, D. P., Spinu, V., Takahashi, K., Vaughan, D., Wilke, C., Woo, K., and Yutani, H. (2019). Welcome to the Tidyverse. *Journal of Open Source Software*, 4(43):1686. DOI: 10.21105/joss.01686. [p4]

Wickham, H. and Grolemund, G. (2016). *R for data science: import, tidy, transform, visualize, and model data*. O'Reilly Media, Inc., 2nd edition. ISBN 978-1491910399. [p7, 87]

Williamson, T., Eliasziw, M., and Fick, G. H. (2013). Log-binomial models: exploring failed convergence. *Emerging Themes in Epidemiology*, 10(1):1–10. DOI: 10.1186/1742-7622-10-14. [p69]

Wise, E. A. (2004). Methods for analyzing psychotherapy outcomes: A review of clinical significance, reliable change, and recommendations for future directions. *Journal of Personality Assessment*, 82(1):50–59. DOI: 10.1207/s15327752jpa8201$_1$0. [p65]

Zigmond, A. S. and Snaith, R. P. (1983). The hospital anxiety and depression scale. *Acta Psychiatrica Scandinavica*, 67(6):361–370. DOI: 10.1111/j.1600-0447.1983.tb09716.x. [p16]

Index

A
Absolute Risk Reduction, 76
ANCOVA, 50, 53, 55, 67, 68, 71, 80, 86
Antilog, 68, 69
Argument (Function), 7, 8
Assignment Operator, 12
Auxiliary Variable, 29, 32, 34, 54
Average Treatment Effect, 50, 59, 70, 71

B
Base R, 4
Bonferroni Correction, 78
By-Group Imputation, 36

C
Center for Epidemiological Studies' Depression Scale (CES-D), 16, 19, 50
Class (R), 9
Cluster-Randomized Trial, 87
Cohen's d, 58–60
Compatibility (Imputation), 43
Complete Case Analysis, 52
Comprehensive R Archive Network (CRAN), 4, 5
Congeniality (Imputation), 43
CONSORT, 19–21, 84
Controlled Multiple Imputation, 40
Convergence, 38
Correlation, 47
Covariate, 50–52, 59, 70, 71
Cross-Over Trial, 87

D
Data Frame, 10
Default Arguments, 8
Dichotomania, 61
Documentation, 8
Donors (Predictive Mean Matching), 28

E
Effect Coding, 50
Effect Size, 58, 59
Effective Dose 50, 67
Environment (R), 9
Estimand, 49, 52, 70
Exponential Distribution, 67
Eyeballing, 18

F
Family-Wise Error Rate, 77
Fisher, Ronald A., 1, 47
For Loop, 48
Fraction of Missing Information Due to Non-Response, 46, 55
Fully Conditional Specification, 26, 27
Function, 7
Functional Programming, 7, 48

G
G-computation, 52, 71
Generalized Estimating Equations, 80
Generalized Linear Model, 67, 68
Github, 16, 41
Graphical User Interface (GUI), 3, 15

H
Histogram, 18
Holm-Bonferroni Correction, 78

I
Ignorable Missing Data, 24, 25
Imputation Matrix, 34
Incidence Rate Ratio, 68
Influx, 30, 31
Integrated Development Environment (IDE), 3

J
Joint Modeling, 26, 27
Jump-To-Reference (J2R), 40, 41, 56, 57

K
Kenward-Roger Correction, 81

L
Linear Regression, 50
List, 11
Listwise Deletion, 22
Little's MCAR Test, 25
Logical Operator, 12
Logistic Regression, 31, 67, 69

M
MAR (Missing At Random), 22, 23, 25, 26
Matrix, 10
Maximum Likelihood Estimation, 67
MCAR (Missing Completely At Random), 21–23, 25, 26
Minimally Important Difference, 66
Missing At Random (MAR), 84
Missing Not At Random (MNAR), 39
Mixed Model, 80, 84
Mixed-Model Repeated Measures, 80
MNAR (Missing Not At Random), 22, 23, 25
Monotone Missingness, 29
Multiplicity, 77, 79
Multivariate Imputation by Chained Equations (MICE), 26, 27, 42

N
Negative Binomial Model, 67
Non-Collapsibility, 70
Number Needed To Harm, 76
Number Needed To Treat, 63, 76

O
Object (R), 9

Odds Ratio, 68, 70
Offset, 67
One-Sided Testing, 57
Open Science Framework, 86
Outflux, 30, 31

P
Package, 4
Pipe Operator, 12
Poisson Regression, 67, 72
Position Matching (Function), 8
Power, Statistical, 51
Predictive Mean Matching, 28
Predictor Matrix, 34
Proportion of Usable Cases, 34

R
Reference-Based Imputation, 40
Relative Increase in Variance, 45, 55
Reliability Coefficient, 62
Reliable Change Index, 61, 62, 65, 66
Risk Difference, 72
Risk Ratio, 71, 74
Robust Variance Estimation, 72
RStudio, 3, 5, 6
Rubin's Rules, 44, 45
Rubin, Donald, 22

S
Sandwich Estimator, 72
Scalar, 9, 74
Selection Model, 39
Standardization (Regression), 52, 71
Standardized Mean Difference, 58
STATA, 41, 87
Stepped-Wedge Trial, 87
Stratified Randomization, 52
Sum of Squares, 50

T
Test Statistic, 47
Tidyverse, 4, 5, 36, 41, 48, 87
Tipping Point Analysis, 39
Transportability, 75

V
Variance Decomposition, 50
Vector, 9

W
Working Directory, 6
